# Head and neck cancer mortality by gender, region and ethnicity: a population-based study in Brazil

**DOI:** 10.1016/j.lana.2025.101306

**Published:** 2025-11-29

**Authors:** Matheus de Abreu, Maria do Rosario Dias de Oliveira Latorre, Maria Paula Curado

**Affiliations:** a Department of Epidemiology and Statistics, International Research Center, A.C.Camargo Cancer Center, São Paulo, SP, Brazil; b Department of Epidemiology, School of Public Health, University of São Paulo, São Paulo, SP, Brazil

**Keywords:** Head and neck neoplasms, Mortality, Squamous cell carcinoma of head and neck

## Abstract

**Background:**

Brazil exhibits the highest mortality rates for oral cavity (OCC), oropharyngeal (OPC), and laryngeal (LC) cancers in South-America. This study aims to analyze mortality trends in head and neck cancers (HNC) across the Brazilian population over 44-year period.

**Methods:**

A time-series ecological study was conducted using OCC, OPC, LC mortality data. Age-period-cohort (APC) effects and the average annual percent change (AAPC) were estimated for each cancer subsite by gender, regions, ethnicity.

**Findings:**

From 1980 to 2023, 303,882 HNC deaths were recorded among adults ≥40 years. LC predominated (45.4%), followed by OCC (30.5%), OPC (24.1%). Mortality rates were higher in men, LC showing the highest. After 2000, mortality declines for LC (RR: 0.98 [0.98–1.00 CI 95%]) in men, while women increasing mortality for OPC (RR: 1.12 [1.04–1.21]), OCC (RR: 1.08 [1.02–1.15]). Men born after-1955 showed reduced RR for all subsites, women exhibited for LC. The Northeast region showed an increasing trend across all age groups and cancer subsites. OPC presented an increase in mortality in all regions, in contrast to LC and OCC, which declined in the South and Southeast. White men had a decreasing trend for LC (AAPC −1.62 [−1.99 to −1.26]), OCC (AAPC −1.01 [−1.31 to −0.71]), OPC (AAPC −0.60 [−0.98 to −0.24]), whereas Brown showed an increasing for both genders in all subsites.

**Interpretation:**

Gender, ethnicity, and geographic location are associated with HNC mortality in Brazil. White men and developed regions showed the most substantial improvements in mortality, while brown, women, and people in the North/Northeast exhibited concerning increases. OPC, although with the lowest rates, shows a growing mortality trend nationwide.

**Funding:**

CAPES-Brazil.

**Research in context**

**Evidence before this study**

We searched PubMed and Google Scholar for longitudinal studies reporting head and neck cancer (HNC) mortality rates and trends in the Brazilian population. We used the following search terms (“mortality” AND (“head and neck cancer” OR “oropharyngeal cancer” OR “laryngeal cancer” OR “oral cancer” OR “mouth cancer”) AND “Brazil”). Our search was limited to longitudinal studies among adults which were published before October 29, 2025, without language limitation. The search identified 28 studies. Most studies focused on oral cavity and/or oropharyngeal cancer mortality. Ten studies analyzed specific locations in Brazil, 16 studies examined mortality trends by regions, and only 2 analyzed mortality by ethnicity. Six studies applied the age–period–cohort approach, and 11 used the average annual percent change method. None of the national-level studies applied both approaches with stratification by gender, Brazilian regions, and ethnicity for the three anatomical subsites (oral cavity, oropharynx, and larynx). HNC remains a leading cause of cancer mortality in Brazil. Factors such as age, gender, ethnicity, and lifestyle changes are likely related to the recent increasing trends in HNC mortality. Studies conducted with Brazilian data have analyzed individual HNC subsites in selected geographic areas or over shorter time periods. However, there is still a lack of nationwide studies that consider gender, ethnicity and geographic location by anatomical subsite.

**Added value of this study**

The present study is the first to analyze mortality rates and trends in HNC over a 44-year period in Brazil, considering age, gender, ethnicity and geographic region. Our results showed a growing mortality trend of oropharyngeal cancer, regardless of any sociodemographic characteristics. From oral cavity and laryngeal cancer, we observed marked geographic inequalities in mortality, while the South and Southeast regions showed a decline, the Northeast and Central-West regions experienced an increase. Despite the positive impact of tobacco control policies in reducing tobacco consumption nationwide, only men appeared to benefits from these behavioral changes in terms of HNC mortality. The reductions were observed primarily among White men, whereas Research in Context Brown individuals of both genders experienced the highest increases in mortality across all subsites. These observed mortality trends indicate that gender, ethnicity, and geographic location are associated with the recent rise in HNC mortality in Brazil.

**Implications of all the available evidence**

Our findings indicate that HNC mortality remains higher among men in economically productive age groups. These increases in mortality observed in the Northeast and Central-West regions may have substantial impacts on local economies. However, the most pronounced increases occurred among women. The absence of targeted public health policies, combined with changes in social dynamics and behavioral patterns among women, may have contributed to these unfavorable mortality trends. Promoting gender equity in prevention and early detection initiatives is essential to reduce HNC mortality among women. This study also demonstrated that ethnicity is associated with HNC mortality, with Brown individuals showing increased mortality across all subsites. This finding may be partly explained by improved access to diagnosis within this population and by enhanced accuracy of mortality records in regions predominantly composed of Brown and Black individuals, highlighting the importance of maintaining high-quality and comprehensive vital registration systems for cancer surveillance and control. Overall, this study provides valuable insights into the emerging epidemiological landscape of HNC mortality in Brazil. Prevention and early diagnosis strategies, adapted to this new reality, could be essential to contain future increases in mortality from HNC in the country.

## Introduction

According to the Brazilian National Cancer Institute (INCA), an estimated 22,890 new cases of head and neck cancer (HNC) are expected annually in Brazil during the 2023–2025 period.^1^ Among HNC subtypes, oral cavity cancer (OCC) is the most common, ranking as the fifth most frequent cancer among men in the country.^1^ Mortality rates for HNC differ by subsite in the country. Overall, 11,334 deaths from this group of neoplasms were recorded in 2023. The highest rates were observed among men, with the larynx cancer (LC) (3.05 per 100,000 inhabitants) and oropharynx (OPC) (2.09 per 100,000) showing the highest mortality.^2^

Despite the growing global burden of HNC, comprehensive Brazilian mortality trend analyses remain limited. Few studies have examined HNC mortality trends in Brazil, often anatomically restricted–focusing primarily on oral cavity and oropharyngeal subsites–or geographically limited to specific Brazilian states. Considering that mortality is an important indicator for cancer control policies and disparities in cancer healthcare have been observed among Brazilian regions, our study aims to assess nationwide mortality rates and trends over a 44-year period in order to identify the burden of HNC mortality across Brazilian regions and Brazil as a whole as well as disparitires by gender and ethnicity.

## Methods

A times-series ecological study was conducted to analyzed rates and trends in head and neck cancer mortality in Brazil from 1980 to 2023. Mortality data were obtained from the Mortality Information System (SIM) of the Brazilian Ministry of Health, available through the DATASUS TabNet platform (https://datasus.saude.gov.br/informacoes-de-saude-tabnet/). For the period 1980 to 1995, mortality data were coded using the International Classification of Diseases, ninth edition (ICD-9). In this study, oral cavity cancer was defined using codes C140, C141, C143, C144 and C145; oropharyngeal cancer by C146; and laryngeal cancer by C161. For the period 1996 to 2023, data were coded using the tenth edition (ICD-10), and we defined oral cavity cancer as C00, C02, C03, C04, C05, and C06; oropharyngeal cancer as C01, C09, and C10; and laryngeal cancer as C32. Population data for the period from 1980 to 1999 were based on census data and intercensal projections (https://datasus.saude.gov.br/populacao-residente) and for the period from 2000 to 2023, population estimates were used, obtained from the Department of Monitoring and Evaluation of the Brazilian Unified Health System (DEMAS) (https://www.gov.br/saude/pt-br/composicao/seidigi/demas/dados-populacionais).

**Fig. 1:**
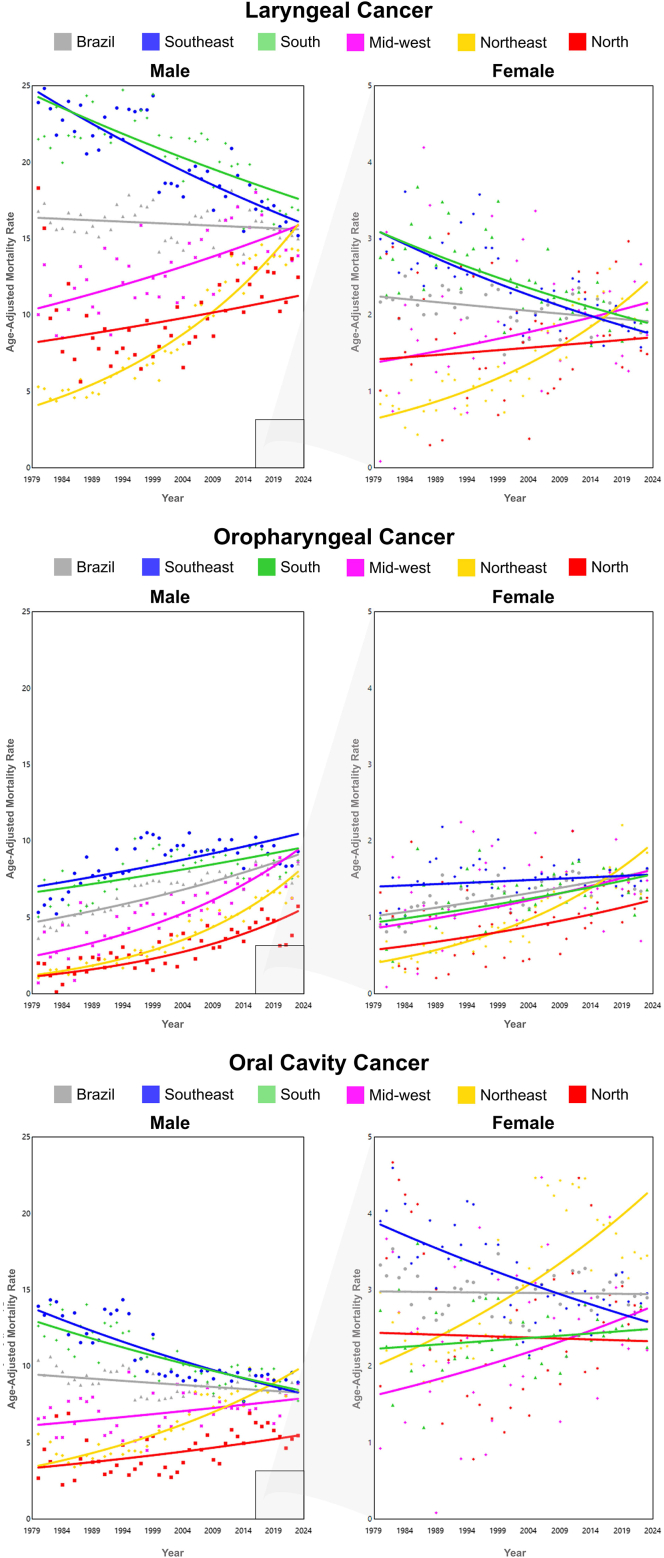
Average annual percent change (AAPC) in mortality from laryngeal, oropharyngeal, and oral cavity cancer by gender in Brazil and Brazilian regions, 1980–2023.

Information on individuals’ race/ethnicity was not available in the TabNet platform. For these analyses, mortality data were directly extracted from the public FTP folder of DATASUS (ftp://ftp.datasus.gov.br/dissemin/publicos/SIM/CID10/DORES/). The classification of the Brazilian Institute of Geography and Statistics (IBGE) was adopted, in which race/skin color categories include White, Black, Brown (“pardo”), Yellow (Asian descent), and Indigenous.^3^ In Brazilian demographic studies, “pardo” is commonly interpreted as mixed race. For consistency, the term “ethnicity” is used throughout the manuscript to refer to these categories, with analyses restricted to White, Black, and Brown, according to the data available from DATASUS. Due to the number of missing data, the selected period for analysis was 2000–2023. Data were coded using the tenth edition (ICD-10), and we defined oral cavity cancer as C003, C004, C005, C006, C008, C009, C020, C021, C022, C023, C028, C029, C030, C031, C039, C040, C041, C048, C049, C050, C058, C059, C060, C061, C062, C068 and C069; oropharyngeal cancer as C019, C024, C051, C052, C058, C090, C091, C098, C099, C101, C102, C103, C104, C108 and C109; and laryngeal cancer as C320, C321, C322, C323, C328 and C329. Population data by ethnicity were obtained from the 2000, 2010, and 2022 censuses, extracted from the SIDRA–IBGE Automatic Recovery System (https://sidra.ibge.gov.br/). To estimate intercensal values, the proportion of each age group relative to the total population in the census years was calculated. A recursive linear interpolation of proportions was applied, estimating values based on the average of those observed in the preceding and subsequent censuses until all missing data were filled. For years following the last census, the proportion from the closest census was assumed for estimation. Absolute estimates were derived by multiplying the interpolated proportions by the annual total population projections provided by DEMAS (https://www.gov.br/saude/pt-br/composicao/seidigi/demas/dados-populacionais). Smoothing of the population estimates over the years was applied using Generalized Additive Models (GAM) to obtain a more stable trend in the data using mgcv package–R version 4.4.1 (Fig. 1 [Supplementary Material]). Death records with unknown age were excluded, proportion can be found in Table S1 [Supplementary Material]. Age groups were organized in 5-year intervals. Mortality and population data for individuals younger than 35 years were excluded due to the low incidence of the disease in this age group. The data were collected between April and October, 2025.

Deaths attributed to ill-defined causes (780–799 of the ICD-9 and R00-R99 of the ICD-10) were redistributed using the World Health Organization (WHO) proportional redistribution methodology.^4^ For each year–sex–age–region stratum, external causes were excluded and the fraction of deaths from the studied cancer subsites among all cancer deaths was calculated. This fraction was then multiplied by the total number of ill-defined causes in that stratum, applying a 50% weight, and the resulting values were added to the site-specific cancer deaths. Unspecified site cancer codes (e.g., C76, C79, C80), which represent neoplasms without indication of the primary location, were fully redistributed (weight = 1.0). These deaths were reassigned proportionally to specific cancer types, based on the distribution of defined cancer sites provided in the Global Burden of Disease (GBD)^5^ stratified by year, sex, age, and region. The reassigned cases were then added to the database already corrected for ill-defined causes. This methodology has been applied and discussed in previous studies.^6^, ^7^, ^8^ The datasets were extracted in CSV format and standardized using the R (version 4.4.1) for age-period-cohort and average annual percent change (AAPC) analyses.

Age-period-cohort models were developed for each cancer subsite (larynx, oropharynx, and oral cavity), stratified by gender, Brazilian region and ethnicity. Age groups were categorized into 9 five-year intervals (40–44 to 80+ years). The period were divided into nine five-year blocks Birth cohorts were calculated from the difference between period and age group, with the earliest cohort in 1900 and the latest in 1980, totaling 17 cohorts. Model fit was assessed through *deviance* statistics. The best-fitting models were selected based on lowest *deviance*, (p < 0.05), and adequate fit as indicated by *deviance* close to residual degrees of freedom. Poisson regression models with APC, Ad-P-C, AP-C, or AC-P parameterizations were fitted to assess the effects of age, period, and birth cohort, when applicable. Initially, the reference categories were selected based on the midpoint's values of the series (period 2000 and birth cohort 1940). After checking model, it was observed that adopting the 1955 birth cohort, which consistently presented the highest relative risks across subgroups, improved the interpretability of results. All models were specified as factor models. The association measures generated by the models were mortality rates and relative risks, both reported with 95% confidence intervals (95% CI). Data processing and analysis were performed using the readxl, tidyverse, and Epi packages–R (version 4.4.1). Graphs were created by extracting the age, period and cohort effects from each model and grouping them by gender for comparison, using the ggplot2, gridExtra and grid packages–R (version 4.4.1).

The AAPC with 95% CI was calculated to determine the weighted average of annual percent changes across all segments in the final model and age-standardized mortality rates (ASMR). Age-specific mortality rates (ASpMR) and AAPCs were estimated for all age groups by cancer subsites (larynx, oropharynx, oral cavity) for each Brazilian region, ethnicity and Brazil as a whole. Mortality rates were calculated using the direct method, as the number of cancer deaths divided by the resident population of the corresponding group, and expressed per 100,000 persons per year. The standard population used was based on Segi's world standard population. Trends were considered statistically significant (either increasing or decreasing) when p < 0.05. All analyses were performed using the National Cancer Institute's Joinpoint Regression Program (version 5.3.0.0). For visualization, six graphs were created (one for each cancer site by gender) using the dplyr, ggplot2, gridExtra, and grid packages–R (version 4.4.1).

This study followed the STROBE reporting guideline for observational studies (Supplementary Material).

### Ethical approval

This study was based on a secondary analysis of publicly available, de-identified population-based data. Therefore, institutional ethics approval was not required. The authors state that all research conducted in this study adhered to the highest ethical standards, ensuring integrity in research practices and compliance with institutional and legal requirements.

### Role of the funding source

The funder of the study had no role in study design, data collection, data Formal analysis, data interpretation, or writing of the report.

## Results

From 1980 to 2023, occurred 303,882 deaths of head and neck cancer recorded among adults. Of these, 137,976 (45.4%) were due to LC (120,979 in men and 16,997 in women); 92,665 (30.5%) from OCC (70,556 men and 22,099 women); and 73,241 (24.1%) from OPC (61,917 men and 11,324 women) (Table S3 [Supplementary Material]). The highest mortality rate in Brazil as a whole was observed in men for LC (16.1 per 100,000), followed by OCC (8.9 per 100,000) and OPC (7.2 per 100,000). For HNC in women, mortality rates were 3.0 per 100,000 for OCC, 2.1 per 100,000 for LC and 1.4 for OPC (Table S3 [Supplementary Material]).

The Southeast region had the highest number of records with 74,791 deaths from larynx (54.2%), 48,691 from oral cavity (52.5%), and 41,693 from oral cavity (56.9%). Meanwhile, in the North and Mid-West regions, deaths did not exceed 4.0% and 6.0% of the national total for any subsites in both genders respectively (e.g., LC in both sexes: North, 4747; Mid-West, 7461 records) (Table S4 [Supplementary Material]). The highest mortalities were observed in the Southeast and South, with rates reaching 20.2 per 100,000 for LC in the South, and 9.1 for OPC and 10.3 for OCC in the Southeast among men. Overall, the North region presented the lowest mortality rates for all subsites in both genders (Table S4 [Supplementary Material]).

Regarding ethnicity (data from 2000 to 2023), whites had the highest number of records for all subsites in both genders, followed by browns. Among white men, deaths from LC accounted for more than 50.0% (n = 45,495) of records, while OCC represented 26.3% (n = 23,591). For browns, LC accounted for approximately 45.0% (n = 23,669) and OCC for 28.6% (n = 14,338) (Table S5 [Supplementary Material]). White men had the highest mortality rates in LC (12.1 per 100,000), OPC (5.5 per 100,000) and OCC (6.3 per 100,000). Among women, blacks followed by browns showed the highest mortality rates, reaching approximately 9.0 per 100,000 for LC (Table S5 [Supplementary Material]).

### Laryngeal cancer

#### Trends

**Fig. 2:**
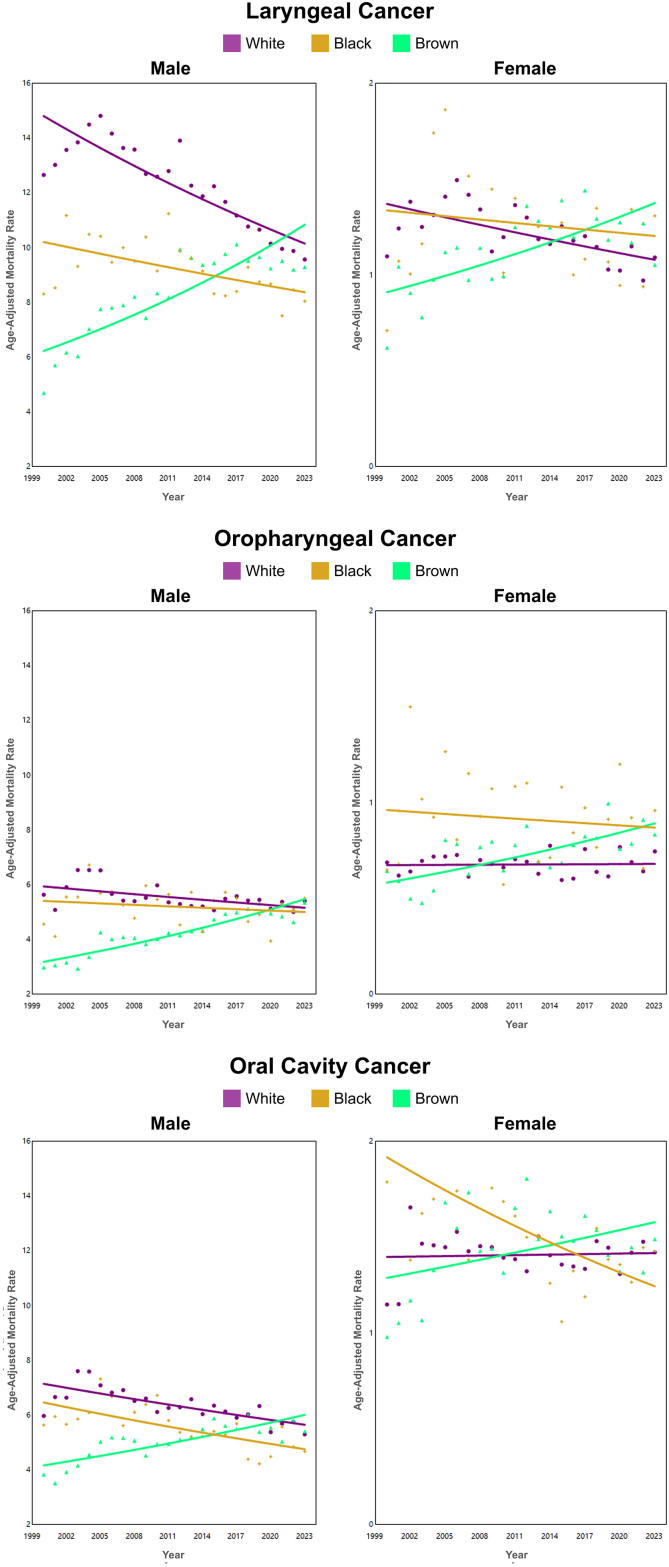
Average annual percent change (AAPC) in mortality from laryngeal, oropharyngeal, and oral cavity cancer by gender and ethnicity in Brazil, 2000–2023.

In Brazil, LC mortality trends among showed a decline in women (AAPC of −0.36 [CI −0.60; −0.12]). For Brazilian regions, the decline in mortality trends was observed only in the South (men AAPC −0.74 [CI 0.93; −0.55]; women AAPC −1.12 [CI −1.40; −0.83) and Southeast (men AAPC −0.97 [CI −1.12; −0.83]; women AAPC −1.28 [CI −1.57; −0.99]), while the highest increasing trends were observed in the Northeast (men AAPC 3.18 [CI 2.92; 3.44]; women AAPC 3.07 [CI 2.56; 3.57]) and Mid-West (men AAPC 0.97 [CI 0.66; 1.29]; women AAPC 1.03 [CI 0.04; 2.03]) (Fig. 1, Table S6 [Supplementary Material]). Regarding ethnicity, a decreasing trend was observed only among whites (men AAPC -1.62; women −1.03) and black men (AAPC -0.85), while an increasing mortality trend was found among browns in both genders (men AAPC 2.43 [CI 1.80; 3.10] and women AAPC 1.81 [CI 1.10; 2.53]) (Fig. 2, Table S7 [Supplementary Material]).

**Fig. 3:**
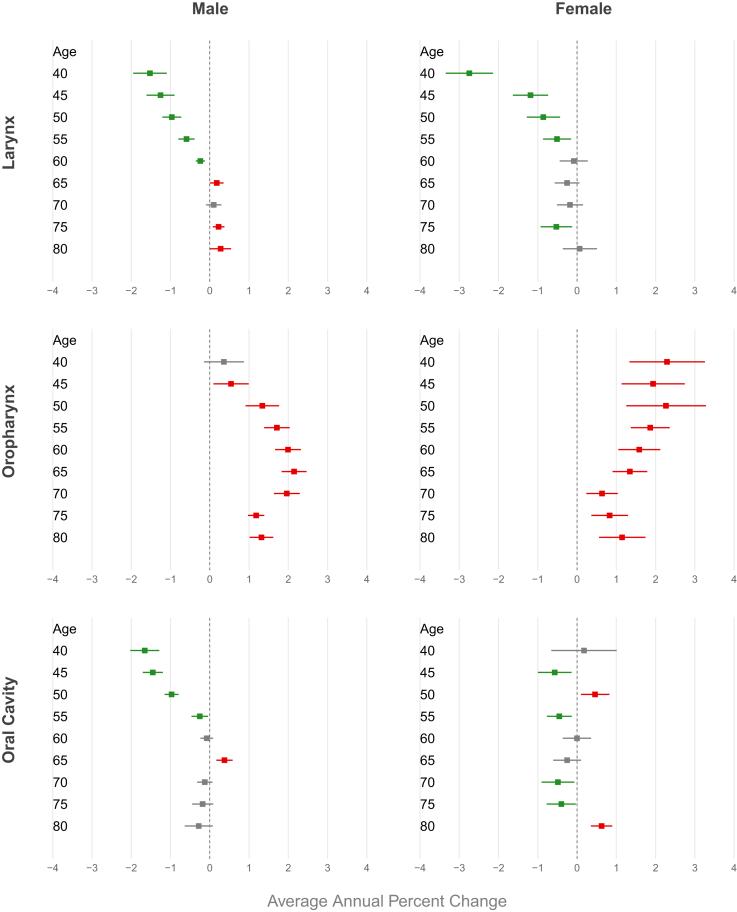
Average annual percent change (AAPC) in mortality from laryngeal, oropharyngeal, and oral cavity cancer by gender and age group in Brazil, 1980–2023.

**Fig. 4:**
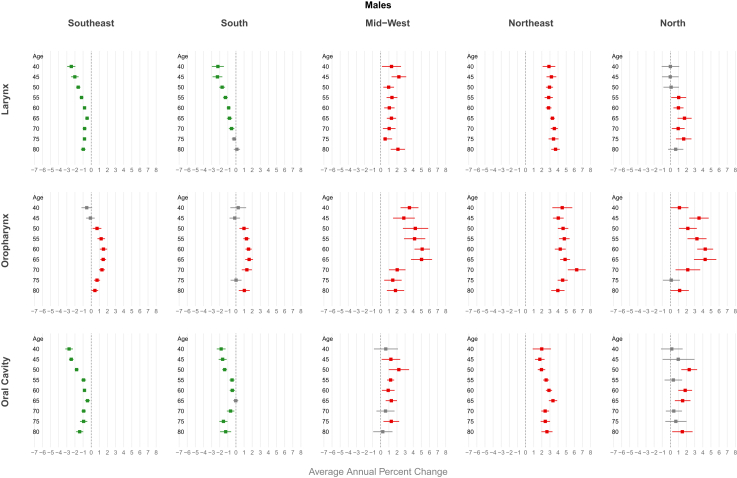
Average annual percent change (AAPC) in mortality from laryngeal, oropharyngeal, and oral cavity cancer in males by age group: Brazilian regions, 1980–2023.

**Fig. 5:**
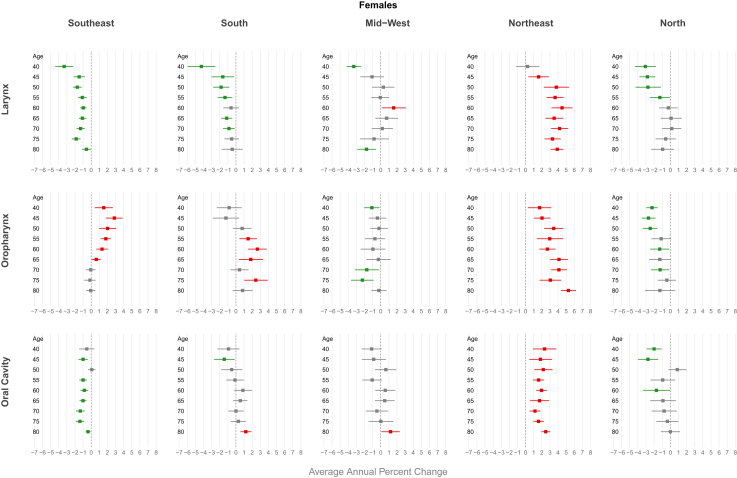
Average annual percent change (AAPC) in mortality from laryngeal, oropharyngeal, and oral cavity cancer in females by age group: Brazilian regions, 1980–2023.

**Fig. 6:**
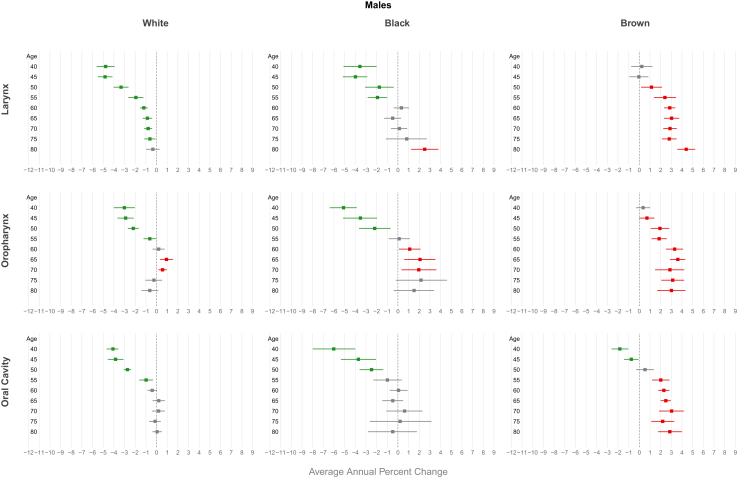
Average annual percent change (AAPC) in mortality from laryngeal, oropharyngeal, and oral cavity cancer in males by age group and ethnicity in Brazil, 2000–2023.

**Fig. 7:**
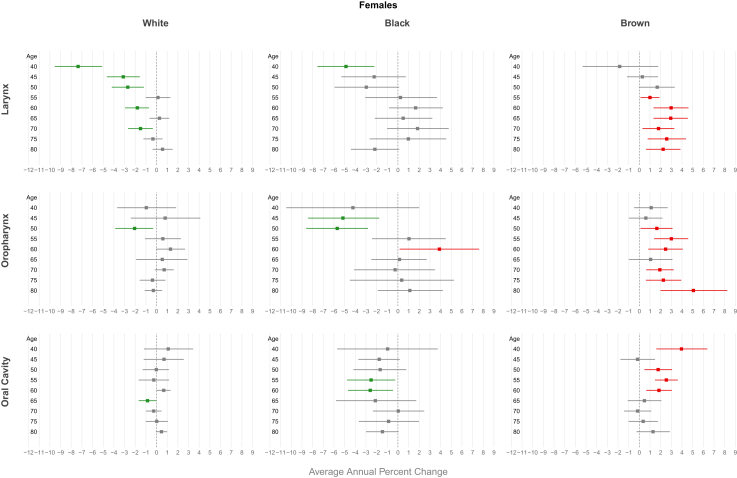
Average annual percent change (AAPC) in mortality from laryngeal, oropharyngeal, and oral cavity cancer in females by age group and ethnicity in Brazil, 2000–2023.

A decreasing trend in mortality was observed in Brazilian men for all age groups under 64 years, with more pronounced declines at younger ages (AAPC −1.52 [CI −1.95; −1.10]) for the 40–44 age group). Same decline for Brazilian women was observed between the ages of 40 and 55, with the highest decline in the 40–44 age group (AAPC −2.74 [CI −3.34; −2.14]) (Fig. 3, Table S8 [Supplementary Material]). Regarding Brazilian regions, a decreasing trend in mortality was observed for all age groups in both genders in the South and Southeast. In contrast, an increasing trend was observed in the Mid-west and in the Northeast among men for all age groups, peaking AAPC 3.54 [CI 3.11; 3.98] for those aged 70–74 in the Northeast. About women increasing trend was observed only in the Northeast (AAPC 4.49 [CI 3.20; 5.80] for those aged 60–64 years) (Figs. 4 and 5, Table S9 [Supplementary Material]). For ethnicity, white males showed a decreasing trend, more pronounced in younger age groups (AAPC −4.77 [CI −5.60; −3.94] for those aged 40–44 years), while an increasing trend were observerd among browns in both genders (AAPC 3.02 [CI 2.31; 3.74] for men and 2.94 [CI 1.32; 4.54] for women aged 65–69 years) (Figs. 6 and 7, Table S10 [Supplementary Material]).

#### Age-period-cohort

**Fig. 8:**
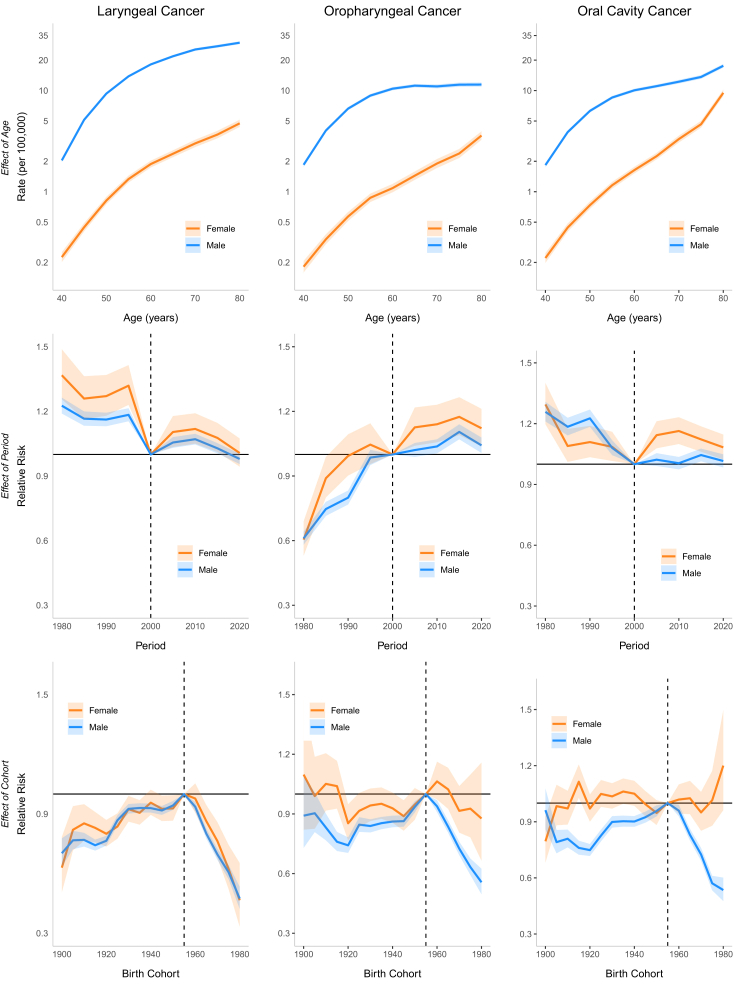
Age, period, and cohort effects on mortality from laryngeal, oropharyngeal, and oral cavity cancers by gender in Brazil, 1980–2023.

**Fig. 9:**
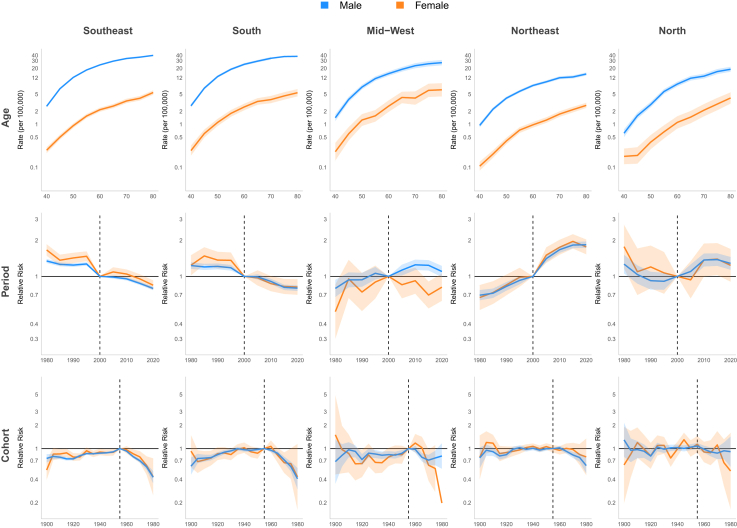
Age, period, and cohort effects on mortality from laryngeal cancer by gender in Brazilian regions, 1980–2023.

**Fig. 10:**
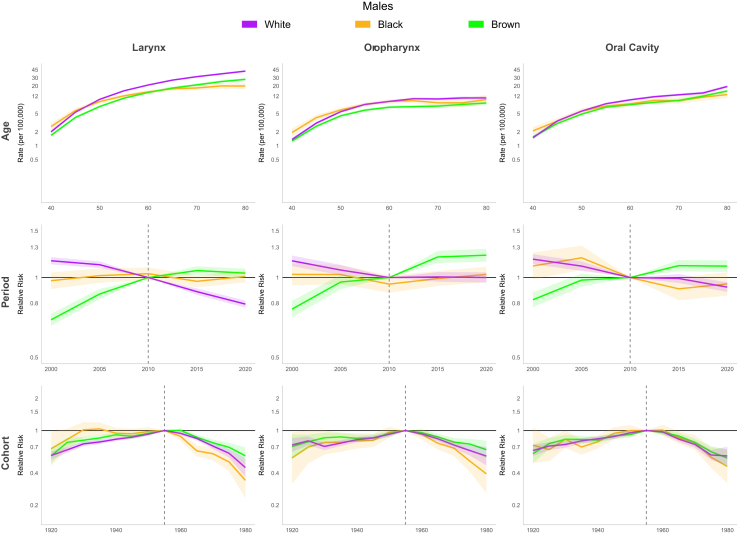
Age, period, and cohort effects on mortality from laryngeal, oropharyngeal, and oral cavity cancers by ethnicity in males in Brazil, 2000–2023.

**Fig. 11:**
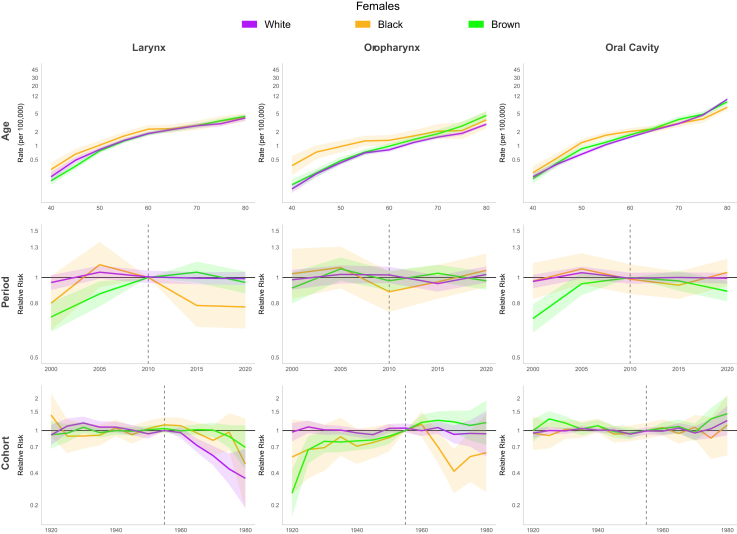
Age, period, and cohort effects on mortality from laryngeal, oropharyngeal, and oral cavity cancers by ethnicity in females in Brazil, 2000–2023.

For Brazil model, the period effect showed a 23% reduction in relative risk (RR) among males and 37% decrease among females between 1980 and 2000. However, this declining trend reversed during the subsequent period (2000–2015), with RR values increasing to 1.07 [CI 1.05; 1.10] for males and 1.12 [CI 1.05; 1.19] for females by 2010. A birth cohort effect was observed, with progressively increasing RR up to the 1955 cohorts, followed by a decline in subsequent cohorts. The lowest RR in the series occurred in the 1980 cohort (men and women: RR 0.47 [CI 0.33; 0.53]) (Fig. 8, Table 1). In the Southeast and South regions, the period effect RR showed consistent declines throughout the entire period. In the Southeast, RR decreased from 1.34 [CI 1.29; 1.40] to 0.79 [CI 0.77; 0.82] for men and from 1.66 [CI 1.49; 1.86] to 0.84 [CI 0.77; 0.93] for women, while in the South region, declined from approximately 1.24 [CI 1.02; 1.50] to 0.81 [CI 0.70; 0.95] for both genders. In the Northeast, a continuous increase was observed throughout the period, with RR reaching 1.84 [CI 1.73; 1.95] for men and 1.76 [CI 1.53; 2.03] for women by 2020. In the North and Mid-west, an increase in period RR was observed only among men (1.38 [CI 1.22; 1.56] and 1.24 [CI 1.12; 1.37] in 2015, respectively). Regarding the cohort effect, an increasing RR up to the 1955 cohorts, followed by a decline in subsequent cohorts, was observed in the Southeast and South for both genders and in the Mid-west among men. No significant cohort effect for LC was observed in the North and Northeast (Fig. 9, Tables S12–S16 [Supplementary Material]). A continuous decrease in RR over the period was observed among white men (from 1.16 [CI 1.12; 1.19] in 2000 to 0.79 [CI 0.77; 0.82] in 2020), while among browns, an increase of approximately 40% in RR was observed until 2010, followed by stabilization in both genders. Regarding the cohort effect, an increasing RR was observed up to the 1955 cohorts, followed by a decline in subsequent cohorts among men of all ethnicities. In women, this pattern was observed only among white women, with no improvement in mortality observed across cohorts for black and brown women (Figs. 10 and 11, Tables S17–S19 [Supplementary Material]).

**Table 1:** Mortality rate (per 100,000 persons) and relative risk (95% confidence interval) from laryngeal, oropharyngeal and oral cavity cancer by gender in Brazil, 1980–2023.

	Laryngeal cancer		Oropharyngeal cancer		Oral cavity cancer	
	Male	Female	Male	Female	Male	Female
	Rate (95%x CI)	Rate (95% CI)	Rate (95% CI)	Rate (95% CI)	Rate (95% CI)	Rate (95% CI)
**Age group**						
40	2.04 (1.96–2.12)	0.23 (0.20–0.25)	1.85 (1.76–1.94)	0.18 (0.16–0.21)	1.83 (1.75–1.92)	0.22 (0.20–0.25)
45	5.13 (4.98–5.30)	0.44 (0.40–0.49)	4.03 (3.87–4.18)	0.34 (0.30–0.38)	3.88 (3.74–4.03)	0.44 (0.40–0.48)
50	9.32 (9.07–9.59)	0.82 (0.75–0.89)	6.63 (6.40–6.87)	0.57 (0.51–0.63)	6.29 (6.07–6.52)	0.74 (0.68–0.80)
55	13.83 (13.46–14.21)	1.33 (1.23–1.44)	8.92 (8.61–9.24)	0.87 (0.79–0.96)	8.53 (8.24–8.84)	1.16 (1.07–1.25)
60	18.14 (17.66–18.64)	1.88 (1.74–2.03)	10.44 (10.07–10.82)	1.09 (0.99–1.19)	10.06 (9.72–10.42)	1.64 (1.52–1.76)
65	21.84 (21.24–22.45)	2.37 (2.20–2.56)	11.18 (10.76–11.62)	1.44 (1.31–1.58)	11.06 (10.66–11.47)	2.25 (2.09–2.42)
70	25.48 (24.72–26.26)	3.01 (2.77–3.27)	11.00 (10.53–11.49)	1.90 (1.72–2.11)	12.23 (11.75–12.74)	3.31 (3.07–3.58)
75	27.44 (26.58–28.34)	3.67 (3.37–4.00)	11.44 (10.91–11.99)	2.39 (2.16–2.65)	13.63 (13.06–14.22)	4.65 (4.30–5.02)
80	29.73 (28.79–30.70)	4.74 (4.37–5.15)	11.47 (10.94–12.02)	3.59 (3.26–3.95)	17.58 (16.88–18.31)	9.49 (8.83–10.2)

	RR (95% CI)	RR (95% CI)	RR (95% CI)	RR (95% CI)	RR (95% CI)	RR (95% CI)
**Period**						
1980	1.23 (1.19–1.26)	1.37 (1.26–1.49)	0.61 (0.58–0.64)	0.60 (0.53–0.69)	1.26 (1.21–1.31)	1.29 (1.20–1.40)
1985	1.17 (1.13–1.20)	1.26 (1.16–1.36)	0.75 (0.71–0.78)	0.89 (0.80–0.99)	1.18 (1.14–1.23)	1.09 (1.01–1.17)
1990	1.16 (1.13–1.19)	1.27 (1.18–1.37)	0.80 (0.77–0.83)	0.99 (0.90–1.09)	1.23 (1.18–1.27)	1.11 (1.03–1.19)
1995	1.18 (1.15–1.21)	1.32 (1.23–1.41)	0.99 (0.95–1.02)	1.05 (0.96–1.14)	1.08 (1.04–1.12)	1.09 (1.02–1.16)
2000	1.00 (Reference)	1.00 (Reference)	1.00 (Reference)	1.00 (Reference)	1.00 (Reference)	1.00 (Reference)
2005	1.06 (1.03–1.08)	1.10 (1.03–1.18)	1.02 (0.99–1.05)	1.13 (1.04–1.22)	1.02 (0.99–1.05)	1.14 (1.08–1.21)
2010	1.07 (1.05–1.10)	1.12 (1.05–1.19)	1.04 (1.01–1.07)	1.14 (1.06–1.23)	1.00 (0.97–1.04)	1.16 (1.10–1.23)
2015	1.03 (1.00–1.05)	1.08 (1.01–1.15)	1.10 (1.07–1.14)	1.17 (1.09–1.27)	1.05 (1.01–1.08)	1.12 (1.06–1.19)
2020	0.98 (0.96–1.00)	1.01 (0.94–1.07)	1.04 (1.01–1.08)	1.12 (1.04–1.21)	1.02 (0.98–1.05)	1.08 (1.02–1.15)
**Birth cohort**						
1900	0.70 (0.63–0.78)	0.63 (0.51–0.78)	0.89 (0.73–1.09)	1.10 (0.82–1.47)	0.96 (0.86–1.08)	0.80 (0.68–0.93)
1905	0.77 (0.72–0.81)	0.82 (0.72–0.94)	0.90 (0.81–1.01)	0.99 (0.83–1.19)	0.79 (0.73–0.86)	0.98 (0.88–1.10)
1910	0.77 (0.74–0.80)	0.85 (0.77–0.95)	0.83 (0.77–0.90)	1.05 (0.92–1.21)	0.81 (0.76–0.86)	0.97 (0.89–1.07)
1915	0.74 (0.72–0.77)	0.83 (0.76–0.91)	0.76 (0.71–0.81)	1.04 (0.93–1.17)	0.76 (0.72–0.80)	1.11 (1.03–1.21)
1920	0.77 (0.74–0.79)	0.80 (0.74–0.87)	0.74 (0.70–0.78)	0.85 (0.77–0.95)	0.75 (0.72–0.78)	0.97 (0.90–1.05)
1925	0.87 (0.84–0.89)	0.84 (0.78–0.90)	0.85 (0.81–0.88)	0.92 (0.83–1.01)	0.83 (0.80–0.86)	1.05 (0.98–1.12)
1930	0.93 (0.90–0.95)	0.92 (0.86–0.99)	0.84 (0.81–0.87)	0.94 (0.86–1.03)	0.90 (0.87–0.93)	1.04 (0.97–1.11)
1935	0.93 (0.91–0.95)	0.91 (0.85–0.97)	0.85 (0.82–0.88)	0.95 (0.88–1.03)	0.90 (0.87–0.93)	1.06 (1.00–1.13)
1940	0.93 (0.91–0.95)	0.96 (0.90–1.02)	0.86 (0.83–0.89)	0.93 (0.86–1.01)	0.90 (0.87–0.93)	1.05 (0.99–1.12)
1945	0.92 (0.90–0.94)	0.93 (0.86–0.99)	0.86 (0.84–0.89)	0.89 (0.82–0.97)	0.92 (0.90–0.95)	0.99 (0.93–1.06)
1950	0.94 (0.92–0.97)	0.93 (0.87–0.99)	0.94 (0.91–0.96)	0.95 (0.87–1.03)	0.96 (0.93–0.99)	0.95 (0.88–1.02)
1955	1.00 (Reference)	1.00 (Reference)	1.00 (Reference)	1.00 (Reference)	1.00 (Reference)	1.00 (Reference)
1960	0.94 (0.91–0.96)	0.98 (0.91–1.05)	0.94 (0.91–0.97)	1.06 (0.97–1.16)	0.96 (0.93–0.99)	1.02 (0.94–1.10)
1965	0.8 (0.78–0.83)	0.86 (0.79–0.94)	0.84 (0.81–0.87)	1.02 (0.93–1.13)	0.83 (0.80–0.86)	1.03 (0.94–1.12)
1970	0.70 (0.67–0.72)	0.76 (0.68–0.86)	0.73 (0.69–0.76)	0.92 (0.81–1.04)	0.73 (0.69–0.76)	0.95 (0.85–1.06)
1975	0.61 (0.57–0.64)	0.62 (0.52–0.74)	0.63 (0.59–0.68)	0.93 (0.78–1.10)	0.57 (0.53–0.61)	1.01 (0.88–1.17)
1980	0.47 (0.42–0.53)	0.47 (0.33–0.65)	0.56 (0.50–0.62)	0.88 (0.66–1.16)	0.54 (0.48–0.60)	1.20 (0.96–1.50)

### Oropharyngeal cancer

#### Trends

An increasing mortality trend was observed among men (AAPC of 1.53 [CI 1.29; 1.77]) and women (AAPC of 1.04 [CI 0.77; 1.31]) in Brazil as a whole. For all Brazilian regions, an increasing mortality trend for both sexes was observerd. The highest trends were found in the Northeast (men: AAPC 4.36 [CI 4.01; 4.66], women: AAPC 3.59 [CI 3.18; 3.99]) and North (men: AAPC 3.60 [CI 2.67; 4.55], women: AAPC 1.70 [CI 0.82; 2.59]) (Fig. 1, Table S6 [Supplementary Material]). Regarding ethnicity, a increasing trend was observed among browns (men AAPC −2.38; women −1.87), while an decreasing mortality trend was only in white males (AAPC −0.61 [CI −0.98; −0.24]) (Fig. 2, Table S7 [Supplementary Material]).

In Brazil, men showed a significant upward trend in mortality starting from age 45, with a peak in the 65–69 age group (AAPC 2.15 [CI 1.83; 2.47]). In women, this increasing trend was observed in all age groups, peaking in the 50–54 age group (AAPC 2.26 [CI 1.25; 3.28]) (Fig. 3, Table S8 [Supplementary Material]). An increasing trend in mortality was observed across all age groups for both genders in the Northeast (AAPC 4.87 [CI 4.26; 5.47] in men and 4.11 [CI 2.99; 5.23] in women at age 65) and among men in the Midwest (AAPC 5.02 [CI 3.73; 6.31] at age 65). In the South and Southeast, this increase was observed primarily among individuals aged over 50 years (AAPC 1.62 [CI 1.14; 2.10] in men from the South and 1.48 [CI 1.13; 1.83] in men from the Southeast at age 65). A declining trend was observed among women under 50 years in the North (AAPC −2.48 [CI −3.37; −1.58] for those aged 50–54) (Figs. 4 and 5, Table S9 [Supplementary Material]). For ethnicity, browns showed an increasing trend, particularly among males and older age groups (AAPC 3.02 [CI 2.31; 3.74] for those aged 65–69 years), while a decreasing trend were observerd among whites men, more pronounced in younger individuals (AAPC −4.82 [CI −5.50; −4.14]) (Figs. 6 and 7, Table S10 [Supplementary Material]).

#### Age-period-cohort

**Fig. 12:**
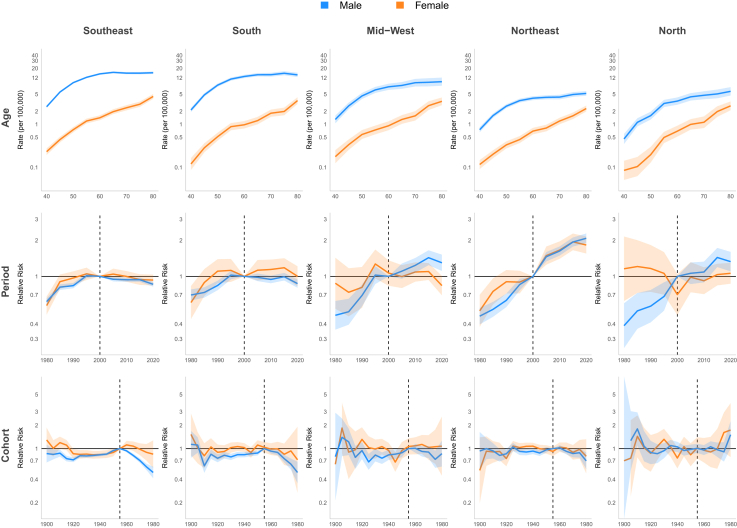
Age, period, and cohort effects on mortality from oropharyngeal cancer by gender in Brazilian regions, 1980–2023.

For Brazil model, a rising RR trend in period effect was observed in women over time. The period before 2000 displayed lower risk (1980 RR: 0.60 [CI 0.53; 0.69]), whereas after 2000, the RR increased progressively, RR 1.17 [CI 1.09; 1.27] in 2015. For men, this upward trend continued until 2000 (1980 RR: 0.61 [CI 0.58; 0.64]), but after 2000 RR showed stabilization. Birth cohort effect showed, among men, that the highest RR occurred in the 1955 cohort, with preceding and subsequent cohorts demonstrating lower risks. The 1980 cohort showed the series' minimum risk (RR 0.56 [CI 0.50; 0.62]). Women maintained stable RR across cohorts (Fig. 8, Table 1). The period effect in the models for Brazilian regions revealed a consistent increase throughout the entire series in RR for men in the Midwest (2015 RR: 1.43 [CI 1.24; 1.65]), Northeast (2015 RR: 1.94 [CI 1.77; 2.25]), and North (2015 RR: 1.44 [CI 1.20; 1.73]). For women, there was a progressive increase in RR in the Northeast (2015 RR: 1.94 [CI 1.66; 2.28]), while other regions showed stabilization after 2000. An increasing RR up to the 1955 birth cohorts, followed by a decline in subsequent cohorts, was observed in the Southeast, South, and Mid-west for men. No significant cohort effect was observed in women in any region except the Southeast (Fig. 12, Tables S12–S16 [Supplementary Material]). A decrease in RR was observed among white men until 2010 (from 1.16 [CI 1.12; 1.19] in 2000 to 1.00 [CI 0.96; 1.05] in 2010) followed by stabilization, while an increasing was seen among brown men over the period (from 0.76 [CI 0.71; 0.82] in 2000 to 1.21 [CI 1.15; 1.28] in 2020). Regarding the cohort effect, an increasing RR was observed up to the 1955 cohorts, followed by a decline in subsequent cohorts among men of all ethnicities. In women, no cohort or period effect was observed (Figs. 10 and 11, Tables S17–S19 [Supplementary Material]).

### Oral cavity cancer

#### Trends

The mortality trend from oral cavity cancer among Brazilian men showed a decline; AAPC of −0.30 [CI −0.46; −0.15]. For Brazilian regions, increasing mortality trends were observed for both genders in the Northeast (men: AAPC 2.42 [CI 2.10; 2.74]; women: AAPC 1.73 [CI 1.31; 2.16]) and for men in the Midwest (AAPC 0.57 [CI 0.21; 0.92]) and North (AAPC 1.12 [CI 0.66; 1.59]). A declining trend was observed in the Southeast for both genders (men: AAPC −1.15 [CI −1.35; −0.95]; women: AAPC −0.93 [CI −1.19; −0.66]) and among men in the South (AAPC −0.97 [CI −1.18; −0.76]) (Fig. 1, Table S6 [Supplementary Material]). Regarding ethnicity, a decreasing trend was observed only among White men white AAPC −1.01 [CI −1.32; −0.71], but increasing in Browns (AAPC 1.60 [CI 1.20; 2.09]), whereas this trend was not demonstrated among whites women (AAPC 0.06 [CI −0.33; 0.46]; p = 0.75) (Fig. 2, Table S7 [Supplementary Material]).

Men under 60 years old had a declining mortality trend, most pronounced reduction observed in 40–44 age group (AAPC −1.66 [CI −2.02; −1.29]) (Fig. 3, Table S8 [Supplementary Material]). Men in the Southeast showed a declining mortality trend across all age groups, with an AAPC of −2.46 [CI −2.73; −2.18] among those aged 45–49 years. In contrast, an increasing mortality trend was observed in both genders in the Northeast region, reaching an AAPC of 3.37 [CI 2.87; 3.89] among men aged 65 years (Figs. 4 and 5, Table S9 [Supplementary Material]). Browns male showed an increasing trend in invidiuals over 55 years-old (AAPC 3.02 [CI 1.85; 4.17] for those aged 70–74 years), while a decreasing trend were observerd among whites and black younger men (AAPC -3.84 [CI −4.58; −3.10] for white and −3.71 [CI −5.34; −2.05] for black aged 45–49 years) (Figs. 6 and 7, Table S10 [Supplementary Material]).

#### Age-period-cohort

**Fig. 13:**
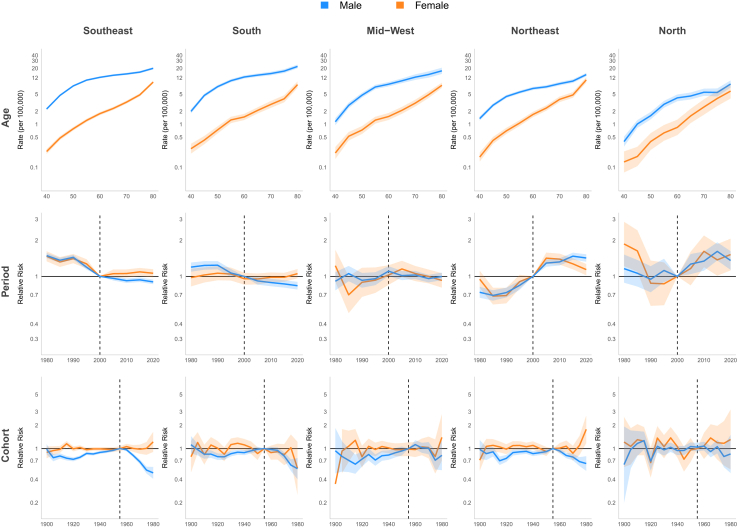
Age, period, and cohort effects on mortality from oral cavity cancer by gender in Brazilian regions, 1980–2023.

The period effect showed declining relative risk (RR) between 1980 and 2000 in both genders (1980–RR: 1.26 [CI 1.21; 1.31] for men, 1.29 [CI 1.20; 1.40] for women to 1.00 in 2000 [reference]). After 2000, male RR remained stable RR while women experienced up to a 16% RR increase among 2005 to 2019. OCC mortality risk among men increased progressively in cohorts born up to 1955, with gradual decline post-1955. The lowest series RR occurred in the final cohort (1980; RR 0.54 [CI 0.48; 0.60]). Women exhibited stable RR throughout, though an upward trend appeared from the 1980 cohort (RR: 1.20 [CI 0.96; 1.50]) (Fig. 8, Table 1). In the Southeast and South regions, the period effect for mortality declined throughout the entire period for men (∼40% reduction in the Southeast and 30% in the South). However, among women in the Southeast, the decline lasted only until 2000, followed by an increase (2015 RR: 1.09 [CI 1.01; 1.18]). In the Northeast, a continuous increase was observed throughout the study period, with RR reaching 1.47 for men in 2015 [CI 1.38; 1.58] and 1.39 [CI 1.25; 1.55] for women in 2010. No significant cohort effect for OCC was observed among women in any Brazilian region. However an increasing RR up to the 1955 birth cohorts, followed by a decline in subsequent cohorts, was observed for men in all regions except the North (Fig. 13, Tables S12–S16 [Supplementary Material]). A continuous increase in RR over the period was observed among brown men (from 0.82 [CI 0.77; 0.88] in 2000 to 1.10 [CI 1.05; 1.16] in 2020), while among whites, an decrease of approximately 21.4% in RR was observed from 2000 to 2020. Regarding the cohort effect, an increasing RR was observed up to the 1955 cohorts, followed by a decline in subsequent cohorts among men of all ethnicities. In women, no cohort and period effects were observed (Figs. 10 and 11, Tables S16–S19 [Supplementary Material]).

## Discussion

Our study is the first to analyzed mortality trends in HNC over 44 years in Brazilian regions and Brazil as a whole by ethnicity. The results demonstrated that men continue to have higher mortality rates across all subsites, with LC showing the highest. Regarding women with HNC, a significant increase in mortality risk was observed in the recent years, for OCC and OPC, mainly among young women in Brazil. Regional disparities were observed, whereas the Northeast region exhibited increasing mortality trends for LC, OPC, and OCC among both genders, the South and Southeast regions demonstrated declining mortality rates for LC and OCC. Our analysis of gender and ethnicity revealed that white Brazilian males were the group showing significant decline in mortality for LC, OPC and OCC, whereas browns of both genders demonstrated increasing mortality rates and trends for these malignancies.

In Brazil, mortality rates for LC, OPC and OCC remain high, mainly among older individuals. LC in men was the most lethal, with rates reaching 25 per 100,000 individuals among 75-year-old men. In contrast, developed countries, such as the USA, show significantly lower and decreasing mortality rates for LC, accompanied by a decline in incidence, greater among men than among women.^9^ In our study, for women, oral cavity cancer had the highest mortality, reaching 5.3 per 100,000 among 75-year-old women. Zhu Z et al. reported similar mortality rates for women but higher for men (approximately 4.8 per 100,000 for women and 31.6 per 100,000 for men) in China, which could reflect healthcare policies and environmental factors,^10^ However, decreasing mortality were observed in some European countries, for example Denmark (AAPC −1.63 for women and −2.81 for men) and Norway (AAPC −1.13 for women and −2.06 for men),^11^ may attributed to early diagnosis. Different scenario is found in Brazil, a study conducted with national data demonstrated that most oral cavity tumors are diagnosed at an advanced stage.^12^ In some European countries,^11^ although incidence rates have been progressively increasing, mortality does not follow this pattern and has shown a decline, which could be attributed to the rise in HPV-associated OPC and its better prognosis. In our study, the oropharynx shows the lowest mortality rates for both men and women but displays an increasing mortality trend, in line with the rising incidence of this cancer in Brazil.^13^ These differences between Brazil and high-income countries may reflect a combination of factors, including later stage at diagnosis,^12^ limited access to oncological care, pronounced regional disparities in healthcare access and in the distribution of healthcare professionals and resources.^14^^,^^15^

Reduction in the relative risk of death was observed among men born after 1955 across all HNC subsites. This cohort effect may reflect the impact of Brazil's recognized intensive tobacco control efforts,^16^ considering that individuals born in 1955 were between 30 and 40 years old, at an age susceptible to habit change. Brazilian government, made other actions as the expansion of health warnings on cigarette packs^17^ and the creation of laws regulating tobacco taxation between 2009 and 2011. The combination of these initiatives contributed to a reduction in more than 40% of tobacco consumption in Brazil.^18^ This may have impacted mortality trends, considering that the peak reduction in mortality was observed among individuals born between 1980, who were aged 30 years in 2010. The decline in tobacco use has influenced the incidence^19^ and mortality of HPV-unrelated HNCs, largely due to tobacco's significant contribution to their carcinogenesis.^20^ As observed in this study, LC, the subsite more related to tobacco, showed the greatest improvements in mortality. Public policies aimed at reducing alcohol consumption started later compared to those targeting tobacco^21^^,^^22^ and presented less effectiveness compared to tobacco.^16^^,^^18^^,^^23^ Thus, the benefits of these government actions may not yet reflect the effects on the birth cohorts observed in this study. The difficulty in implementing public policies aimed at controlling alcohol consumption in the country, mainly among younger individuals,^24^ can be explained by the fact that alcohol consumption is still widely accepted in cultural and social practices.

This study highlighted different mortality patterns by subsite. For LC, a reduction in relative risk was observed up until the 2000s, followed by a slight increase and stabilization. Although tobacco control campaigns positively impacted mortality, research suggests that recent changes in the therapeutic approach to LC, with a preference for more conservative treatments aiming at organ function preservation,^9^^,^^25^ may have affected the prognosis of these patients, resulting in a stabilization of outcomes. Regarding OPC, an increase in mortality relative risk and trends was observed among both genders and Brazilian regions throughout the entire period. Studies reported that this finding could be explain for the burden of HPV-associated OPC incidence worldwide.^19^ Although it has already been reported that HPV-related OPC presents better outcomes,^26^ this rise in incidence^13^ may have impacted the mortality from this type of cancer in the country, mainly in women aged 40–55 years.

In Brazil, differences mortality patterns between genders were observed, mainly for OPC and OCC. While mortality for OCC decreased until 2000 for all individuals, gender differences were observed in more recent periods, with a significant increase in the mortality trend for women, while the relative risk remained stable and the AAPC declined for men. This finding aligns with a Brazilian study, which identified a more pronounced upward trend for women than for men,^27^ also found in a study that analyzed global mortality.^11^ To consider in the increasing risk of OPC and OCC in women involves the sociocultural aspects of the Brazilian population. Significant changes in social and gender norms have occurred in recent decades worldwide, with the advancement of the feminist movement for gender equality and women autonomy.^28^ As women began to occupy positions traditionally held by men, they also experienced shifts in lifestyle behaviors, including consumption of alcohol and tobacco, as demonstrated by national^18^ and international data.^11^ In Brazil, data from a survey conducted in all state capitals showed that the number of women who consumed more than four alcoholic drinks in the past 30 days increased by 94% between 2006 and 2023, while the increase among men was less than 10%.^18^ This study highlights the context of cancer in relation to changes in gender norms. LC, OPC and OCC has historically been associated with men. As a result, healthcare professionals and public policies may have focused more on this specific population, which has recently led to negative outcomes for women in terms of mortality.

Strong regional differences can be observed in the mortality patterns of LC, OCC, and OPC in this study. The Northeast region, which is economically less developed and historically disadvantaged, showed an increase in mortality for all HNC subsites across both genders. In contrast, the South and Southeast, characterized by higher levels of economic development, presented a decline in mortality from LC and OCC. One of the reason for these disparities may be the unequal and limiting access to access to adequate cancer care in Brazilian population and the lack of available resources for treatment in less developed regions, with the North and Northeast regions facing the greatest challenges.^14^^,^^29^ Furthermore, these regions have the highest proportion of individuals who do not attend dental appointments regularly, despite dentists playing an important role in early diagnosis.^30^ Conversely, regions with higher socioeconomic development, such as South and Southeast, tend to have more health professionals and infrastructure, resulting in fewer barriers to the diagnosis and treatment of chronic diseases.^29^

Other studies have also demonstrated regional disparities in cancer mortality. Analyses focusing on breast, gastric and oral cavity and lip cancer identified a similar increasing trend in mortality in the Northeast, North, and Mid-West regions.^27^ As demonstrated in this study, regions with limited economic resources and healthcare access, despite still having lower overall mortality rates, are showing a steady and concerning increase in deaths from OCC, OPC, and LC. This upward trend may increasingly contribute to a worsening of national HNC mortality rates. However, this result should still be interpreted with caution, as the increasing mortality trends for OPC, OCC, and LC observed in the Northeast region may suggest improvements in the mortality information system,^31^ which could have led to an underestimation of the number of deaths in earlier periods.

Social and cultural environments, including lifestyle and increased exposure to risk factors, may also contribute to the increase in cancer mortality, especially in a country of large territorial extension.^27^^,^^32^ According to Vigitel data from 2006 to 2023, in the most recent year, capitals in the North, Northeast, and Mid-West regions showed an increase in the percentage of alcohol consumers (Boa Vista–North; Salvador and Maceió—Northeast; and Campo Grande and Cuiabá—Mid-West) and smokers (Belém—North; Cuiabá—Mid-West; and João Pessoa and Maceió—Northeast), while São Paulo–Southeast, continues to show a steady decline in smoking prevalence.^18^ Perea et al., 2018 also demonstrated a reduction in oral cancer mortality trends between 2002 and 2013 in the Southeast region, which was attributed to their intensive anti-smoking policies.^33^

Disparities in HNC mortality were observed by ethnicity in our study. White men showed a decreasing mortality trend across all subsites, more pronounced for LC. A study using data from the Surveillance, Epidemiology, and End Results (SEER) program found that black individuals, across all socioeconomic categories, have worse outcomes when compared to whites.^34^ The persistence of significant racial disparities in HNC outcomes, even when controlled for income, socioeconomic index, employment status, insurance, and education, was also reported in a meta-analysis.^35^ In our study, increased mortality was mainly observed among brown individuals of both genders and black men. However, a Brazilian study conducted in São Paulo between 2001 and 2017 demonstrated higher mortality rates for black individuals, followed by whites, and then browns, for cancer subsites such as colorectal, stomach, and lung.^36^ These differences may be explained by differences in healthcare access in this region and Brazil.

Some limitations of our study include the lack of information on factors associated with mortality, such as smoking, alcohol consumption. Although we were able to analyze mortality trends by ethnicity, the high proportion of missing data in records before 2000, between 1996 and 1999 accounted for 51% of all missing cases, limited the assessment for a more comprehensive time period analysis, potentially obscuring temporal trends. Additionally, this analysis was conducted using two data sources, the SIM and the Census by IBGE, which determine race/ethnicity through different methods. While the IBGE uses self-identification, in the SIM race/ethnicity is determined by heteroclassification.^3^ Furthermore, it is important to highlight that variations in data quality across regions may have influenced the observed mortality trends, potentially affecting their magnitude. Subnational disparities in the quality of death records are still concerning, with much poorer performance observed in the North and Northeast regions, especially in municipalities with lower socioeconomic index and limited healthcare infrastructure.^31^^,^^37^ Although we used established redistribution methodologies to minimize the impact of ill-defined causes of death, some degree of misclassification and underreporting may persist. Improvements in death certification over time could also have contributed to apparent increases in mortality rates in earlier periods, especially in less-developed regions.

Our findings demonstrated that mortality from oral cavity, oropharyngeal, and laryngeal cancer in Brazil has decreased among men, mainly due to the positive impact of tobacco control policies. However, the increase in mortality among women highlights that these policies have not effectively addressed this population. Shifts in lifestyle behaviors and social changes among women may be associated with this rising mortality trend. Gender, ethnicity and geographic location are associated with HNC mortality in Brazil, white males showed the most substantial improvements in mortality rates, while browns individuals exhibited concerning increases across all cancer subsites. The North and Northeast regions, have experienced a significant increase in mortality from oral cavity, oropharyngeal, and laryngeal cancers.

## Contributors

MA contributed to conceptualisation, data curation, formal analysis, investigation, visualisation, and wrote the original draft and the manuscript review and editing. MPC contributed to conceptualisation, funding acquisition, investigation, project administration, resources, supervision, validation, visualisation, and wrote the manuscript review and editing. MRDOL contributed to data curation, formal analysis, investigation, supervision, validation, visualisation, and wrote the manuscript review and editing. MA and MRDOL directly accessed and verified the data.

All authors had full access to all the raw data in the study and accept responsibility to submit for publication.

## Data sharing statement

This publication contains results from a secondary analysis of publicly available, de-identified population-based data. These data are accessible through the links provided in the Methods section.

## AI use statement

The authors declare that generative artificial intelligence (AI) tool was used in the creation of this manuscript. ChatGPT (GPT-5 model, OpenAI; https://chat.openai.com) was employed to review grammar and spelling. The authors reviewed and edited all AI-assisted text to ensure accuracy and integrity, and take full responsibility for the final content of this publication. No generative AI tools were used in data analysis, interpretation, or to draw insights from the results.

## Declaration of interests

We declare no competing interests.

## Appendix A Supplementary data

Supplementary Figures and Tables

Translated Summary
