# Supplementary material for: Head and neck cancer mortality by gender, region and ethnicity: a population-based study in Brazil
Source: Lancet Reg Health Am. 2025 Nov 29;53:101306. doi: 10.1016/j.lana.2025.101306 (PMC12703860; doi:10.1016/j.lana.2025.101306)
Supplement: Supplementary Figures and Tables [file mmc1.docx]

**Supplementary File**

**Figure 1.** Brazilian population estimates by ethnicity (white, brown and black) from 2000 to 2023 based on Census 2000, 2010 and 2022.

**
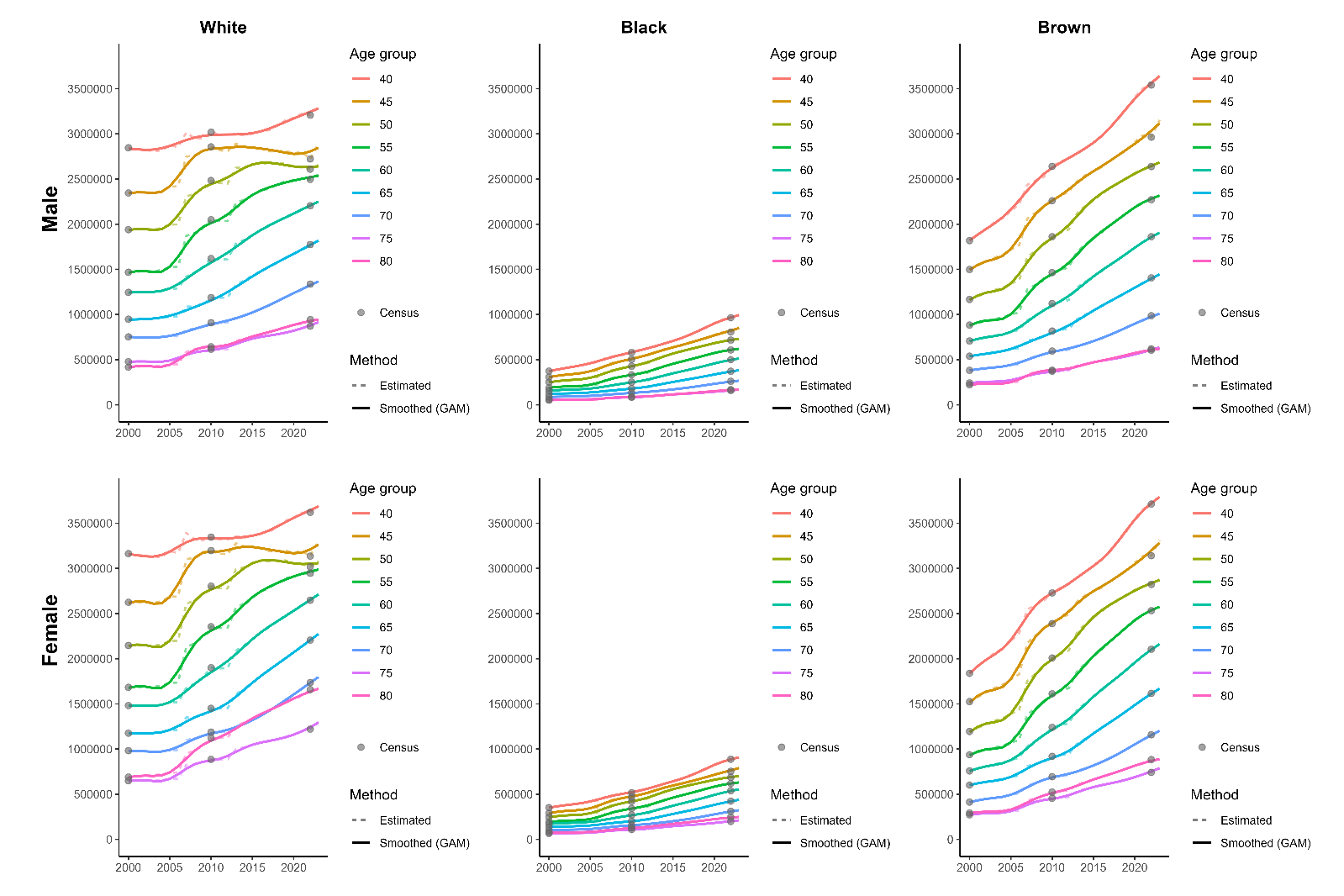
**

**Table 1.** Proportion of death records with unknown age by subsite in Brazil, 1980–2023.

|  | **Larynx** | | |  | **Oropharynx** | | |  |  | **Oral cavity** | | |
| --- | --- | --- | --- | --- | --- | --- | --- | --- | --- | --- | --- | --- |
| **Year** | **Age 0-80+ years** | **Unknown age** | **Unknown age (%)** |  | **Age 0-80+ years** | **Unknown age** | **Unknown age (%)** | |  | **Age 0-80+ years** | **Unknown age** | **Unknown age (%)** |
| **1980** | 1496 | 2 | 0·13 |  | 411 | 0 | 0·00 |  |  | 1082 | 3 | 0·28 |
| **1981** | 1561 | 3 | 0·19 |  | 468 | 2 | 0·43 |  |  | 1069 | 5 | 0·47 |
| **1982** | 1575 | 8 | 0·51 |  | 523 | 2 | 0·38 |  |  | 1163 | 7 | 0·60 |
| **1983** | 1592 | 2 | 0·13 |  | 480 | 0 | 0·00 |  |  | 1133 | 5 | 0·44 |
| **1984** | 1687 | 7 | 0·41 |  | 537 | 0 | 0·00 |  |  | 1135 | 3 | 0·26 |
| **1985** | 1747 | 3 | 0·17 |  | 595 | 3 | 0·50 |  |  | 1150 | 9 | 0·78 |
| **1986** | 1708 | 9 | 0·53 |  | 662 | 3 | 0·45 |  |  | 1178 | 6 | 0·51 |
| **1987** | 1837 | 11 | 0·60 |  | 711 | 3 | 0·42 |  |  | 1210 | 10 | 0·83 |
| **1988** | 1809 | 6 | 0·33 |  | 790 | 4 | 0·51 |  |  | 1262 | 6 | 0·48 |
| **1989** | 1925 | 8 | 0·42 |  | 765 | 5 | 0·65 |  |  | 1294 | 9 | 0·70 |
| **1990** | 1891 | 8 | 0·42 |  | 798 | 2 | 0·25 |  |  | 1295 | 2 | 0·15 |
| **1991** | 1991 | 7 | 0·35 |  | 808 | 7 | 0·87 |  |  | 1423 | 9 | 0·63 |
| **1992** | 2086 | 20 | 0·96 |  | 862 | 5 | 0·58 |  |  | 1491 | 10 | 0·67 |
| **1993** | 2224 | 4 | 0·18 |  | 938 | 4 | 0·43 |  |  | 1457 | 5 | 0·34 |
| **1994** | 2234 | 7 | 0·31 |  | 925 | 7 | 0·76 |  |  | 1624 | 4 | 0·25 |
| **1995** | 2397 | 8 | 0·33 |  | 901 | 8 | 0·89 |  |  | 1561 | 7 | 0·45 |
| **1996** | 2336 | 10 | 0·43 |  | 1218 | 7 | 0·57 |  |  | 1423 | 5 | 0·35 |
| **1997** | 2562 | 8 | 0·31 |  | 1227 | 4 | 0·33 |  |  | 1484 | 11 | 0·74 |
| **1998** | 2525 | 5 | 0·20 |  | 1297 | 5 | 0·39 |  |  | 1479 | 4 | 0·27 |
| **1999** | 2575 | 5 | 0·19 |  | 1382 | 0 | 0·00 |  |  | 1690 | 6 | 0·36 |
| **2000** | 2427 | 2 | 0·08 |  | 1490 | 2 | 0·13 |  |  | 1669 | 3 | 0·18 |
| **2001** | 2664 | 4 | 0·15 |  | 1407 | 2 | 0·14 |  |  | 1791 | 3 | 0·17 |
| **2002** | 2798 | 4 | 0·14 |  | 1565 | 0 | 0·00 |  |  | 1858 | 2 | 0·11 |
| **2003** | 2733 | 5 | 0·18 |  | 1642 | 3 | 0·18 |  |  | 1943 | 4 | 0·21 |
| **2004** | 2951 | 3 | 0·10 |  | 1715 | 1 | 0·06 |  |  | 2005 | 1 | 0·05 |
| **2005** | 3157 | 3 | 0·10 |  | 1855 | 2 | 0·11 |  |  | 2118 | 0 | 0·00 |
| **2006** | 3305 | 0 | 0·00 |  | 1765 | 0 | 0·00 |  |  | 2206 | 2 | 0·09 |
| **2007** | 3402 | 2 | 0·06 |  | 1869 | 1 | 0·05 |  |  | 2341 | 1 | 0·04 |
| **2008** | 3623 | 2 | 0·06 |  | 1991 | 1 | 0·05 |  |  | 2389 | 1 | 0·04 |
| **2009** | 3490 | 4 | 0·11 |  | 2133 | 1 | 0·05 |  |  | 2450 | 2 | 0·08 |
| **2010** | 3618 | 2 | 0·06 |  | 2171 | 0 | 0·00 |  |  | 2451 | 1 | 0·04 |
| **2011** | 3891 | 3 | 0·08 |  | 2227 | 0 | 0·00 |  |  | 2566 | 0 | 0·00 |
| **2012** | 4339 | 2 | 0·05 |  | 2269 | 0 | 0·00 |  |  | 2632 | 1 | 0·04 |
| **2013** | 4141 | 1 | 0·02 |  | 2287 | 1 | 0·04 |  |  | 2804 | 1 | 0·04 |
| **2014** | 4142 | 0 | 0·00 |  | 2421 | 0 | 0·00 |  |  | 2842 | 2 | 0·07 |
| **2015** | 4384 | 0 | 0·00 |  | 2530 | 0 | 0·00 |  |  | 3009 | 1 | 0·03 |
| **2016** | 4382 | 1 | 0·02 |  | 2739 | 2 | 0·07 |  |  | 2999 | 3 | 0·10 |
| **2017** | 4501 | 0 | 0·00 |  | 2850 | 3 | 0·11 |  |  | 3071 | 1 | 0·03 |
| **2018** | 4455 | 1 | 0·02 |  | 2798 | 1 | 0·04 |  |  | 3250 | 0 | 0·00 |
| **2019** | 4532 | 2 | 0·04 |  | 2919 | 1 | 0·03 |  |  | 3279 | 1 | 0·03 |
| **2020** | 4478 | 0 | 0·00 |  | 2675 | 0 | 0·00 |  |  | 3085 | 0 | 0·00 |
| **2021** | 4562 | 1 | 0·02 |  | 2678 | 1 | 0·04 |  |  | 3253 | 1 | 0·03 |
| **2022** | 4612 | 1 | 0·02 |  | 2684 | 2 | 0·07 |  |  | 3444 | 1 | 0·03 |
| **2023** | 4689 | 0 | 0·00 |  | 3287 | 0 | 0·00 |  |  | 3358 | 1 | 0·03 |

**Table 2.** Proportion of missing values in the ethnicity variable in the Brazilian Mortality Information System database for laryngeal, oral cavity, and oropharyngeal cancers, 1996-2023.

| **Year** | **Total deaths** | **Missing values (Ethnicity)** | **Missing (%)** |
| --- | --- | --- | --- |
| 1996 | 4,930 | 4,719 | 95·7 |
| 1997 | 5,240 | 2,688 | 51·3 |
| 1998 | 5,275 | 1,350 | 25·6 |
| 1999 | 5,580 | 985 | 17·6 |
| 2000 | 5,550 | 569 | 10·2 |
| 2001 | 5,827 | 541 | 9·3 |
| 2002 | 6,177 | 497 | 8·0 |
| 2003 | 6,272 | 398 | 6·3 |
| 2004 | 6,630 | 380 | 5·7 |
| 2005 | 7,092 | 395 | 5·6 |
| 2006 | 7,239 | 486 | 6·7 |
| 2007 | 7,558 | 508 | 6·7 |
| 2008 | 7,955 | 507 | 6·4 |
| 2009 | 8,028 | 532 | 6·7 |
| 2010 | 8,192 | 436 | 5·3 |
| 2011 | 8,626 | 449 | 5·2 |
| 2012 | 9,179 | 392 | 4·3 |
| 2013 | 9,163 | 401 | 4·4 |
| 2014 | 9,327 | 388 | 4·2 |
| 2015 | 9,859 | 366 | 3·7 |
| 2016 | 10,042 | 352 | 3·5 |
| 2017 | 10,341 | 279 | 2·7 |
| 2018 | 10,430 | 282 | 2·7 |
| 2019 | 10,662 | 263 | 2·5 |
| 2020 | 10,178 | 270 | 2·6 |
| 2021 | 10,433 | 250 | 2·4 |
| 2022 | 10,663 | 183 | 1·7 |
| 2023 | 11,262 | 190 | 1·7 |

**Table 3.** Number of deaths age-specific mortality rates (ASpMR), and age-standardized mortality rates (ASMR) (per 100,000 persons) for laryngeal, oropharyngeal and oral cavity cancers in Brazil, 1980–2023.

|  | **Laryngeal Cancer** | | | |  | **Oropharyngeal Cancer** | | | |  | **Oral Cavity Cancer** | | | |
| --- | --- | --- | --- | --- | --- | --- | --- | --- | --- | --- | --- | --- | --- | --- |
|  | **Male** | | **Female** | |  | **Male** | | **Female** | |  | **Male** | | **Female** | |
| **Age Group** | n | ASpMR | n | ASpMR |  | n | ASpMR | n | ASpMR |  | n | ASpMR | n | ASpMR |
| 40 | 4,005 | 1·73 | 509 | 0·21 |  | 3,230 | 1·40 | 443 | 0·18 |  | 3,624 | 1·57 | 616 | 0·25 |
| 45 | 9,188 | 4·65 | 924 | 0·44 |  | 6,445 | 3·26 | 729 | 0·35 |  | 6,925 | 3·51 | 1,041 | 0·49 |
| 50 | 15,080 | 8·89 | 1,542 | 0·84 |  | 9,581 | 5·65 | 1,076 | 0·59 |  | 10,181 | 6·00 | 1,507 | 0·83 |
| 55 | 18,993 | 13·61 | 2,159 | 1·40 |  | 10,981 | 7·87 | 1,399 | 0·91 |  | 11,653 | 8·35 | 2,014 | 1·31 |
| 60 | 20,173 | 18·02 | 2,495 | 1·98 |  | 10,357 | 9·25 | 1,402 | 1·11 |  | 11,070 | 9·89 | 2,319 | 1·84 |
| 65 | 18,277 | 21·30 | 2,441 | 2·46 |  | 8,295 | 9·66 | 1,429 | 1·44 |  | 9,124 | 10·63 | 2,523 | 2·54 |
| 70 | 14,646 | 24·17 | 2,240 | 3·04 |  | 5,592 | 9·23 | 1,391 | 1·89 |  | 6,930 | 11·44 | 2,767 | 3·75 |
| 75 | 10,240 | 25·52 | 1,935 | 3·67 |  | 3,786 | 9·43 | 1,258 | 2·38 |  | 4,993 | 12·44 | 2,815 | 5·34 |
| 80 | 10,377 | 27·07 | 2,752 | 4·60 |  | 3,650 | 9·52 | 2,197 | 3·67 |  | 6,066 | 15·82 | 6,497 | 10·86 |
| **Total / ASMR** | 120,979 | 16·11 | 16,997 | 2·07 |  | 61,917 | 7·25 | 11,324 | 1·39 |  | 70,566 | 8·85 | 22,099 | 3·02 |

**Table 4.** Number of deaths age-specific mortality rates (ASpMR), and age-standardized mortality rates (ASMR) (per 100,000 persons) for laryngeal, oropharyngeal and oral cavity cancers in Brazilian regions, 1980–2023.

|  | **Southeast** | | | | | | | | | | | | | |
| --- | --- | --- | --- | --- | --- | --- | --- | --- | --- | --- | --- | --- | --- | --- |
|  | **Laryngeal Cancer** | | | |  | **Oropharyngeal Cancer** | | | |  | **Oral Cavity Cancer** | | | |
|  | **Male** | | **Female** | |  | **Male** | | **Female** | |  | **Male** | | **Female** | |
| **Age Group** | n | ASpMR | n | ASpMR |  | n | ASpMR | n | ASpMR |  | n | ASpMR | n | ASpMR |
| 40 | 2,240 | 2·15 | 259 | 0·23 |  | 1,868 | 1·79 | 235 | 0·21 |  | 2,043 | 1·96 | 297 | 0·27 |
| 45 | 5,182 | 5·77 | 476 | 0·49 |  | 3,770 | 4·20 | 397 | 0·41 |  | 3,783 | 4·21 | 529 | 0·55 |
| 50 | 8,499 | 10·92 | 800 | 0·94 |  | 5,639 | 7·25 | 591 | 0·70 |  | 5,692 | 7·31 | 763 | 0·90 |
| 55 | 10,599 | 16·46 | 1,136 | 1·57 |  | 6,365 | 9·88 | 786 | 1·09 |  | 6,517 | 10·12 | 1,014 | 1·40 |
| 60 | 11,157 | 21·56 | 1,314 | 2·19 |  | 6,148 | 11·88 | 746 | 1·25 |  | 6,070 | 11·73 | 1,211 | 2·02 |
| 65 | 10,040 | 25·69 | 1,231 | 2·62 |  | 4,894 | 12·52 | 780 | 1·66 |  | 4,977 | 12·73 | 1,250 | 2·66 |
| 70 | 7,773 | 28·56 | 1,164 | 3·34 |  | 3,163 | 11·62 | 708 | 2·03 |  | 3,601 | 13·23 | 1,292 | 3·71 |
| 75 | 5,330 | 30·03 | 953 | 3·81 |  | 2,012 | 11·33 | 620 | 2·48 |  | 2,504 | 14·11 | 1,364 | 5·46 |
| 80 | 5,240 | 31·95 | 1,398 | 4·95 |  | 1,889 | 11·52 | 1,082 | 3·83 |  | 2,770 | 16·89 | 3,014 | 10·67 |
| **Total / ASMR** | 66,060 | 19·23 | 8,731 | 2·24 |  | 35,748 | 9·11 | 5,945 | 1·52 |  | 37,957 | 10·26 | 10,734 | 3·07 |
|  |  |  |  |  |  |  |  |  |  |  |  |  |  |  |
|  | **South** | | | | | | | | | | | | | |
|  | **Laryngeal Cancer** | | | |  | **Oropharyngeal Cancer** | | | |  | **Oral Cavity Cancer** | | | |
|  | **Male** | | **Female** | |  | **Male** | | **Female** | |  | **Male** | | **Female** | |
| **Age Group** | n | ASpMR | n | ASpMR |  | n | ASpMR | n | ASpMR |  | n | ASpMR | n | ASpMR |
| 40 | 807 | 2·23 | 80 | 0·21 |  | 568 | 1·57 | 47 | 0·13 |  | 615 | 1·70 | 91 | 0·24 |
| 45 | 1,928 | 6·09 | 184 | 0·56 |  | 1,186 | 3·75 | 99 | 0·30 |  | 1,307 | 4·13 | 136 | 0·41 |
| 50 | 3,247 | 11·74 | 291 | 1·03 |  | 1,810 | 6·55 | 159 | 0·54 |  | 1,877 | 6·79 | 208 | 0·71 |
| 55 | 4,071 | 17·60 | 404 | 1·71 |  | 2,131 | 9·21 | 233 | 0·93 |  | 2,157 | 9·33 | 307 | 1·23 |
| 60 | 4,275 | 23·01 | 470 | 2·43 |  | 1,945 | 10·47 | 212 | 1·03 |  | 2,080 | 11·19 | 302 | 1·47 |
| 65 | 3,742 | 26·75 | 470 | 3·13 |  | 1,571 | 11·23 | 207 | 1·28 |  | 1,698 | 12·14 | 344 | 2·14 |
| 70 | 2,943 | 30·34 | 374 | 3·34 |  | 1,044 | 10·76 | 228 | 1·92 |  | 1,249 | 12·88 | 352 | 2·96 |
| 75 | 2,005 | 32·44 | 317 | 3·99 |  | 719 | 11·63 | 177 | 2·11 |  | 892 | 14·43 | 348 | 4·15 |
| 80 | 1,700 | 31·55 | 387 | 4·63 |  | 578 | 10·73 | 342 | 3·80 |  | 984 | 18·26 | 792 | 8·81 |
| **Total / ASMR** | 24,718 | 20·19 | 3,148 | 2·34 |  | 11,552 | 8·43 | 1,704 | 1·34 |  | 12,859 | 10·09 | 2,880 | 2·46 |
|  |  |  |  |  |  |  |  |  |  |  |  |  |  |  |
|  | **Mid-west** | | | | | | | | | | | | | |
|  | **Laryngeal Cancer** | | | |  | **Oropharyngeal Cancer** | | | |  | **Oral Cavity Cancer** | | | |
|  | **Male** | | **Female** | |  | **Male** | | **Female** | |  | **Male** | | **Female** | |
| **Age Group** | n | ASpMR | n | ASpMR |  | n | ASpMR | n | ASpMR |  | n | ASpMR | n | ASpMR |
| 40 | 216 | 1·27 | 25 | 0·14 |  | 209 | 1·23 | 32 | 0·18 |  | 180 | 1·06 | 38 | 0·22 |
| 45 | 487 | 3·42 | 62 | 0·43 |  | 371 | 2·61 | 51 | 0·35 |  | 369 | 2·59 | 78 | 0·54 |
| 50 | 813 | 6·86 | 112 | 0·93 |  | 550 | 4·64 | 73 | 0·61 |  | 545 | 4·60 | 90 | 0·75 |
| 55 | 1,063 | 11·3 | 117 | 1·21 |  | 610 | 6·48 | 75 | 0·78 |  | 662 | 7·04 | 123 | 1·28 |
| 60 | 1,082 | 14·91 | 146 | 1·95 |  | 550 | 7·58 | 73 | 0·98 |  | 572 | 7·88 | 116 | 1·55 |
| 65 | 970 | 18·16 | 156 | 2·81 |  | 415 | 7·77 | 76 | 1·37 |  | 472 | 8·84 | 120 | 2·16 |
| 70 | 786 | 21·46 | 100 | 2·56 |  | 319 | 8·71 | 65 | 1·67 |  | 375 | 10·24 | 124 | 3·18 |
| 75 | 562 | 23·75 | 101 | 3·82 |  | 210 | 8·87 | 76 | 2·87 |  | 269 | 11·37 | 132 | 4·99 |
| 80 | 556 | 25·31 | 107 | 3·86 |  | 203 | 9·24 | 101 | 3·65 |  | 298 | 13·57 | 229 | 8·27 |
| **Total / ASMR** | 6,535 | 14·05 | 926 | 1·97 |  | 3,437 | 6·35 | 622 | 1·38 |  | 3,742 | 7·47 | 1,055 | 2·55 |
|  |  |  |  |  |  |  |  |  |  |  |  |  |  |  |
|  | **Northeast** | | | | | | | | | | | | | |
|  | **Laryngeal Cancer** | | | |  | **Oropharyngeal Cancer** | | | |  | **Oral Cavity Cancer** | | | |
|  | **Male** | | **Female** | |  | **Male** | | **Female** | |  | **Male** | | **Female** | |
| **Age Group** | n | ASpMR | n | ASpMR |  | n | ASpMR | n | ASpMR |  | n | ASpMR | n | ASpMR |
| 40 | 606 | 1·05 | 92 | 0·15 |  | 501 | 0·87 | 99 | 0·16 |  | 704 | 1·22 | 136 | 0·22 |
| 45 | 1,333 | 2·74 | 152 | 0·28 |  | 963 | 1·98 | 148 | 0·28 |  | 1,294 | 2·66 | 255 | 0·48 |
| 50 | 2,160 | 5·19 | 265 | 0·57 |  | 1,394 | 3·35 | 212 | 0·46 |  | 1,861 | 4·47 | 365 | 0·79 |
| 55 | 2,677 | 7·84 | 401 | 1·03 |  | 1,601 | 4·69 | 238 | 0·61 |  | 2,004 | 5·87 | 475 | 1·22 |
| 60 | 2,985 | 10·73 | 435 | 1·36 |  | 1,479 | 5·31 | 307 | 0·96 |  | 2,005 | 7·20 | 609 | 1·91 |
| 65 | 2,849 | 12·64 | 452 | 1·74 |  | 1,197 | 5·31 | 292 | 1·12 |  | 1,710 | 7·59 | 703 | 2·70 |
| 70 | 2,618 | 15·69 | 479 | 2·43 |  | 886 | 5·31 | 326 | 1·65 |  | 1,470 | 8·81 | 871 | 4·41 |
| 75 | 1,897 | 16·28 | 446 | 3·09 |  | 706 | 6·06 | 316 | 2·19 |  | 1,170 | 10·04 | 828 | 5·74 |
| 80 | 2,409 | 19·65 | 689 | 4·01 |  | 832 | 6·79 | 575 | 3·35 |  | 1,786 | 14·57 | 2248 | 13·09 |
| **Total / ASMR** | 19,534 | 10·20 | 3,411 | 1·63 |  | 9,559 | 4·41 | 2,513 | 1·20 |  | 14,004 | 6·94 | 5·97 | 6,490 |
|  |  |  |  |  |  |  |  |  |  |  |  |  |  |  |
|  | **North** | | | | | | | | | | | | | |
|  | **Laryngeal Cancer** | | | |  | **Oropharyngeal Cancer** | | | |  | **Oral Cavity Cancer** | | | |
|  | **Male** | | **Female** | |  | **Male** | | **Female** | |  | **Male** | | **Female** | |
| **Age Group** | n | ASpMR | n | ASpMR |  | n | ASpMR | n | ASpMR |  | n | ASpMR | n | ASpMR |
| 40 | 118 | 0·73 | 34 | 0·22 |  | 76 | 0·47 | 15 | 0·10 |  | 81 | 0·50 | 32 | 0·21 |
| 45 | 246 | 1·88 | 29 | 0·23 |  | 152 | 1·16 | 15 | 0·12 |  | 170 | 1·30 | 35 | 0·28 |
| 50 | 365 | 3·42 | 48 | 0·47 |  | 178 | 1·67 | 23 | 0·23 |  | 217 | 2·03 | 62 | 0·61 |
| 55 | 581 | 6·90 | 66 | 0·82 |  | 272 | 3·23 | 46 | 0·57 |  | 316 | 3·75 | 76 | 0·94 |
| 60 | 670 | 10·26 | 85 | 1·36 |  | 239 | 3·66 | 50 | 0·80 |  | 345 | 5·28 | 72 | 1·15 |
| 65 | 681 | 13·99 | 85 | 1·80 |  | 222 | 4·56 | 54 | 1·14 |  | 283 | 5·81 | 98 | 2·07 |
| 70 | 523 | 15·67 | 87 | 2·60 |  | 167 | 5·00 | 43 | 1·29 |  | 238 | 7·13 | 117 | 3·50 |
| 75 | 437 | 20·06 | 84 | 3·61 |  | 118 | 5·42 | 53 | 2·28 |  | 154 | 7·07 | 134 | 5·76 |
| 80 | 478 | 22·79 | 130 | 4·96 |  | 134 | 6·39 | 82 | 3·13 |  | 234 | 11·15 | 236 | 9·00 |
| **Total / ASMR** | 4,099 | 10·63 | 648 | 1·78 |  | 1,558 | 3·51 | 381 | 1·07 |  | 2,038 | 4·89 | 862 | 2·61 |

**Table 5.** Number of deaths age-specific mortality rates (ASpMR), and age-standardized mortality rates (ASMR) (per 100,000 persons) for laryngeal, oropharyngeal and oral cavity cancer by sex and ethnicity in Brazil, 2000–2023.

|  | **White** | | | | | | | | | | | | | |
| --- | --- | --- | --- | --- | --- | --- | --- | --- | --- | --- | --- | --- | --- | --- |
|  | **Laryngeal Cancer** | | | |  | **Oropharyngeal Cancer** | | | |  | **Oral Cavity Cancer** | | | |
|  | **Male** | | **Female** | |  | **Male** | | **Female** | |  | **Male** | | **Female** | |
| **Age Group** | n | ASpMR | n | ASpMR |  | n | ASpMR | n | ASpMR |  | n | ASpMR | n | ASpMR |
| 40 | 1,064 | 1·48 | 105 | 0·13 |  | 777 | 1·08 | 93 | 0·12 |  | 848 | 1·18 | 171 | 0·21 |
| 45 | 2,883 | 4·47 | 261 | 0·36 |  | 1,749 | 2·71 | 179 | 0·25 |  | 1,944 | 3·02 | 292 | 0·40 |
| 50 | 5,162 | 9·01 | 451 | 0·69 |  | 2,907 | 5·08 | 279 | 0·43 |  | 3,035 | 5·30 | 427 | 0·66 |
| 55 | 6,859 | 14·05 | 679 | 1·20 |  | 3,658 | 7·49 | 396 | 0·70 |  | 3,824 | 7·83 | 596 | 1·06 |
| 60 | 7,510 | 18·62 | 876 | 1·83 |  | 3,514 | 8·71 | 391 | 0·82 |  | 3,850 | 9·55 | 738 | 1·54 |
| 65 | 7,059 | 22·86 | 872 | 2·29 |  | 2,975 | 9·63 | 448 | 1·18 |  | 3,318 | 10·74 | 842 | 2·21 |
| 70 | 5,951 | 25·63 | 861 | 2·85 |  | 2,054 | 8·85 | 468 | 1·55 |  | 2,582 | 11·12 | 918 | 3·03 |
| 75 | 4,325 | 27·61 | 725 | 3·28 |  | 1,408 | 8·99 | 413 | 1·87 |  | 1,800 | 11·49 | 1031 | 4·66 |
| 80 | 4,682 | 29·40 | 1,193 | 4·34 |  | 1,386 | 8·70 | 809 | 2·94 |  | 2,390 | 15·01 | 2830 | 10·30 |
| **Total / ASMR** | 45,495 | 12·13 | 6,023 | 1·21 |  | 20,428 | 5·54 | 3,476 | 0·68 |  | 23,591 | 6·32 | 7,845 | 1·41 |
|  |  |  |  |  |  |  |  |  |  |  |  |  |  |  |
|  | **Black** | | | | | | | | | | | | | |
|  | **Laryngeal Cancer** | | | |  | **Oropharyngeal Cancer** | | | |  | **Oral Cavity Cancer** | | | |
|  | **Male** | | **Female** | |  | **Male** | | **Female** | |  | **Male** | | **Female** | |
| **Age Group** | n | ASpMR | n | ASpMR |  | n | ASpMR | n | ASpMR |  | n | ASpMR | n | ASpMR |
| 40 | 226 | 1·47 | 38 | 0·27 |  | 184 | 1·19 | 34 | 0·24 |  | 231 | 1·50 | 36 | 0·26 |
| 45 | 527 | 3·97 | 72 | 0·58 |  | 400 | 3·01 | 64 | 0·52 |  | 376 | 2·83 | 66 | 0·53 |
| 50 | 814 | 7·13 | 101 | 0·91 |  | 575 | 5·04 | 85 | 0·76 |  | 589 | 5·16 | 127 | 1·14 |
| 55 | 960 | 10·51 | 133 | 1·43 |  | 640 | 7·01 | 109 | 1·17 |  | 643 | 7·04 | 153 | 1·64 |
| 60 | 1,012 | 14·10 | 155 | 2·01 |  | 609 | 8·48 | 96 | 1·24 |  | 565 | 7·87 | 154 | 1·99 |
| 65 | 895 | 16·94 | 123 | 2·06 |  | 453 | 8·58 | 84 | 1·41 |  | 469 | 8·88 | 135 | 2·27 |
| 70 | 652 | 17·58 | 107 | 2·44 |  | 268 | 7·23 | 72 | 1·64 |  | 308 | 8·30 | 131 | 2·98 |
| 75 | 453 | 19·11 | 91 | 3·05 |  | 159 | 6·71 | 48 | 1·61 |  | 216 | 9·11 | 111 | 3·73 |
| 80 | 446 | 18·59 | 127 | 3·63 |  | 181 | 7·54 | 93 | 2·66 |  | 232 | 9·67 | 233 | 6·66 |
| **Total / ASMR** | 5,985 | 9·08 | 947 | 1·29 |  | 3,469 | 5·21 | 685 | 0·93 |  | 3,629 | 5·42 | 1,146 | 1·50 |
|  |  |  |  |  |  |  |  |  |  |  |  |  |  |  |
|  | **Brown** | | | | | | | | | | | | | |
|  | **Laryngeal Cancer** | | | |  | **Oropharyngeal Cancer** | | | |  | **Oral Cavity Cancer** | | | |
|  | **Male** | | **Female** | |  | **Male** | | **Female** | |  | **Male** | | **Female** | |
| **Age Group** | n | ASpMR | n | ASpMR |  | n | ASpMR | n | ASpMR |  | n | ASpMR | n | ASpMR |
| 40 | 776 | 1·20 | 109 | 0·16 |  | 671 | 1·04 | 114 | 0·17 |  | 744 | 1·15 | 139 | 0·21 |
| 45 | 1,818 | 3·29 | 196 | 0·34 |  | 1,303 | 2·36 | 174 | 0·3 |  | 1,418 | 2·57 | 245 | 0·42 |
| 50 | 2,869 | 6·17 | 364 | 0·73 |  | 1,967 | 4·23 | 267 | 0·54 |  | 2,087 | 4·49 | 402 | 0·81 |
| 55 | 3,728 | 9·93 | 495 | 1·20 |  | 2,216 | 5·90 | 322 | 0·78 |  | 2,502 | 6·66 | 462 | 1·12 |
| 60 | 3,948 | 13·40 | 566 | 1·73 |  | 2,039 | 6·92 | 311 | 0·95 |  | 2,228 | 7·56 | 523 | 1·60 |
| 65 | 3,559 | 16·24 | 530 | 2·13 |  | 1,53 | 6·98 | 297 | 1·20 |  | 1,802 | 8·22 | 549 | 2·21 |
| 70 | 2,791 | 18·17 | 458 | 2·57 |  | 1,046 | 6·81 | 267 | 1·50 |  | 1,320 | 8·59 | 626 | 3·52 |
| 75 | 2,022 | 20·70 | 394 | 3·33 |  | 699 | 7·16 | 249 | 2·10 |  | 1,006 | 10·3 | 573 | 4·84 |
| 80 | 2,158 | 22·17 | 557 | 4·15 |  | 740 | 7·60 | 436 | 3·25 |  | 1,231 | 12·65 | 1,256 | 9·36 |
| **Total / ASMR** | 23,669 | 8·72 | 3,669 | 1·17 |  | 12,211 | 4·44 | 2,437 | 0·76 |  | 14,338 | 5·20 | 4,775 | 1·46 |

**Table 6.** Average annual percent change (AAPC) in mortality from laryngeal, oropharyngeal, and oral cavity cancer by sex in Brazilian regions, 1980–2023.

| **Male** | | | | | | | |  | **Female** | | | | | | | |
| --- | --- | --- | --- | --- | --- | --- | --- | --- | --- | --- | --- | --- | --- | --- | --- | --- |
| **Laryngeal Cancer** | | | | | | | |  | **Laryngeal Cancer** | | | | | | | |
| **Cohort** | **Segment** | **Lower Endpoint** | **Upper Endpoint** | **APC** | **Lower CI** | **Upper CI** | **P-Value** |  | **Cohort** | **Segment** | **Lower Endpoint** | **Upper Endpoint** | **APC** | **Lower CI** | **Upper CI** | **P-Value** |
| Brazil | 1 | 1980 | 2023 | -0·109 | -0·251 | 0·034 | 0·13 |  | Brazil | 1 | 1980 | 2023 | -0·359* | -0·602 | -0·117 | 0·003 |
| Mid-West | 1 | 1980 | 2023 | 0·973* | 0·656 | 1·289 | <0·001 |  | Mid-West | 1 | 1980 | 2023 | 1·028* | 0·038 | 2·029 | 0·042 |
| Northeast | 1 | 1980 | 2023 | 3·183* | 2·928 | 3·439 | <0·001 |  | Northeast | 1 | 1980 | 2023 | 3·070* | 2·564 | 3·575 | <0·001 |
| North | 1 | 1980 | 2023 | 0·725* | 0·218 | 1·234 | 0·006 |  | North | 1 | 1980 | 2023 | 0·415 | -0·606 | 1·448 | 0·42 |
| Southeast | 1 | 1980 | 2023 | -0·975* | -1·120 | -0·830 | <0·001 |  | Southeast | 1 | 1980 | 2023 | -1·282* | -1·571 | -0·992 | <0·001 |
| South | 1 | 1980 | 2023 | -0·742* | -0·932 | -0·551 | <0·001 |  | South | 1 | 1980 | 2023 | -1·119* | -1·400 | -0·837 | <0·001 |
| **Oropharyngeal Cancer** | | | | | | | |  | **Oropharyngeal Cancer** | | | | | | | |
| **Cohort** | **Segment** | **Lower Endpoint** | **Upper Endpoint** | **APC** | **Lower CI** | **Upper CI** | **P-Value** |  | **Cohort** | **Segment** | **Lower Endpoint** | **Upper Endpoint** | **APC** | **Lower CI** | **Upper CI** | **P-Value** |
| Brazil | 1 | 1980 | 2023 | 1·529* | 1·292 | 1·767 | <0·001 |  | Brazil | 1 | 1980 | 2023 | 1·036* | 0·767 | 1·307 | <0·001 |
| Mid-West | 1 | 1980 | 2023 | 3·089* | 2·474 | 3·709 | <0·001 |  | Mid-West | 1 | 1980 | 2023 | 1·431* | 0·507 | 2·363 | 0·003 |
| Northeast | 1 | 1980 | 2023 | 4·357* | 4·055 | 4·660 | <0·001 |  | Northeast | 1 | 1980 | 2023 | 3·586* | 3·179 | 3·995 | <0·001 |
| North | 1 | 1980 | 2023 | 3·602* | 2·669 | 4·545 | <0·001 |  | North | 1 | 1980 | 2023 | 1·699* | 0·815 | 2·592 | <0·001 |
| Southeast | 1 | 1980 | 2023 | 0·9228 | 0·603 | 1·243 | <0·001 |  | Southeast | 1 | 1980 | 2023 | 0·241 | -0·096 | 0·578 | 0·16 |
| South | 1 | 1980 | 2023 | 0·824* | 0·544 | 1·105 | <0·001 |  | South | 1 | 1980 | 2023 | 1·154* | 0·528 | 1·784 | <0·001 |
| **Oral Cavity Cancer** | | | | | | | |  | **Oral Cavity Cancer** | | | | | | | |
| **Cohort** | **Segment** | **Lower Endpoint** | **Upper Endpoint** | **APC** | **Lower CI** | **Upper CI** | **P-Value** |  | **Cohort** | **Segment** | **Lower Endpoint** | **Upper Endpoint** | **APC** | **Lower CI** | **Upper CI** | **P-Value** |
| Brazil | 1 | 1980 | 2023 | -0·303* | -0·457 | -0·149 | <0·001 |  | Brazil | 1 | 1980 | 2023 | -0·027 | -0·209 | 0·155 | 0·77 |
| Mid-West | 1 | 1980 | 2023 | 0·568* | 0·212 | 0·920 | 0·002 |  | Mid-West | 1 | 1980 | 2023 | 1·215 | -0·401 | 2·809 | 0·12 |
| Northeast | 1 | 1980 | 2023 | 2·423* | 2·107 | 2·741 | <0·001 |  | Northeast | 1 | 1980 | 2023 | 1·734* | 1·312 | 2·159 | <0·001 |
| North | 1 | 1980 | 2023 | 1·124* | 0·662 | 1·589 | <0·001 |  | North | 1 | 1980 | 2023 | -0·106 | -0·979 | 0·775 | 0·81 |
| Southeast | 1 | 1980 | 2023 | -1·153* | -1·354 | -0·952 | <0·001 |  | Southeast | 1 | 1980 | 2023 | -0·926* | -1·191 | -0·659 | <0·001 |
| South | 1 | 1980 | 2023 | -0·974* | -1·180 | -0·762 | <0·001 |  | South | 1 | 1980 | 2023 | 0·248 | -0·072 | 0·569 | 0·13 |

*AAPC statistically significant at p < 0.05.

**Table 7.** Average annual percent change (AAPC) in mortality from laryngeal, oropharyngeal, and oral cavity cancer by sex and ethnicity in Brazil, 2000–2023.

| **Male** | | | | | | | |  | **Female** | | | | | | | |
| --- | --- | --- | --- | --- | --- | --- | --- | --- | --- | --- | --- | --- | --- | --- | --- | --- |
| **Laryngeal Cancer** | | | | | | | |  | **Laryngeal Cancer** | | | | | | | |
| **Cohort** | **Segment** | **Lower Endpoint** | **Upper Endpoint** | **APC** | **Lower CI** | **Upper CI** | **P-Value** |  | **Cohort** | **Segment** | **Lower Endpoint** | **Upper Endpoint** | **APC** | **Lower CI** | **Upper CI** | **P-Value** |
| White | 1 | 2000 | 2023 | -1·627* | -1·991 | -1·261 | <0·001 |  | White | 1 | 2000 | 2023 | -1·032* | -1·522 | -0·540 | <0·001 |
| Black | 1 | 2000 | 2023 | -0·857* | -1·301 | -0·411 | <0·001 |  | Black | 1 | 2000 | 2023 | -0·456 | -1·935 | 1·045 | 0·53 |
| Brown | 1 | 2000 | 2023 | 2·437* | 1·802 | 3·075 | <0·001 |  | Brown | 1 | 2000 | 2023 | 1·810* | 1·089 | 2·537 | <0·001 |
| **Oropharyngeal Cancer** | | | | | | | |  | **Oropharyngeal Cancer** | | | | | | | |
| **Cohort** | **Segment** | **Lower Endpoint** | **Upper Endpoint** | **APC** | **Lower CI** | **Upper CI** | **P-Value** |  | **Cohort** | **Segment** | **Lower Endpoint** | **Upper Endpoint** | **APC** | **Lower CI** | **Upper CI** | **P-Value** |
| White | 1 | 2000 | 2023 | -0·609* | -0·979 | -0·237 | 0·002 |  | White | 1 | 2000 | 2023 | 0·040 | -0·399 | 0·464 | 0·90 |
| Black | 1 | 2000 | 2023 | -0·337 | -0·958 | 0·288 | 0·27 |  | Brown | 1 | 2000 | 2023 | -0·434 | -1·480 | 0·623 | 0·40 |
| Brown | 1 | 2000 | 2023 | 2·378* | 1·934 | 2·824 | <0·001 |  | Black | 1 | 2000 | 2023 | 1·866* | 0·894 | 2·812 | <0·001 |
| **Oral Cavity Cancer** | | | | | | | |  | **Oral Cavity Cancer** | | | | | | | |
| **Cohort** | **Segment** | **Lower Endpoint** | **Upper Endpoint** | **APC** | **Lower CI** | **Upper CI** | **P-Value** |  | **Cohort** | **Segment** | **Lower Endpoint** | **Upper Endpoint** | **APC** | **Lower CI** | **Upper CI** | **P-Value** |
| White | 1 | 2000 | 2023 | -1·014* | -1·319 | -0·709 | <0·001 |  | White | 1 | 2000 | 2023 | 0·062 | -0·337 | 0·463 | 0·75 |
| Black | 1 | 2000 | 2023 | -1·324* | -1·924 | -0·720 | <0·001 |  | Brown | 1 | 2000 | 2023 | -1·862* | -2·665 | -1·097 | <0·001 |
| Brown | 1 | 2000 | 2023 | 1·605* | 1·119 | 2·094 | <0·001 |  | Black | 1 | 2000 | 2023 | 0·8889* | 0·064 | 1·720 | 0·036 |

*AAPC statistically significant at p < 0.05.

**Table 8.** Average annual percent change (AAPC) in mortality from laryngeal, oropharyngeal, and oral cavity cancer by sex and age group in Brazil, 1980–2023.

|  |  | **Male** | | |  | **Female** | | |
| --- | --- | --- | --- | --- | --- | --- | --- | --- |
| **Age Group** | | **APC** | **Lower CI** | **Upper CI** |  | **APC** | **Lower CI** | **Upper CI** |
| **Laryngeal Cancer** | 40 | -1·52* | -1·95 | -1·10 |  | -2·74* | -3·34 | -2·14 |
|  | 45 | -1·26* | -1·61 | -0·90 |  | -1·19* | -1·63 | -0·74 |
|  | 50 | -0·97* | -1·21 | -0·73 |  | -0·86* | -1·28 | -0·43 |
|  | 55 | -0·59* | -0·80 | -0·39 |  | -0·51* | -0·87 | -0·15 |
|  | 60 | -0·24* | -0·36 | -0·12 |  | -0·08 | -0·44 | 0·27 |
|  | 65 | 0·18* | 0·00 | 0·35 |  | -0·25 | -0·57 | 0·06 |
|  | 70 | 0·10 | -0·10 | 0·30 |  | -0·18 | -0·51 | 0·15 |
|  | 75 | 0·22* | 0·08 | 0·37 |  | -0·53* | -0·93 | -0·13 |
|  | 80 | 0·28* | 0·01 | 0·54 |  | 0·07 | -0·36 | 0·51 |
|  |  |  |  |  |  |  |  |  |
| **Oropharyngeal Cancer** | 40 | 0·36 | -0·15 | 0·87 |  | 2·29* | 1·33 | 3·26 |
|  | 45 | 0·54* | 0·09 | 0·99 |  | 1·94* | 1·13 | 2·74 |
|  | 50 | 1·34* | 0·91 | 1·77 |  | 2·26* | 1·25 | 3·28 |
|  | 55 | 1·71* | 1·38 | 2·03 |  | 1·86* | 1·37 | 2·36 |
|  | 60 | 1·99* | 1·66 | 2·32 |  | 1·58* | 1·05 | 2·12 |
|  | 65 | 2·15* | 1·83 | 2·47 |  | 1·35* | 0·90 | 1·79 |
|  | 70 | 1·96* | 1·63 | 2·29 |  | 0·64* | 0·24 | 1·04 |
|  | 75 | 1·18* | 0·97 | 1·39 |  | 0·83* | 0·36 | 1·30 |
|  | 80 | 1·32* | 1·01 | 1·62 |  | 1·15* | 0·56 | 1·74 |
|  |  |  |  |  |  |  |  |  |
| **Oral Cavity Cancer** | 40 | -1·66* | -2·02 | -1·29 |  | 0·18 | -0·66 | 1·01 |
|  | 45 | -1·45* | -1·71 | -1·20 |  | -0·57* | -1·00 | -0·14 |
|  | 50 | -0·97* | -1·15 | -0·80 |  | 0·46* | 0·10 | 0·82 |
|  | 55 | -0·25* | -0·47 | -0·04 |  | -0·45* | -0·77 | -0·14 |
|  | 60 | -0·08 | -0·24 | 0·09 |  | 0·00 | -0·37 | 0·36 |
|  | 65 | 0·37* | 0·17 | 0·58 |  | -0·25 | -0·61 | 0·10 |
|  | 70 | -0·13 | -0·32 | 0·07 |  | -0·49* | -0·91 | -0·07 |
|  | 75 | -0·18 | -0·45 | 0·09 |  | -0·40* | -0·77 | -0·03 |
|  | 80 | -0·28 | -0·64 | 0·08 |  | 0·62* | 0·35 | 0·90 |

*AAPC statistically significant at p < 0.05.

Green indicates negative AAPC (decreasing trend), orange indicates positive AAPC (increasing trend), and white indicates non-significant results (p > 0.05)

**Table 9.** Average annual percent change (AAPC) in mortality from laryngeal, oropharyngeal, and oral cavity cancer by sex and age group in Brazilian regions, 1980–2023.

|  |  | | | **Southeast** | | | | | | | | | | | | |  | | **South** | | | | | | | | | | | | | |  | | **Mid-west** | | | | | | | | | | | | | |  | | **Northeast** | | | | | | | | | | | | | |  | | **North** | | | | | | | | | | | |
| --- | --- | --- | --- | --- | --- | --- | --- | --- | --- | --- | --- | --- | --- | --- | --- | --- | --- | --- | --- | --- | --- | --- | --- | --- | --- | --- | --- | --- | --- | --- | --- | --- | --- | --- | --- | --- | --- | --- | --- | --- | --- | --- | --- | --- | --- | --- | --- | --- | --- | --- | --- | --- | --- | --- | --- | --- | --- | --- | --- | --- | --- | --- | --- | --- | --- | --- | --- | --- | --- | --- | --- | --- | --- | --- | --- | --- | --- | --- |
|  |  | | | **Male** | | | | | |  | **Female** | | | | | |  | | **Male** | | | | | |  | | **Female** | | | | | |  | | **Male** | | | | | |  | | **Female** | | | | | |  | | **Male** | | | | | |  | **Female** | | | | | | |  | | **Male** | | | | | |  | **Female** | | | | |
|  | **Age Group** | | | **APC** | **Lower CI** | | **Upper CI** | | |  | **APC** | | **Lower CI** | | **Upper CI** | |  | | **APC** | | **Lower CI** | | **Upper CI** | |  | | **APC** | | **Lower CI** | | **Upper CI** | |  | | **APC** | | **Lower CI** | | **Upper CI** | |  | | **APC** | | **Lower CI** | | **Upper CI** | |  | | **APC** | | **Lower CI** | | **Upper CI** | |  | **APC** | | **Lower CI** | | **Upper CI** | | |  | | **APC** | **Lower CI** | | **Upper CI** | | |  | **APC** | **Lower CI** | | **Upper CI** | |
| **Laryngeal Cancer** | | **40** | -2·45* | | | -2·95 | | -1·94 |  | | | -3·32* | | -4·42 | | -2·22 | |  | | -2·23* | | -2·96 | | -1·49 | |  | | -4·27* | | -5·94 | | -2·54 | |  | | 1·33* | | 0·15 | | 2·49 | |  | | -3·32* | | -4·20 | | -2·44 | |  | | 2·88* | | 2·10 | | 3·66 |  | | 0·25 | | -1·17 | | 1·71 |  | | 0·00 | | | -1·07 | | 1·09 |  | | -3·08* | | -4·34 | | -1·81 |
|  |  | **45** | -2·01* | | | -2·47 | | -1·55 |  | | | -1·48* | | -2·17 | | -0·78 | |  | | -2·28* | | -2·88 | | -1·68 | |  | | -1·62* | | -3·00 | | -0·22 | |  | | 2·23* | | 1·33 | | 3·13 | |  | | -1·08 | | -2·50 | | 0·38 | |  | | 3·18* | | 2·58 | | 3·78 |  | | 1·60* | | 0·34 | | 2·87 |  | | -0·01 | | | -1·03 | | 1·02 |  | | -2·83* | | -3·80 | | -1·87 |
|  |  | **50** | -1·59* | | | -1·86 | | -1·32 |  | | | -1·70* | | -2·23 | | -1·16 | |  | | -1·71* | | -2·06 | | -1·35 | |  | | -1·82* | | -2·80 | | -0·84 | |  | | 0·97* | | 0·32 | | 1·63 | |  | | 0·32 | | -1·07 | | 1·68 | |  | | 2·94* | | 2·51 | | 3·37 |  | | 3·79* | | 2·25 | | 5·35 |  | | 0·10 | | | -0·85 | | 1·05 |  | | -2·76* | | -4·29 | | -1·17 |
|  |  | **55** | -1·19* | | | -1·41 | | -0·96 |  | | | -1·09* | | -1·62 | | -0·56 | |  | | -1·29* | | -1·59 | | -1·00 | |  | | -1·35* | | -2·24 | | -0·45 | |  | | 1·38* | | 0·72 | | 2·04 | |  | | -0·06 | | -1·15 | | 1·01 | |  | | 2·85* | | 2·35 | | 3·36 |  | | 3·63* | | 2·59 | | 4·68 |  | | 1·02* | | | 0·11 | | 1·94 |  | | -1·29* | | -2·49 | | -0·07 |
|  |  | **60** | -0·82* | | | -0·97 | | -0·66 |  | | | -0·97* | | -1·38 | | -0·56 | |  | | -0·90* | | -1·12 | | -0·67 | |  | | -0·61 | | -1·57 | | 0·37 | |  | | 1·06* | | 0·42 | | 1·70 | |  | | 1·598 | | 0·14 | | 3·08 | |  | | 2·84* | | 2·49 | | 3·19 |  | | 4·49* | | 3·20 | | 5·80 |  | | 0·99* | | | 0·38 | | 1·62 |  | | -0·25 | | -1·39 | | 0·91 |
|  |  | **65** | -0·50* | | | -0·72 | | -0·28 |  | | | -1·08* | | -1·52 | | -0·62 | |  | | -0·79* | | -1·12 | | -0·47 | |  | | -1·14* | | -1·81 | | -0·47 | |  | | 1·31* | | 0·75 | | 1·89 | |  | | 0·71 | | -0·67 | | 2·12 | |  | | 3·31* | | 3·02 | | 3·60 |  | | 3·52* | | 2·44 | | 4·62 |  | | 1·74* | | | 0·87 | | 2·62 |  | | 0·09 | | -1·21 | | 1·40 |
|  |  | **70** | -0·81* | | | -1·03 | | -0·58 |  | | | -1·30* | | -1·80 | | -0·80 | |  | | -0·548 | | -0·87 | | -0·21 | |  | | -0·87* | | -1·59 | | -0·14 | |  | | 1·04* | | 0·26 | | 1·82 | |  | | 0·19 | | -1·11 | | 1·50 | |  | | 3·54* | | 3·11 | | 3·98 |  | | 4·19* | | 3·14 | | 5·25 |  | | 0·97* | | | 0·18 | | 1·75 |  | | 0·16 | | -1·02 | | 1·35 |
|  |  | **75** | -0·82* | | | -1·03 | | -0·61 |  | | | -1·85* | | -2·40 | | -1·30 | |  | | -0·23 | | -0·51 | | 0·05 | |  | | -0·54 | | -1·42 | | 0·35 | |  | | 0·54* | | 0·31 | | 1·39 | |  | | -0·83 | | -2·58 | | 1·01 | |  | | 3·44* | | 2·83 | | 4·06 |  | | 3·32* | | 2·34 | | 4·33 |  | | 1·64* | | | 0·69 | | 2·59 |  | | -0·58 | | -1·82 | | 0·69 |
|  |  | **80** | -0·97* | | | -1·26 | | -0·68 |  | | | -0·59* | | -1·13 | | -0·05 | |  | | 0·11 | | -0·27 | | 0·49 | |  | | -0·46 | | -1·77 | | 0·83 | |  | | 2·12* | | 1·23 | | 2·99 | |  | | -1·76 | | -2·90 | | -0·60 | |  | | 3·69* | | 3·16 | | 4·21 |  | | 3·90* | | 3·10 | | 4·65 |  | | 0·66 | | | -0·27 | | 1·60 |  | | -0·95 | | -2·37 | | 0·47 |
|  | |  |  | | |  | |  |  | | |  | |  | |  | |  | |  | |  | |  | |  | |  | |  | |  | |  | |  | |  | |  | |  | |  | |  | |  | |  | |  | |  | |  |  | |  | |  | |  |  | |  | | |  | |  |  | |  | |  | |  |
| **Oropharyngeal Cancer** | | **40** | -0·54 | | | -1·20 | | 0·11 |  | | | 1·56* | | 0·44 | | 2·68 | |  | | 0·28 | | -0·69 | | 1·25 | |  | | -0·84 | | -2·36 | | 0·71 | |  | | 3·53* | | 2·44 | | 4·64 | |  | | -1·10* | | -2·04 | | -0·16 | |  | | 4·52* | | 3·29 | | 5·75 |  | | 1·73* | | 0·28 | | 3·17 |  | | 1·12* | | | 0·02 | | 2·22 |  | | -2·26* | | -2·96 | | -1·56 |
|  |  | **45** | -0·09 | | | -0·63 | | 0·44 |  | | | 2·85* | | 1·83 | | 3·88 | |  | | -0·15 | | -0·79 | | 0·49 | |  | | -1·24 | | -2·84 | | 0·40 | |  | | 2·84* | | 1·51 | | 4·19 | |  | | -0·42 | | -1·50 | | 0·67 | |  | | 4·03* | | 3·37 | | 4·69 |  | | 2·03* | | 1·02 | | 3·06 |  | | 3·53* | | | 2·32 | | 4·71 |  | | -2·69* | | -3·55 | | -1·83 |
|  |  | **50** | 0·72* | | | 0·19 | | 1·26 |  | | | 2·02* | | 0·98 | | 3·08 | |  | | 0·99* | | 0·43 | | 1·56 | |  | | 0·76 | | -0·37 | | 1·90 | |  | | 4·25* | | 2·73 | | 5·84 | |  | | -0·22 | | -1·29 | | 0·87 | |  | | 4·62* | | 3·98 | | 5·26 |  | | 3·47* | | 2·29 | | 4·66 |  | | 2·15* | | | 1·06 | | 3·25 |  | | -2·48* | | -3·37 | | -1·58 |
|  |  | **55** | 1·23* | | | 0·77 | | 1·70 |  | | | 1·81* | | 1·17 | | 2·45 | |  | | 1·29* | | 0·91 | | 1·68 | |  | | 1·50* | | 0·43 | | 2·59 | |  | | 4·17* | | 2·87 | | 5·47 | |  | | -0·72 | | -1·95 | | 0·52 | |  | | 4·77* | | 4·12 | | 5·42 |  | | 2·98* | | 1·39 | | 4·63 |  | | 3·27* | | | 2·11 | | 4·45 |  | | -1·14 | | -2·32 | | 0·07 |
|  |  | **60** | 1·52* | | | 1·07 | | 1·97 |  | | | 1·34* | | 0·64 | | 2·05 | |  | | 1·55* | | 1·17 | | 1·94 | |  | | 2·64* | | 1·49 | | 3·80 | |  | | 5·10* | | 4·15 | | 6·05 | |  | | -0·97 | | -2·47 | | 0·56 | |  | | 4·27* | | 3·61 | | 4·94 |  | | 2·69* | | 1·70 | | 3·70 |  | | 4·29* | | | 3·31 | | 5·27 |  | | -1·32* | | -2·49 | | -0·13 |
|  |  | **65** | 1·48* | | | 1·13 | | 1·83 |  | | | 0·63* | | 0·08 | | 1·17 | |  | | 1·62* | | 1·14 | | 2·10 | |  | | 1·82* | | 0·37 | | 3·33 | |  | | 5·02* | | 3·73 | | 6·31 | |  | | -0·30 | | -1·78 | | 1·22 | |  | | 4·87* | | 4·26 | | 5·47 |  | | 4·11* | | 2·99 | | 5·23 |  | | 4·28* | | | 2·91 | | 5·65 |  | | -1·30 | | -2·62 | | 0·04 |
|  |  | **70** | 1·33* | | | 0·97 | | 1·69 |  | | | -0·06 | | -0·66 | | 0·55 | |  | | 1·34* | | 0·72 | | 1·97 | |  | | 0·43 | | -0·65 | | 1·55 | |  | | 2·02* | | 1·01 | | 3·05 | |  | | -1·72* | | -3·20 | | -0·20 | |  | | 6·30* | | 5·21 | | 7·39 |  | | 4·10* | | 3·14 | | 5·08 |  | | 2·14* | | | 0·64 | | 3·67 |  | | -1·26* | | -2·40 | | -0·11 |
|  |  | **75** | 0·72* | | | 0·35 | | 1·09 |  | | | -0·15 | | -0·85 | | 0·56 | |  | | 0·01 | | -0·66 | | 0·68 | |  | | 2·45* | | 1·03 | | 3·90, | |  | | 1·48* | | 0·42 | | 2·57 | |  | | -2·27* | | -3·67 | | -0·88 | |  | | 4·57* | | 3·95 | | 5·19 |  | | 3·05* | | 1·74 | | 4·4 |  | | 0·11 | | | -0·91 | | 1·17 |  | | -0·44 | | -1·57 | | 0·70 |
|  |  | **80** | 0·46* | | | 0·06 | | 0·86 |  | | | -0·06 | | -0·63 | | 0·52 | |  | | 1·03* | | 0·35 | | 1·70 | |  | | 0·82 | | -0·40 | | 2·06 | |  | | 1·81* | | 0·76 | | 2·88 | |  | | -0·24 | | -1·18 | | 0·72 | |  | | 3·98* | | 3·16 | | 4·80 |  | | 5·28* | | 4·36 | | 6·20 |  | | 1·14* | | | 0·03 | | 2·27 |  | | -1·29 | | -3·13 | | 0·59 |
|  | |  |  | | |  | |  |  | | |  | |  | |  | |  | |  | |  | |  | |  | |  | |  | |  | |  | |  | |  | |  | |  | |  | |  | |  | |  | |  | |  | |  |  | |  | |  | |  |  | |  | | |  | |  |  | |  | |  | |  |
| **Oral Cavity Cancer** | | **40** | -2·72* | | | -3·18 | | -2·24 |  | | | -0·54 | | -1·48 | | 0·40 | |  | | -1·82* | | -2·36 | | -1·28 | |  | | -0·92 | | -2·28 | | 0·44 | |  | | 0·61 | | -0·84 | | 2·12 | |  | | -1·13 | | -2·36 | | 0·11 | |  | | 1·99* | | 0·88 | | 3·12 |  | | 2·34* | | 0·91 | | 3·79 |  | | 0·17 | | | -1·16 | | 1·51 |  | | -2·00* | | -2·93 | | -1·07 |
|  |  | **45** | -2·46* | | | -2·73 | | -2·18 |  | | | -0·99* | | -1·56 | | -0·43 | |  | | -1·64* | | -2·13 | | -1·15 | |  | | -1·44* | | -2·71 | | -0·16 | |  | | 1·25* | | 0·12 | | 2·39 | |  | | -0·87 | | -2·33 | | 0·63 | |  | | 1·75* | | 1·16 | | 2·34 |  | | 1·83* | | 0·46 | | 3·25 |  | | 0·97 | | | -0·95 | | 2·96 |  | | -2·76* | | -3·99 | | -1·51 |
|  |  | **50** | -1·79* | | | -2·02 | | -1·56 |  | | | 0·08 | | -0·39 | | 0·53 | |  | | -1·39* | | -1·68 | | -1·10 | |  | | -0·53 | | -1·82 | | 0·78 | |  | | 2·23* | | 0·98 | | 3·48 | |  | | 0·62 | | -0·69 | | 1·91 | |  | | 1·96* | | 1·51 | | 2·42 |  | | 2·18* | | 1·07 | | 3·32 |  | | 2·32* | | | 1·35 | | 3·29 |  | | 0·86 | | -0·24 | | 1·97 |
|  |  | **55** | -0·94* | | | -1·24 | | -0·63 |  | | | -0·99* | | -1·46 | | -0·52 | |  | | -0·48* | | -0·79 | | -0·17 | |  | | -0·12 | | -1·19 | | 0·96 | |  | | 1·21* | | 0·78 | | 1·65 | |  | | -1·06 | | -2·26 | | 0·14 | |  | | 2·53* | | 2·16 | | 2·90 |  | | 1·61* | | 0·91 | | 2·30 |  | | 0·36 | | | -0·72 | | 1·45 |  | | -0·94 | | -2·45 | | 0·59 |
|  |  | **60** | -0·83* | | | -1·00 | | -0·65 |  | | | -0·84* | | -1·31 | | -0·37 | |  | | -0·45* | | -0·77 | | -0·13 | |  | | 0·86 | | -0·22 | | 1·95 | |  | | 0·92* | | 0·15 | | 1·70 | |  | | 0·55 | | -0·68 | | 1·79 | |  | | 2·88* | | 2·50 | | 3·26 |  | | 1·97* | | 1·33 | | 2·61 |  | | 1·82* | | | 0·98 | | 2·67 |  | | -1·72* | | -3·35 | | -0·05 |
|  |  | **65** | -0·45* | | | -0·75 | | -0·15 |  | | | -1·00* | | -1·42 | | -0·58 | |  | | -0·02 | | -0·34 | | 0·28 | |  | | 0·53 | | -0·34 | | 1·41 | |  | | 1·30* | | 0·61 | | 1·99 | |  | | 0·49 | | -0·72 | | 1·71 | |  | | 3·37* | | 2·87 | | 3·89 |  | | 1·72* | | 0·56 | | 2·89 |  | | 1·51* | | | 0·55 | | 2·47 |  | | -0·91 | | -2·48 | | 0·68 |
|  |  | **70** | -0·92* | | | -1·19 | | -0·66 |  | | | -1·34* | | -1·87 | | -0·80 | |  | | -0·66* | | -1·11 | | -0·20 | |  | | 0·00 | | -0·94 | | 0·96 | |  | | 0·59 | | -0·53 | | 1·69 | |  | | -0·48 | | -1·83 | | 0·90 | |  | | 2·41* | | 1·93 | | 2·90 |  | | 1·16* | | 0·49 | | 1·83 |  | | 0·42 | | | -0·55 | | 1·42 |  | | -0·76 | | -2·30 | | 0·80 |
|  |  | **75** | -0·93* | | | -1·38 | | -0·48 |  | | | -1·38* | | -1·87 | | -0·89 | |  | | -1·54* | | -2·04 | | -1·05 | |  | | 0·30 | | -0·61 | | 1·21 | |  | | 1·30* | | 0·33 | | 2·26 | |  | | 0·01 | | -1·50 | | 1·54 | |  | | 2·43* | | 1·86 | | 3·00 |  | | 1·60* | | 0·95 | | 2·26 |  | | 0·67 | | | -0·62 | | 1·98 |  | | -0·37 | | -1·72 | | 1·00 |
|  |  | **80** | -1·42* | | | -1·85 | | -0·98 |  | | | -0·39* | | -0·69 | | -0·09 | |  | | -1·27* | | -1·96 | | -0·59 | |  | | 1·21* | | 0·55 | | 1·89 | |  | | 0·25 | | -0·93 | | 1·44 | |  | | 1·21* | | 0·09 | | 2·35 | |  | | 2·63* | | 1·95 | | 3·31 |  | | 2·49* | | 1·95 | | 3·02 |  | | 1·47* | | | 0·22 | | 2·73 |  | | 0·01 | | -1·17 | | 1·19 |

*APC statistically significant at p < 0.05.

Green indicates negative AAPC (decreasing trend), orange indicates positive AAPC (increasing trend), and white indicates non-significant results (p > 0.05)

**Table 10.** Average annual percent change (AAPC) in mortality from laryngeal, oropharyngeal, and oral cavity cancer by sex and ethnicity in Brazil, 2000–2023.

|  |  | **White** | | | | | | |  | **Black** | | | | | | |  | **Brown** | | | | | | |
| --- | --- | --- | --- | --- | --- | --- | --- | --- | --- | --- | --- | --- | --- | --- | --- | --- | --- | --- | --- | --- | --- | --- | --- | --- |
|  |  | **Male** | | |  | **Female** | | |  | **Male** | | |  | **Female** | | |  | **Male** | | |  | **Female** | | |
|  | **Age Group** | **APC** | **Lower CI** | **Upper CI** |  | **APC** | **Lower CI** | **Upper CI** |  | **APC** | **Lower CI** | **Upper CI** |  | **APC** | **Lower CI** | **Upper CI** |  | **APC** | **Lower CI** | **Upper CI** |  | **APC** | **Lower CI** | **Upper CI** |
| **Laryngeal Cancer** | **40** | -4,77* | -5,60 | -3,94 |  | -7,36* | -9,55 | -5,11 |  | -3,56* | -5,10 | -2,01 |  | -4,87* | -7,54 | -2,22 |  | 0,23 | -0,76 | 1,23 |  | -1,84 | -5,33 | 1,77 |
|  | **45** | -4,82* | -5,50 | -4,14 |  | -3,12* | -4,65 | -1,57 |  | -3,98* | -5,16 | -2,87 |  | -2,23 | -5,31 | 0,73 |  | -0,05 | -0,93 | 0,85 |  | 0,28 | -1,16 | 1,75 |
|  | **50** | -3,33* | -4,02 | -2,63 |  | -2,70* | -4,19 | -1,19 |  | -1,75* | -3,08 | -0,39 |  | -2,97 | -5,94 | 0,10 |  | 1,14* | 0,17 | 2,12 |  | 1,67 | -0,03 | 3,32 |
|  | **55** | -1,94* | -2,64 | -1,24 |  | 0,13 | -1,01 | 1,29 |  | -1,92* | -2,83 | -1,00 |  | 0,24 | -3,07 | 3,65 |  | 2,40* | 1,37 | 3,43 |  | 0,99* | 0,11 | 1,89 |
|  | **60** | -1,20* | -1,56 | -0,83 |  | -1,80* | -2,95 | -0,71 |  | 0,32 | -0,39 | 1,04 |  | 1,66 | -0,83 | 4,21 |  | 2,85* | 2,33 | 3,38 |  | 2,98* | 1,33 | 4,61 |
|  | **65** | -0,86* | -1,30 | -0,42 |  | 0,27 | -0,66 | 1,19 |  | -0,49 | -1,31 | 0,29 |  | 0,49 | -2,16 | 3,22 |  | 3,02* | 2,31 | 3,74 |  | 2,94* | 1,32 | 4,54 |
|  | **70** | -0,79 | -1,16 | -0,42 |  | -1,50* | -2,67 | -0,35 |  | 0,13 | -0,65 | 0,86 |  | 1,84 | -1,01 | 4,76 |  | 2,87* | 2,24 | 3,51 |  | 1,80* | 0,29 | 3,28 |
|  | **75** | -0,62* | -1,15 | -0,09 |  | -0,34 | -1,23 | 0,56 |  | 0,82 | -1,13 | 2,71 |  | 0,98 | -2,66 | 4,53 |  | 2,80* | 2,12 | 3,49 |  | 2,56* | 0,78 | 4,38 |
|  | **80** | -0,34 | -0,98 | 0,29 |  | 0,56 | -0,37 | 1,50 |  | 2,51* | 1,24 | 3,79 |  | -2,17 | -4,41 | 0,12 |  | 4,40* | 3,57 | 5,23 |  | 2,23* | 0,63 | 3,86 |
|  |  |  |  |  |  |  |  |  |  |  |  |  |  |  |  |  |  |  |  |  |  |  |  |  |
| **Oropharyngeal Cancer** | **40** | -3,02* | -4,01 | -2,03 |  | -0,96 | -3,70 | 1,83 |  | -5,10* | -6,39 | -3,86 |  | -4,22 | -10,47 | 1,99 |  | 0,36 | -0,31 | 1,02 |  | 1,11 | -0,50 | 2,66 |
|  | **45** | -2,90* | -3,65 | -2,13 |  | 0,81 | -2,41 | 4,10 |  | -3,52* | -5,14 | -1,97 |  | -5,16* | -8,44 | -1,76 |  | 0,71 | 0,00 | 1,43 |  | 0,61 | -0,98 | 2,17 |
|  | **50** | -2,18* | -2,69 | -1,67 |  | -2,06* | -3,87 | -0,32 |  | -2,18* | -3,64 | -0,71 |  | -5,69* | -8,60 | -2,80 |  | 1,93* | 1,07 | 2,81 |  | 1,64* | 0,11 | 3,11 |
|  | **55** | -0,638 | -1,22 | -0,04 |  | 0,59 | -1,08 | 2,29 |  | 0,11 | -0,87 | 1,10 |  | 1,04 | -2,43 | 4,49 |  | 1,86* | 1,14 | 2,58 |  | 3,00* | 1,39 | 4,58 |
|  | **60** | 0,20 | -0,38 | 0,76 |  | 1,30 | -0,04 | 2,67 |  | 1,10* | 0,09 | 2,11 |  | 3,89* | 0,16 | 7,61 |  | 3,31* | 2,50 | 4,10 |  | 2,46* | 0,84 | 4,07 |
|  | **65** | 0,93* | 0,32 | 1,54 |  | 0,54 | -1,91 | 2,90 |  | 2,07* | 0,60 | 3,51 |  | 0,14 | -2,49 | 2,69 |  | 3,61* | 2,88 | 4,30 |  | 1,06 | -0,98 | 3,11 |
|  | **70** | 0,56* | 0,17 | 0,96 |  | 0,71 | -0,18 | 1,61 |  | 1,95* | 0,33 | 3,60 |  | -0,27 | -4,11 | 3,48 |  | 2,87* | 1,48 | 4,21 |  | 1,92* | 0,64 | 3,22 |
|  | **75** | -0,25 | -1,05 | 0,53 |  | -0,38 | -1,58 | 0,83 |  | 2,17 | -0,21 | 4,60 |  | 0,35 | -4,50 | 5,25 |  | 3,13* | 2,06 | 4,21 |  | 2,26* | 0,62 | 3,92 |
|  | **80** | -0,63 | -1,41 | 0,16 |  | -0,30 | -1,11 | 0,52 |  | 1,51 | -0,41 | 3,40 |  | 1,11 | -1,89 | 4,21 |  | 3,01* | 1,67 | 4,33 |  | 5,07* | 1,99 | 8,24 |
|  |  |  |  |  |  |  |  |  |  |  |  |  |  |  |  |  |  |  |  |  |  |  |  |  |
| **Oral Cavity Cancer** | **40** | -4,09* | -4,67 | -3,58 |  | 1,10 | -1,19 | 3,43 |  | -6,02* | -7,99 | -4,01 |  | -0,97 | -5,70 | 3,73 |  | -1,83* | -2,61 | -1,04 |  | 3,95* | 1,59 | 6,37 |
|  | **45** | -3,84* | -4,58 | -3,10 |  | 0,70 | -1,20 | 2,56 |  | -3,71* | -5,34 | -2,05 |  | -1,76 | -3,71 | 0,17 |  | -0,76* | -1,43 | -0,07 |  | -0,17 | -1,78 | 1,47 |
|  | **50** | -2,73* | -3,08 | -2,38 |  | -0,02 | -1,31 | 1,15 |  | -2,47* | -3,59 | -1,38 |  | -1,67 | -4,18 | 0,79 |  | 0,53 | -0,31 | 1,38 |  | 1,76* | 0,47 | 3,07 |
|  | **55** | -0,98* | -1,61 | -0,34 |  | -0,26 | -1,68 | 1,18 |  | -0,99 | -2,32 | 0,36 |  | -2,52* | -4,78 | -0,26 |  | 2,01* | 1,19 | 2,84 |  | 2,53* | 1,47 | 3,61 |
|  | **60** | -0,41 | -0,86 | 0,04 |  | 0,67 | -0,01 | 1,32 |  | 0,06 | -0,78 | 0,90 |  | -2,60* | -4,69 | -0,47 |  | 2,30* | 1,78 | 2,83 |  | 1,84* | 0,65 | 3,05 |
|  | **65** | 0,20 | -0,37 | 0,78 |  | -0,84* | -1,68 | -0,04 |  | -0,48 | -1,46 | 0,49 |  | -2,12 | -5,80 | 1,71 |  | 2,49* | 1,99 | 2,95 |  | 0,47 | -1,09 | 2,06 |
|  | **70** | 0,19 | -0,42 | 0,78 |  | -0,27 | -0,99 | 0,46 |  | 0,62 | -1,09 | 2,30 |  | 0,03 | -2,34 | 2,46 |  | 3,02* | 1,85 | 4,17 |  | -0,17 | -1,44 | 1,12 |
|  | **75** | -0,14 | -0,70 | 0,40 |  | 0,02 | -1,01 | 1,06 |  | 0,21 | -2,64 | 3,14 |  | -0,88 | -3,66 | 1,97 |  | 2,19* | 1,12 | 3,28 |  | 0,36 | -1,01 | 1,74 |
|  | **80** | 0,06 | -0,39 | 0,50 |  | 0,46 | -0,06 | 0,99 |  | -0,48 | -2,79 | 1,78 |  | -1,45 | -2,99 | 0,06 |  | 2,86* | 1,74 | 3,99 |  | 1,28 | -0,26 | 2,86 |

*APC statistically significant at p < 0.05.

Green indicates negative AAPC (decreasing trend), orange indicates positive AAPC (increasing trend), and white indicates non-significant results (p > 0.05)

**Figure 2.** Age-specific mortality rates (per 100,000 persons) from laryngeal, oropharyngeal cancer and oral cavity cancer by age, period, and birth cohort in Brazil, 1980–2023.

**
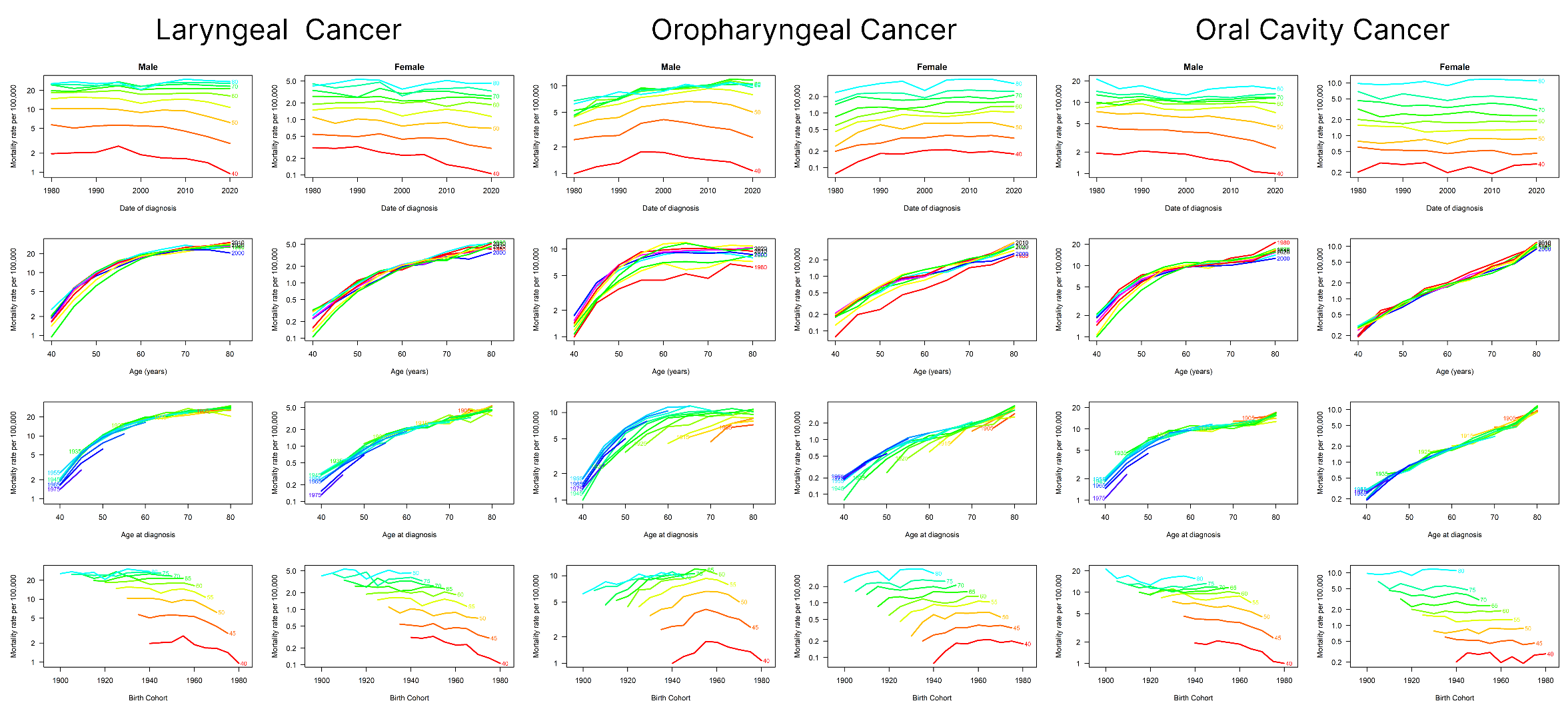
**

Figure layout by row – (1) Age group × period· (2) Period × age group· (3) Birth cohort × age group· (4) Age group × birth cohort·

**Figure 3.** Age-specific mortality rates (per 100,000 persons) from laryngeal, oropharyngeal cancer and oral cavity cancer by age, period, and birth cohort in Brazilian regions (Southeast), 1980–2023.

**
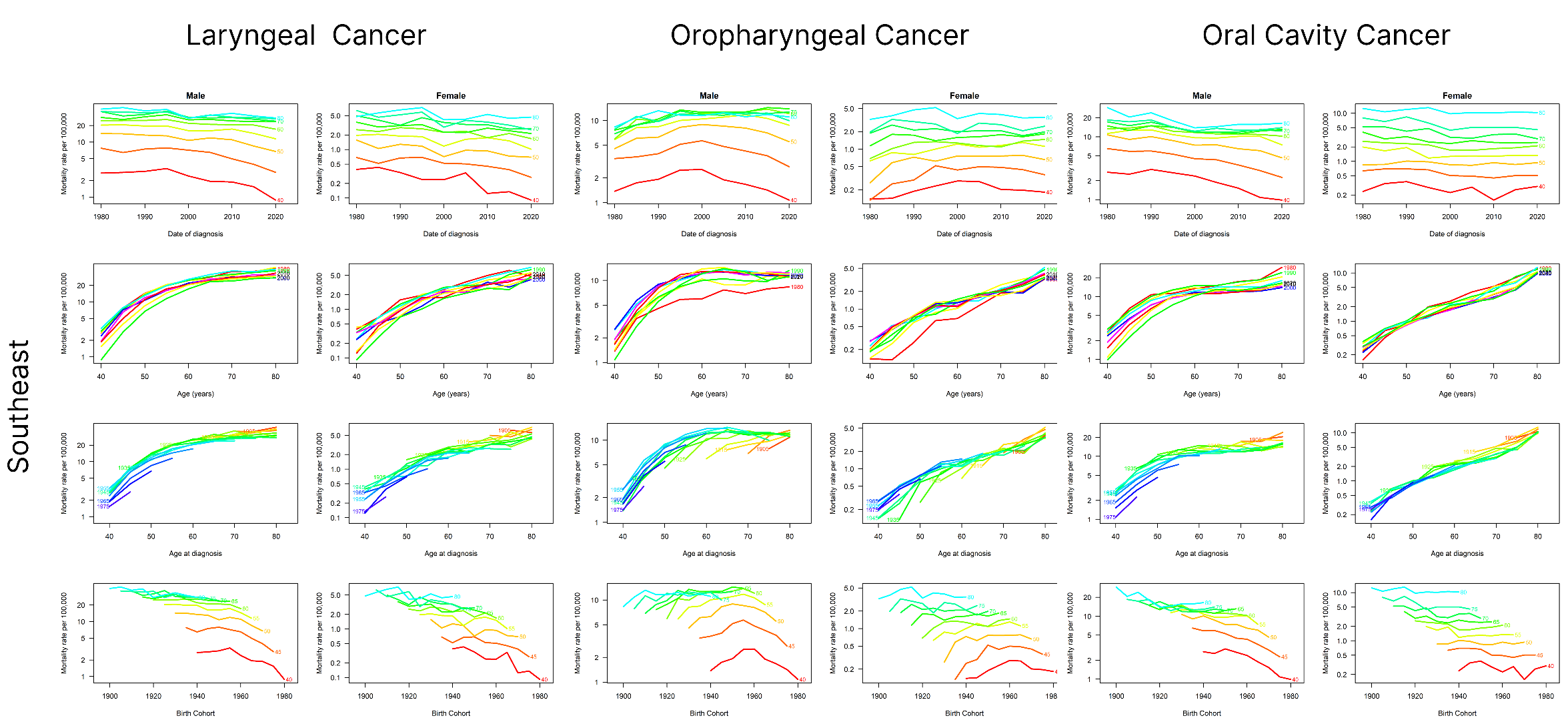
**

Figure layout by row – (1) Age group × period· (2) Period × age group· (3) Birth cohort × age group· (4) Age group × birth cohort·

**Figure 4.** Age-specific mortality rates (per 100,000 persons) from laryngeal, oropharyngeal cancer and oral cavity cancer by age, period, and birth cohort in Brazilian regions (South), 1980–2023.


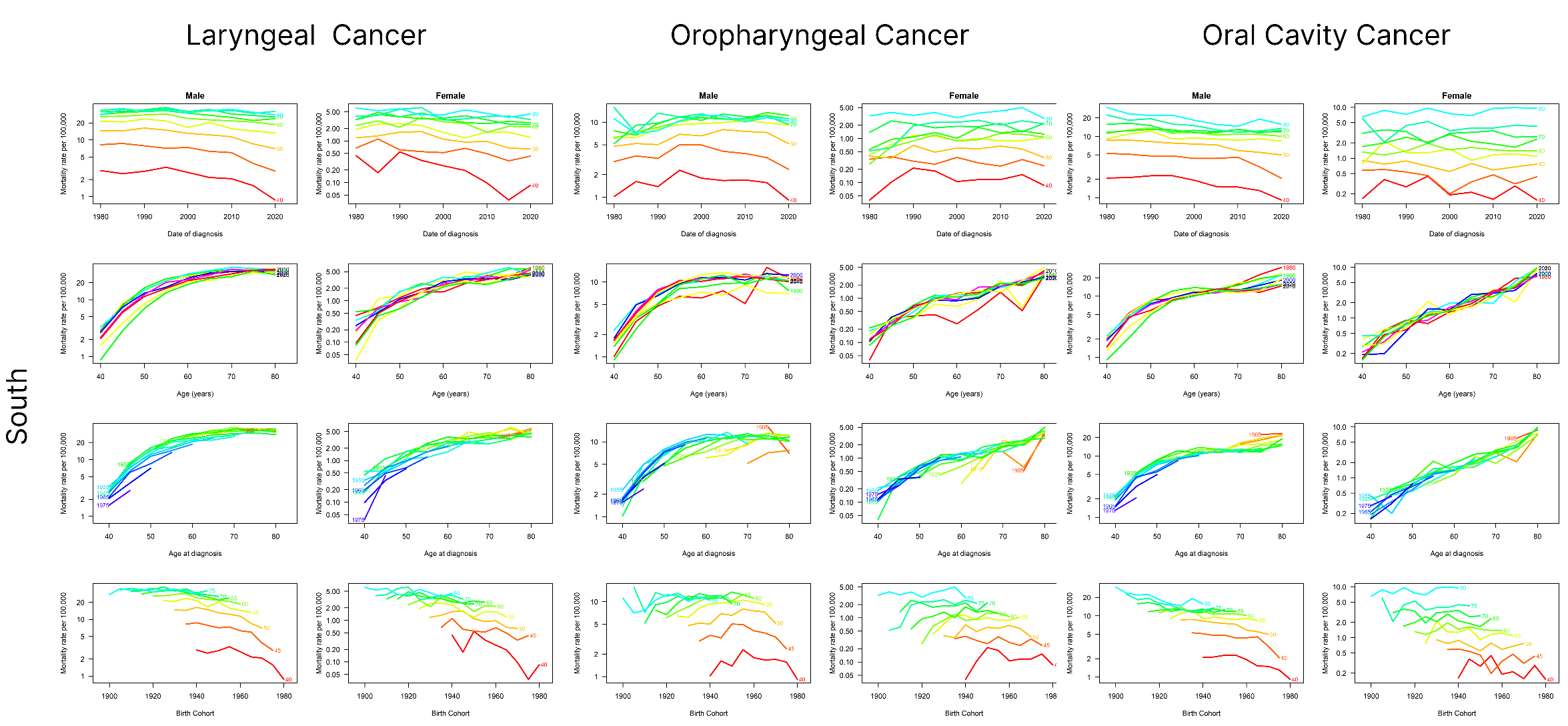


Figure layout by row – (1) Age group × period· (2) Period × age group· (3) Birth cohort × age group· (4) Age group × birth cohort·

**Figure 5.** Age-specific mortality rates (per 100,000 persons) from laryngeal, oropharyngeal cancer and oral cavity cancer by age, period, and birth cohort in Brazilian regions (Mid-west), 1980–2023.


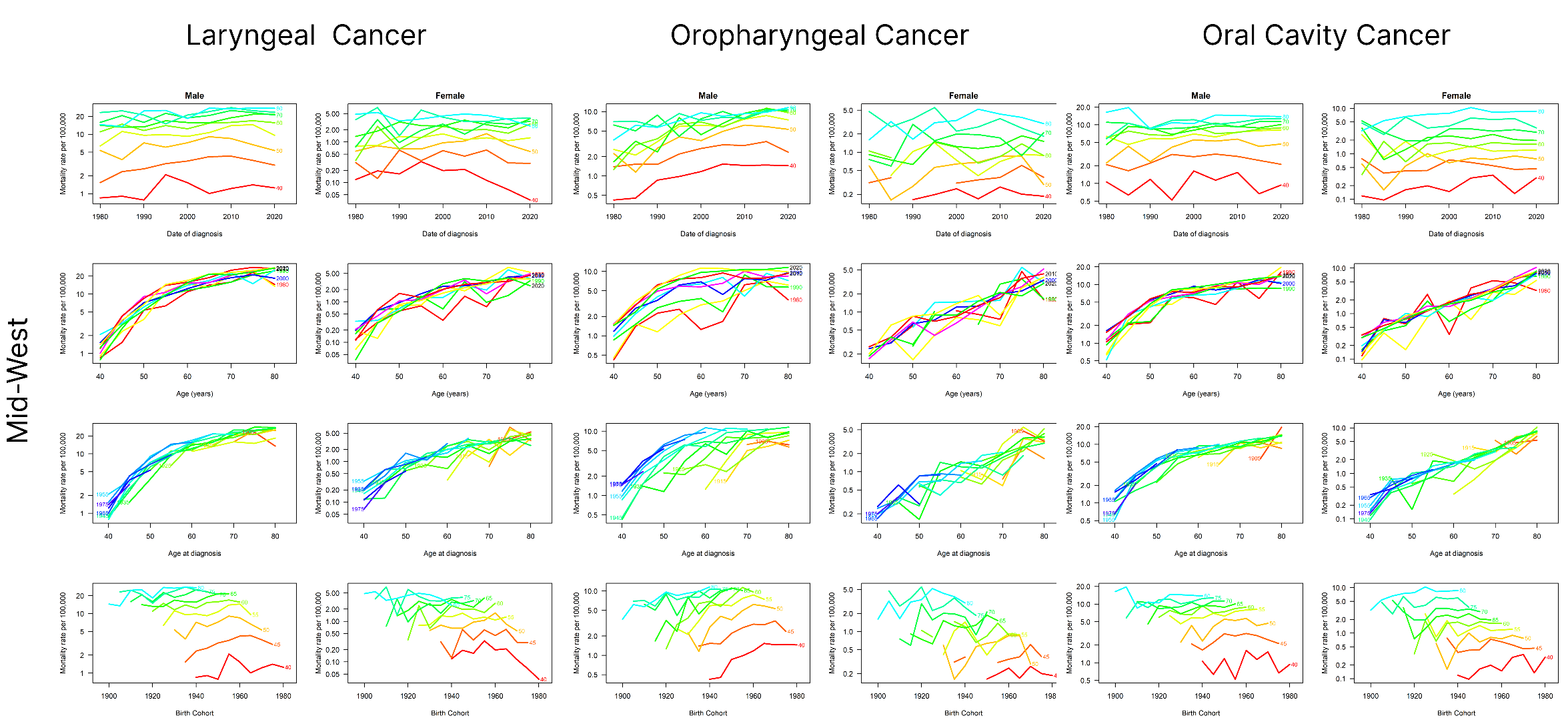


Figure layout by row – (1) Age group × period· (2) Period × age group· (3) Birth cohort × age group· (4) Age group × birth cohort·

**Figure 6.** Age-specific mortality rates (per 100,000 persons) from laryngeal, oropharyngeal cancer and oral cavity cancer by age, period, and birth cohort in Brazilian regions (Northeast), 1980–2023.


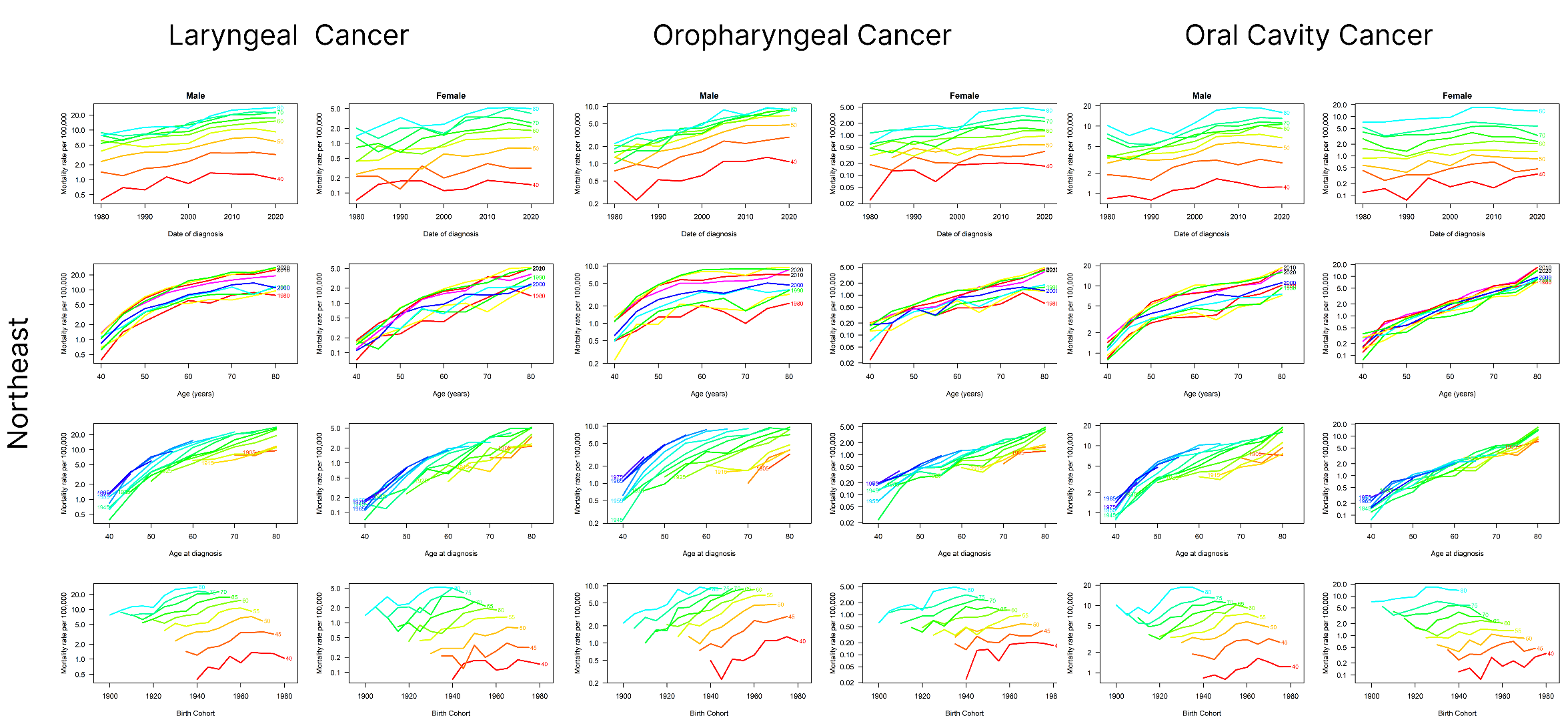


Figure layout by row – (1) Age group × period· (2) Period × age group· (3) Birth cohort × age group· (4) Age group × birth cohort·

**Figure 7.** Age-specific mortality rates (per 100,000 persons) from laryngeal, oropharyngeal cancer and oral cavity cancer by age, period, and birth cohort in Brazilian regions (North), 1980–2023.


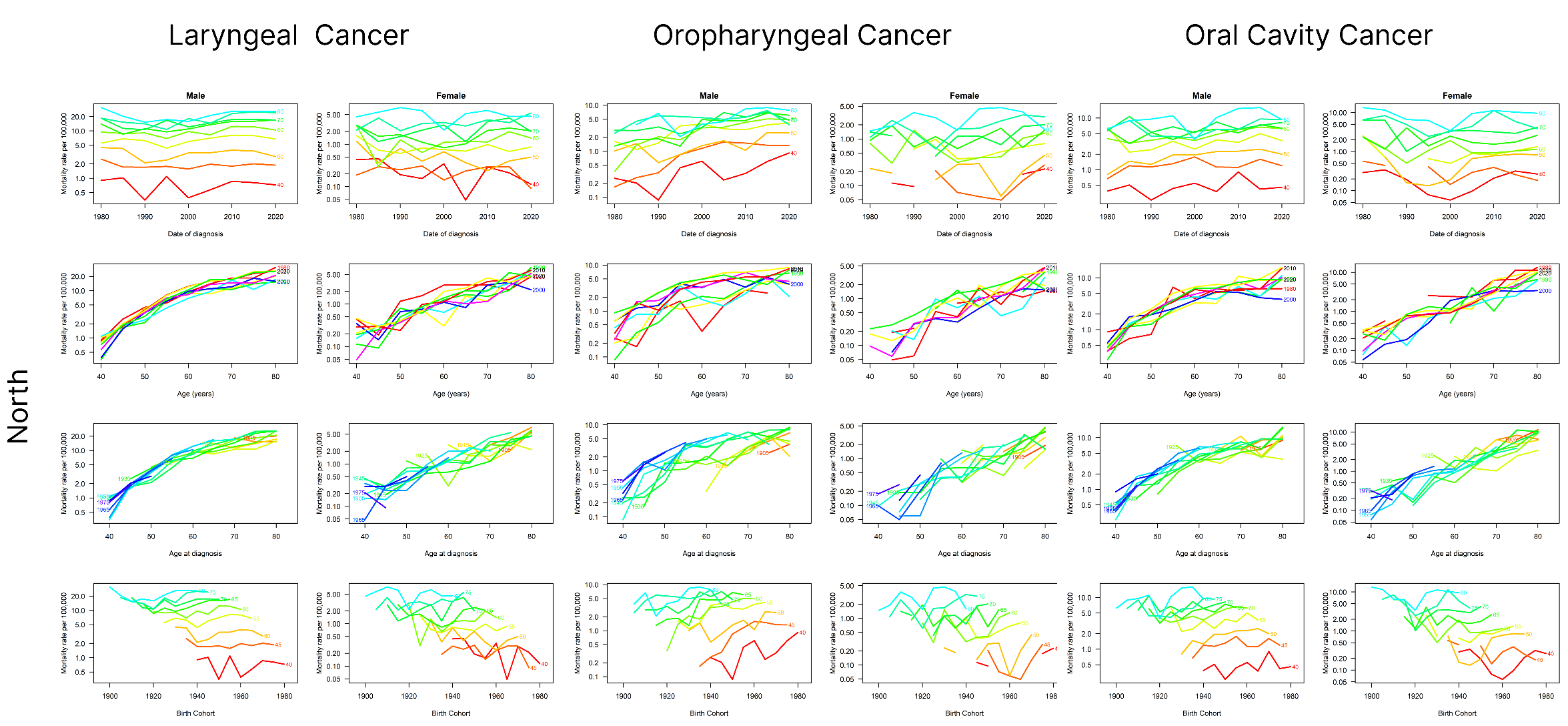


Figure layout by row – (1) Age group × period· (2) Period × age group· (3) Birth cohort × age group· (4) Age group × birth cohort·

**Figure 8.** Age-specific mortality rates (per 100,000 persons) from laryngeal, oropharyngeal cancer and oral cavity cancer by age, period, and birth cohort by sex and ethnicity (males) in Brazil, 2000–2023.


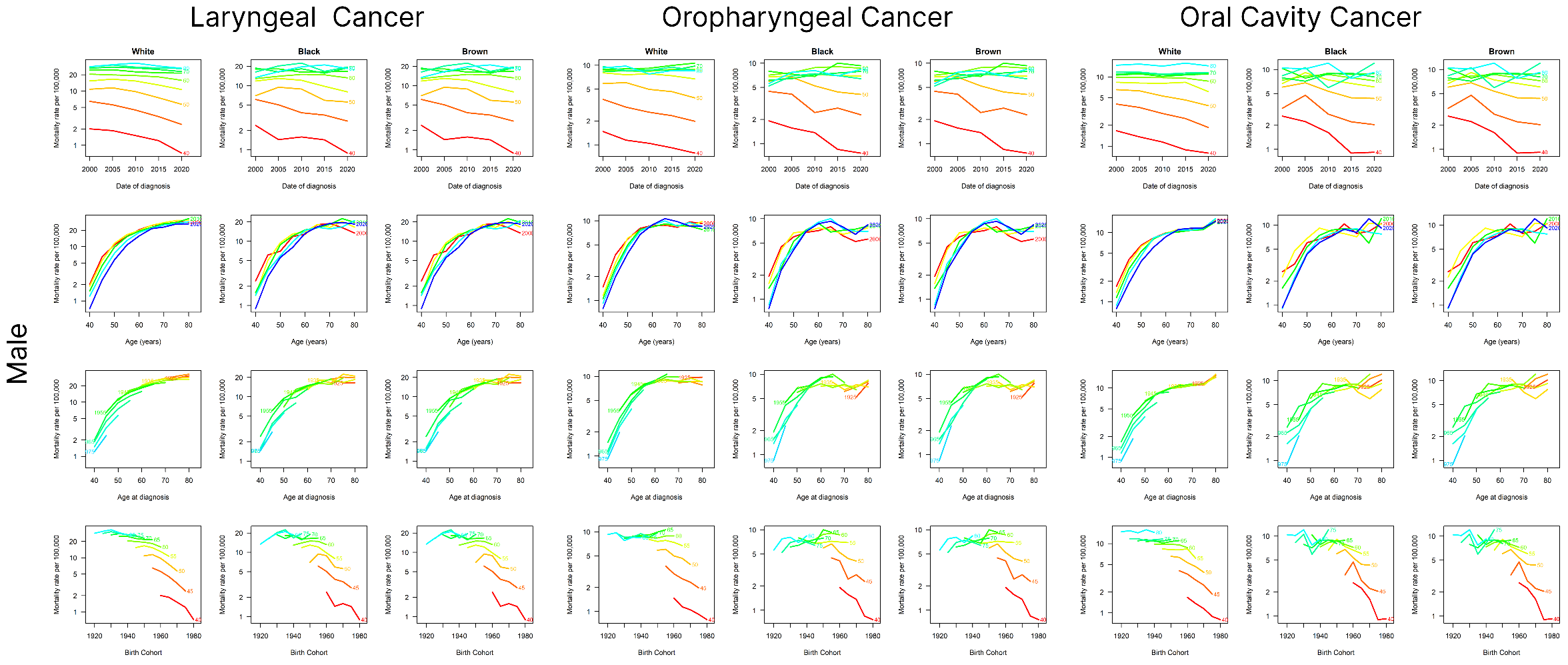


Figure layout by row – (1) Age group × period· (2) Period × age group· (3) Birth cohort × age group· (4) Age group × birth cohort·

**Figure 9.** Age-specific mortality rates (per 100,000 persons) from laryngeal, oropharyngeal cancer and oral cavity cancer by age, period, and birth cohort by sex and ethnicity (females) in Brazil, 2000–2023.


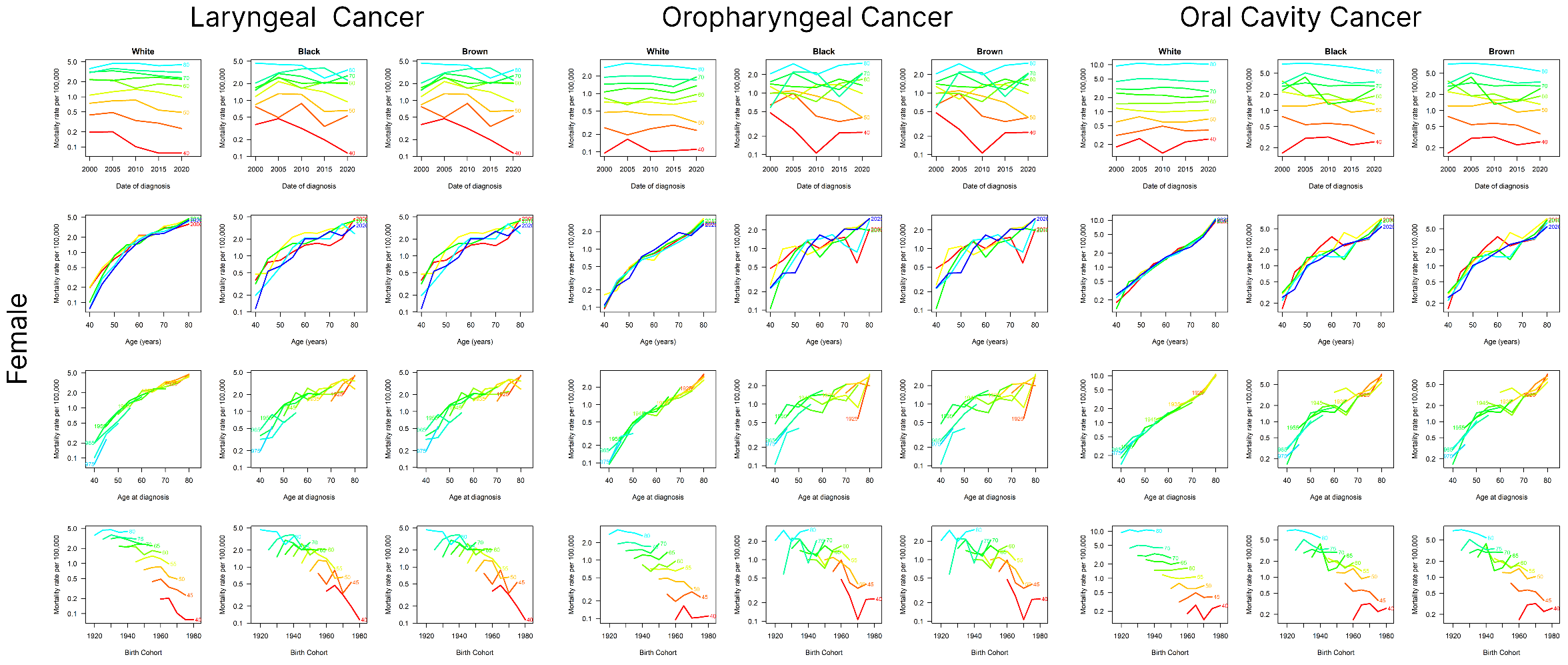


Figure layout by row – (1) Age group × period· (2) Period × age group· (3) Birth cohort × age group· (4) Age group × birth cohort·

**Table 11.** Age–Period–Cohort models of mortality from laryngeal, oropharyngeal, and oral cavity cancer by gender in Brazil and Brazilian regions (1980–2023) and by ethnicity (2000-2023).

| **Brazil** | | | | | | | | | | | | | | | | | | | | | | | | | | | | |
| --- | --- | --- | --- | --- | --- | --- | --- | --- | --- | --- | --- | --- | --- | --- | --- | --- | --- | --- | --- | --- | --- | --- | --- | --- | --- | --- | --- | --- |
| **Laryngeal Cancer** | | | | | | | | |  | **Oropharyngeal Cancer** | | | | | | | | |  | **Oral Cavity Cancer** | | | | | | | | |
| **Model** |  | **Male** | | |  | **Female** | | |  | **Model** |  | **Male** | | |  | **Female** | | |  | **Model** |  | **Male** | | |  | **Female** | | |
|  | **AIC** | **Resid df** | **Deviance** | **p** | **AIC** | **Resid df** | **Deviance** | **p** |  |  | **AIC** | **Resid df** | **Deviance** | **p** | **AIC** | **Resid df** | **Deviance** | **p** |  |  | **AIC** | **Resid df** | **Deviance** | **p** | **AIC** | **Resid df** | **Deviance** | **p** |
| Age | 2355 | 72 | 1609 |  | 868 | 72 | 283 |  |  | Age | 2834 | 72 | 2150 |  | 838 | 72 | 291 |  |  | Age | 1655 | 72 | 950 |  | 790 | 72 | 191 |  |
| Age-drift | 2093 | 71 | 1346 | <0·001 | 818 | 71 | 231 | <0·001 |  | Age-drift | 1801 | 71 | 1114 | <0·001 | 704 | 71 | 154 | <0·001 |  | Age-drift | 1478 | 71 | 771 | <0·001 | 790 | 71 | 189 | 0·28 |
| Age-Cohort | 1039 | 56 | 262 | <0·001 | 748 | 56 | 131 | <0·001 |  | Age-Cohort | 1050 | 56 | 334 | <0·001 | 699 | 56 | 119 | 0·002 |  | Age-Cohort | 932 | 56 | 195 | <0·001 | 771 | 56 | 140 | <0·001 |
| Age-Period-Cohort | 891 | 49 | 100 | <0·001 | 705 | 49 | 73 | <0·001 |  | Age-Period-Cohort | 788 | 49 | 58 | <0·001 | 653 | 49 | 60 | <0·001 |  | Age-Period-Cohort | 815 | 49 | 64 | <0·001 | 729 | 49 | 84 | <0·001 |
| Age-Period | 1830 | 64 | 1069 | <0·001 | 769 | 64 | 167 | <0·001 |  | Age-Period | 1233 | 64 | 533 | <0·001 | 565 | 64 | 92 | 0·006 |  | Age-Period | 1425 | 64 | 704 | <0·001 | 746 | 64 | 131 | 0·001 |
| Age-drift | 2093 | 71 | 1346 | <0·001 | 818 | 71 | 231 | <0·001 |  | Age-drift | 1801 | 71 | 1114 | <0·001 | 704 | 71 | 154 | <0·001 |  | Age-drift | 1478 | 71 | 771 | <0·001 | 790 | 71 | 189 | <0·001 |
| **Southeast** | | | | | | | | | | | | | | | | | | | | | | | | | | | | |
| **Laryngeal Cancer** | | | | | | | | |  | **Oropharyngeal Cancer** | | | | | | | | |  | **Oral Cavity Cancer** | | | | | | | | |
| **Model** |  | **Male** | | |  | **Female** | | |  | **Model** |  | **Male** | | |  | **Female** | | |  | **Model** |  | **Male** | | |  | **Female** | | |
|  | **AIC** | **Resid df** | **Deviance** | **p** | **AIC** | **Resid df** | **Deviance** | **p** |  |  | **AIC** | **Resid df** | **Deviance** | **p** | **AIC** | **Resid df** | **Deviance** | **p** |  |  | **AIC** | **Resid df** | **Deviance** | **p** | **AIC** | **Resid df** | **Deviance** | **p** |
| Age | 2784 | 72 | 2086 |  | 967 | 72 | 434 |  |  | Age | 1693 | 72 | 1051 |  | 659 | 72 | 161 |  |  | Age | 2113 | 72 | 1457 |  | 780 | 72 | 236 |  |
| Age-drift | 1485 | 71 | 785 | <0·001 | 732 | 71 | 199 | <0·001 |  | Age-drift | 1578 | 71 | 934 | <0·001 | 651 | 71 | 151 | 0·001 |  | Age-drift | 1247 | 71 | 590 | <0·001 | 689 | 71 | 142 | <0·001 |
| Age-Cohort | 935 | 56 | 205 | <0·001 | 697 | 56 | 132 | 0·001 |  | Age-Cohort | 995 | 56 | 322 | <0·001 | 633 | 56 | 103 | <0·001 |  | Age-Cohort | 905 | 56 | 218 | <0·001 | 681 | 56 | 105 | 0·001 |
| Age-Period-Cohort | 817 | 49 | 73 | <0·001 | 660 | 49 | 81 | 0·001 |  | Age-Period-Cohort | 731 | 49 | 43 | <0·001 | 590 | 49 | 46 | <0·001 |  | Age-Period-Cohort | 762 | 49 | 61 | <0·001 | 650 | 49 | 60 | <0·001 |
| Age-Period | 1279 | 64 | 565 | <0·001 | 690 | 64 | 141 | 0·001 |  | Age-Period | 1072 | 64 | 415 | <0·001 | 624 | 64 | 109 | <0·001 |  | Age-Period | 1178 | 64 | 507 | <0·001 | 644 | 64 | 84 | 0·07 |
| Age-drift | 1485 | 71 | 785 | <0·001 | 734 | 71 | 199 | <0·001 |  | Age-drift | 1578 | 71 | 934 | <0·001 | 651 | 71 | 151 | <0·001 |  | Age-drift | 1247 | 71 | 590 | <0·001 | 689 | 71 | 142 | <0·001 |
| **South** | | | | | | | | | | | | | | | | | | | | | | | | | | | | |
| **Laryngeal Cancer** | | | | | | | | |  | **Oropharyngeal Cancer** | | | | | | | | |  | **Oral Cavity Cancer** | | | | | | | | |
| **Model** |  | **Male** | | |  | **Female** | | |  | **Model** |  | **Male** | | |  | **Female** | | |  | **Model** |  | **Male** | | |  | **Female** | | |
|  | **AIC** | **Resid df** | **Deviance** | **p** | **AIC** | **Resid df** | **Deviance** | **p** |  |  | **AIC** | **Resid df** | **Deviance** | **p** | **AIC** | **Resid df** | **Deviance** | **p** |  |  | **AIC** | **Resid df** | **Deviance** | **p** | **AIC** | **Resid df** | **Deviance** | **p** |
| Age | 1473 | 72 | 856 |  | 655 | 72 | 207 |  |  | Age | 902 | 72 | 353 |  | 481 | 72 | 90 |  |  | Age | 891 | 72 | 323 |  | 534 | 72 | 99 |  |
| Age-drift | 976 | 71 | 357 | <0·001 | 570 | 71 | 120 | <0·001 |  | Age-drift | 847 | 71 | 296 | <0·001 | 475 | 71 | 82 | 0·005 |  | Age-drift | 731 | 71 | 161 | <0·001 | 536 | 71 | 98 | 0·57 |
| Age-Cohort | 747 | 56 | 98 | <0·001 | 564 | 56 | 83 | 0·001 |  | Age-Cohort | 724 | 56 | 143 | <0·001 | 489 | 56 | 66 | 0·36 |  | Age-Cohort | 677 | 56 | 78 | <0·001 | 527 | 56 | 59 | <0·001 |
| Age-Period-Cohort | 723 | 49 | 60 | <0·001 | 557 | 49 | 62 | 0·004 |  | Age-Period-Cohort | 655 | 49 | 60 | <0·001 | 484 | 49 | 47 | 0·007 |  | Age-Period-Cohort | 743 | 49 | 59 | 0·010 | 535 | 49 | 54 | 0·63 |
| Age-Period | 884 | 64 | 251 | <0·001 | 563 | 64 | 98 | 0·001 |  | Age-Period | 724 | 64 | 159 | <0·001 | 467 | 64 | 60 | 0·57 |  | Age-Period | 734 | 64 | 150 | <0·001 | 544 | 64 | 92 | <0·001 |
| Age-drift | 976 | 71 | 357 | <0·001 | 570 | 71 | 120 | 0·003 |  | Age-drift | 847 | 71 | 293 | <0·001 | 475 | 71 | 82 | 0·002 |  | Age-drift | 731 | 71 | 161 | 0·13 | 536 | 71 | 98 | 0·54 |
| **Mid-west** | | | | | | | | | | | | | | | | | | | | | | | | | | | | |
| **Laryngeal Cancer** | | | | | | | | |  | **Oropharyngeal Cancer** | | | | | | | | |  | **Oral Cavity Cancer** | | | | | | | | |
| **Model** |  | **Male** | | |  | **Female** | | |  | **Model** |  | **Male** | | |  | **Female** | | |  | **Model** |  | **Male** | | |  | **Female** | | |
|  | **AIC** | **Resid df** | **Deviance** | **p** | **AIC** | **Resid df** | **Deviance** | **p** |  |  | **AIC** | **Resid df** | **Deviance** | **p** | **AIC** | **Resid df** | **Deviance** | **p** |  |  | **AIC** | **Resid df** | **Deviance** | **p** | **AIC** | **Resid df** | **Deviance** | **p** |
| Age | 706 | 72 | 209 |  | 422 | 72 | 86 |  |  | Age | 806 | 72 | 371 |  | 393 | 72 | 97 |  |  | Age | 571 | 72 | 115 |  | 406 | 72 | 59 |  |
| Age-drift | 650 | 71 | 151 | <0·001 | 423 | 71 | 84 | 0·17 |  | Age-drift | 558 | 71 | 121 | <0·001 | 390 | 71 | 92 | 0·035 |  | Age-drift | 549 | 71 | 91 | <0·001 | 407 | 71 | 57 | 0·23 |
| Age-Cohort | 617 | 56 | 87 | <0·001 | 425 | 56 | 56 | 0·023 |  | Age-Cohort | 552 | 56 | 84 | 0·001 | 404 | 56 | 76 | 0·37 |  | Age-Cohort | 549 | 56 | 61 | 0·011 | 426 | 56 | 47 | 0·80 |
| Age-Period-Cohort | 599 | 49 | 55 | <0·001 | 423 | 49 | 41 | 0·027 |  | Age-Period-Cohort | 530 | 49 | 49 | <0·001 | 405 | 49 | 63 | 0·07 |  | Age-Period-Cohort | 553 | 49 | 51 | 0·196 | 427 | 49 | 34 | 0·07 |
| Age-Period | 620 | 64 | 106 | <0·001 | 422 | 64 | 69 | 0·018 |  | Age-Period | 532 | 64 | 81 | 0·007 | 392 | 64 | 80 | 0·30 |  | Age-Period | 552 | 64 | 80 | 0·017 | 409 | 64 | 46 | 0·71 |
| Age-drift | 650 | 71 | 151 | <0·001 | 423 | 71 | 84 | 0·036 |  | Age-drift | 558 | 71 | 121 | <0·001 | 390 | 71 | 92 | 0·10 |  | Age-drift | 549 | 71 | 91 | 0·119 | 407 | 71 | 57 | 0·10 |
| **Northeast** | | | | | | | | | | | | | | | | | | | | | | | | | | | | |
| **Laryngeal Cancer** | | | | | | | | |  | **Oropharyngeal Cancer** | | | | | | | | |  | **Oral Cavity Cancer** | | | | | | | | |
| **Model** |  | **Male** | | |  | **Female** | | |  | **Model** |  | **Male** | | |  | **Female** | | |  | **Model** |  | **Male** | | |  | **Female** | | |
|  | **AIC** | **Resid df** | **Deviance** | **p** | **AIC** | **Resid df** | **Deviance** | **p** |  |  | **AIC** | **Resid df** | **Deviance** | **p** | **AIC** | **Resid df** | **Deviance** | **p** |  |  | **AIC** | **Resid df** | **Deviance** | **p** | **AIC** | **Resid df** | **Deviance** | **p** |
| Age | 3016 | 72 | 2432 |  | 899 | 72 | 462 |  |  | Age | 2456 | 72 | 1936 |  | 797 | 72 | 386 |  |  | Age | 1716 | 72 | 1151 |  | 894 | 72 | 409 |  |
| Age-drift | 910 | 71 | 324 | <0·001 | 566 | 71 | 127 | <0·001 |  | Age-drift | 693 | 71 | 170 | <0·001 | 521 | 71 | 108 | <0·001 |  | Age-drift | 854 | 71 | 286 | <0·001 | 748 | 71 | 261 | <0·001 |
| Age-Cohort | 802 | 56 | 486 | <0·001 | 568 | 56 | 99 | 0·019 |  | Age-Cohort | 661 | 56 | 109 | <0·001 | 817 | 56 | 74 | 0·003 |  | Age-Cohort | 734 | 56 | 137 | <0·001 | 714 | 56 | 197 | <0·001 |
| Age-Period-Cohort | 696 | 49 | 66 | <0·001 | 547 | 49 | 64 | <0·001 |  | Age-Period-Cohort | 624 | 49 | 57 | <0·001 | 515 | 49 | 59 | 0·032 |  | Age-Period-Cohort | 663 | 49 | 52 | <0·001 | 590 | 49 | 59 | <0·001 |
| Age-Period | 746 | 64 | 147 | <0·001 | 535 | 64 | 82 | 0·27 |  | Age-Period | 629 | 64 | 93 | 0·002 | 508 | 64 | 81 | 0·10 |  | Age-Period | 722 | 64 | 141 | <0·001 | 591 | 64 | 90 | 0·007 |
| Age-drift | 910 | 71 | 324 | <0·001 | 566 | 71 | 127 | <0·001 |  | Age-drift | 693 | 71 | 170 | <0·001 | 521 | 71 | 108 | <0·001 |  | Age-drift | 854 | 71 | 286 | <0·001 | 478 | 71 | 261 | <0·001 |
| **North** | | | | | | | | | | | | | | | | | | | | | | | | | | | | |
| **Laryngeal Cancer** | | | | | | | | |  | **Oropharyngeal Cancer** | | | | | | | | |  | **Oral Cavity Cancer** | | | | | | | | |
| **Model** |  | **Male** | | |  | **Female** | | |  | **Model** |  | **Male** | | |  | **Female** | | |  | **Model** |  | **Male** | | |  | **Female** | | |
|  | **AIC** | **Resid df** | **Deviance** | **p** | **AIC** | **Resid df** | **Deviance** | **p** |  |  | **AIC** | **Resid df** | **Deviance** | **p** | **AIC** | **Resid df** | **Deviance** | **p** |  |  | **AIC** | **Resid df** | **Deviance** | **p** | **AIC** | **Resid df** | **Deviance** | **p** |
| Age | 610 | 72 | 150 |  | 394 | 72 | 81 |  |  | Age | 615 | 72 | 249 |  | 350 | 72 | 105 |  |  | Age | 542 | 72 | 138 |  | 461 | 72 | 141 |  |
| Age-drift | 569 | 71 | 106 | <0·001 | 395 | 71 | 80 | 0·53 |  | Age-drift | 478 | 71 | 109 | <0·001 | 336 | 71 | 89 | <0·001 |  | Age-drift | 512 | 71 | 106 | <0·001 | 462 | 71 | 139 | 0·21 |
| Age-Cohort | 573 | 56 | 81 | 0·042 | 404 | 56 | 59 | 0·15 |  | Age-Cohort | 493 | 56 | 95 | 0·48 | 347 | 56 | 70 | 0·22 |  | Age-Cohort | 514 | 56 | 78 | 0·024 | 450 | 56 | 98 | <0·001 |
| Age-Period-Cohort | 543 | 49 | 37 | <0·001 | 399 | 49 | 40 | 0·007 |  | Age-Period-Cohort | 486 | 49 | 74 | 0·004 | 357 | 49 | 66 | 0·80 |  | Age-Period-Cohort | 511 | 49 | 61 | 0·015 | 435 | 49 | 69 | <0·001 |
| Age-Period | 536 | 64 | 60 | 0·08 | 393 | 64 | 64 | 0·08 |  | Age-Period | 474 | 64 | 92 | 0·27 | 344 | 64 | 84 | 0·31 |  | Age-Period | 503 | 64 | 84 | 0·09 | 436 | 64 | 100 | 0·008 |
| Age-drift | 569 | 71 | 106 | <0·001 | 395 | 71 | 80 | 0·021 |  | Age-drift | 478 | 71 | 109 | 0·013 | 336 | 71 | 89 | 0·60 |  | Age-drift | 512 | 71 | 106 | 0·002 | 462 | 71 | 139 | <0·001 |
| **White** | | | | | | | | | | | | | | | | | | | | | | | | | | | | |
| **Laryngeal Cancer** | | | | | | | | |  | **Oropharyngeal Cancer** | | | | | | | | |  | **Oral Cavity Cancer** | | | | | | | | |
| **Model** |  | **Male** | | |  | **Female** | | |  | **Model** |  | **Male** | | |  | **Female** | | |  | **Model** |  | **Male** | | |  | **Female** | | |
|  | **AIC** | **Resid df** | **Deviance** | **p** | **AIC** | **Resid df** | **Deviance** | **p** |  |  | **AIC** | **Resid df** | **Deviance** | **p** | **AIC** | **Resid df** | **Deviance** | **p** |  |  | **AIC** | **Resid df** | **Deviance** | **p** | **AIC** | **Resid df** | **Deviance** | **p** |
| Age | 1407 | 36 | 1002 |  | 423 | 36 | 112 |  |  | Age | 599 | 36 | 228 |  | 329 | 36 | 40 |  |  | Age | 735 | 36 | 357 |  | 363 | 36 | 44 |  |
| Age-drift | 873 | 35 | 466 | <0·001 | 404 | 35 | 91 | <0·001 |  | Age-drift | 565 | 35 | 193 | <0·001 | 331 | 35 | 40 | 0·92 |  | Age-drift | 622 | 35 | 242 | <0·001 | 365 | 35 | 44 | 0·98 |
| Age-Cohort | 475 | 24 | 46 | <0·001 | 371 | 24 | 36 | <0·001 |  | Age-Cohort | 426 | 24 | 32 | <0·001 | 342 | 24 | 29 | 0·42 |  | Age-Cohort | 425 | 24 | 23 | <0·001 | 371 | 24 | 28 | 0·17 |
| Age-Period-Cohort | 459 | 21 | 24 | <0·001 | 372 | 21 | 31 | 0·19 |  | Age-Period-Cohort | 418 | 21 | 17 | 0·002 | 345 | 21 | 26 | 0·34 |  | Age-Period-Cohort | 424 | 21 | 16 | 0·06 | 375 | 21 | 26 | 0·56 |
| Age-Period | 812 | 32 | 399 | <0·001 | 398 | 32 | 79 | <0·001 |  | Age-Period | 566 | 32 | 188 | <0·001 | 333 | 32 | 36 | 0·46 |  | Age-Period | 619 | 32 | 233 | <0·001 | 367 | 32 | 40 | 0·26 |
| Age-drift | 873 | 35 | 466 | <0·001 | 404 | 35 | 91 | 0·011 |  | Age-drift | 565 | 35 | 193 | 0·20 | 331 | 35 | 40 | 0·28 |  | Age-drift | 622 | 35 | 242 | 0·034 | 365 | 35 | 44 | 0·27 |
| **Black** | | | | | | | | | | | | | | | | | | | | | | | | | | | | |
| **Laryngeal Cancer** | | | | | | | | |  | **Oropharyngeal Cancer** | | | | | | | | |  | **Oral Cavity Cancer** | | | | | | | | |
| **Model** |  | **Male** | | |  | **Female** | | |  | **Model** |  | **Male** | | |  | **Female** | | |  | **Model** |  | **Male** | | |  | **Female** | | |
|  | **AIC** | **Resid df** | **Deviance** | **p** | **AIC** | **Resid df** | **Deviance** | **p** |  |  | **AIC** | **Resid df** | **Deviance** | **p** | **AIC** | **Resid df** | **Deviance** | **p** |  |  | **AIC** | **Resid df** | **Deviance** | **p** | **AIC** | **Resid df** | **Deviance** | **p** |
| Age | 464 | 36 | 149 |  | 291 | 36 | 59 |  |  | Age | 377 | 36 | 90 |  | 273 | 36 | 56 |  |  | Age | 429 | 36 | 136 |  | 295 | 36 | 55 |  |
| Age-drift | 431 | 35 | 114 | <0·001 | 287 | 35 | 53 | 0·011 |  | Age-drift | 375 | 35 | 85 | 0·027 | 275 | 35 | 56 | 0·60 |  | Age-drift | 396 | 35 | 102 | <0·001 | 281 | 35 | 39 | <0·001 |
| Age-Cohort | 363 | 24 | 24 | <0·001 | 293 | 24 | 36 | 0·13 |  | Age-Cohort | 333 | 24 | 21 | <0·001 | 265 | 24 | 24 | <0·001 |  | Age-Cohort | 357 | 24 | 41 | <0·001 | 297 | 24 | 33 | 0·86 |
| Age-Period-Cohort | 365 | 21 | 20 | 0·25 | 283 | 21 | 21 | 0·001 |  | Age-Period-Cohort | 335 | 21 | 17 | 0·28 | 267 | 21 | 19 | 0·24 |  | Age-Period-Cohort | 353 | 21 | 30 | 0·012 | 300 | 21 | 30 | 0·38 |
| Age-Period | 427 | 32 | 104 | <0·001 | 279 | 32 | 38 | 0·10 |  | Age-Period | 379 | 32 | 83 | <0·001 | 278 | 32 | 53 | <0·001 |  | Age-Period | 391 | 32 | 90 | <0·001 | 284 | 32 | 36 | 0·87 |
| Age-drift | 431 | 35 | 114 | 0·017 | 287 | 35 | 53 | 0·002 |  | Age-drift | 375 | 35 | 85 | 0·70 | 275 | 35 | 56 | 0·40 |  | Age-drift | 396 | 35 | 102 | 0·010 | 281 | 35 | 39 | 0·38 |
| **Brown** | | | | | | | | | | | | | | | | | | | | | | | | | | | | |
| **Laryngeal Cancer** | | | | | | | | |  | **Oropharyngeal Cancer** | | | | | | | | |  | **Oral Cavity Cancer** | | | | | | | | |
| **Model** |  | **Male** | | |  | **Female** | | |  | **Model** |  | **Male** | | |  | **Female** | | |  | **Model** |  | **Male** | | |  | **Female** | | |
|  | **AIC** | **Resid df** | **Deviance** | **p** | **AIC** | **Resid df** | **Deviance** | **p** |  |  | **AIC** | **Resid df** | **Deviance** | **p** | **AIC** | **Resid df** | **Deviance** | **p** |  |  | **AIC** | **Resid df** | **Deviance** | **p** | **AIC** | **Resid df** | **Deviance** | **p** |
| Age | 1019 | 36 | 644 |  | 363 | 36 | 73 |  |  | Age | 683 | 36 | 339 |  | 348 | 36 | 74 |  |  | Age | 619 | 36 | 265 |  | 394 | 36 | 93 |  |
| Age-drift | 694 | 35 | 317 | <0·001 | 340 | 35 | 48 | <0·001 |  | Age-drift | 476 | 35 | 130 | <0·001 | 325 | 35 | 49 | <0·001 |  | Age-drift | 534 | 35 | 178 | <0·001 | 393 | 35 | 90 | 0·09 |
| Age-Cohort | 501 | 24 | 101 | <0·001 | 345 | 24 | 30 | 0·10 |  | Age-Cohort | 423 | 24 | 54 | <0·001 | 319 | 24 | 21 | 0·003 |  | Age-Cohort | 419 | 24 | 41 | <0·001 | 380 | 24 | 55 | <0·001 |
| Age-Period-Cohort | 414 | 21 | 10 | <0·001 | 334 | 21 | 14 | <0·001 |  | Age-Period-Cohort | 403 | 21 | 28 | <0·001 | 319 | 21 | 15 | 0·10 |  | Age-Period-Cohort | 405 | 21 | 23 | <0·001 | 350 | 21 | 20 | <0·001 |
| Age-Period | 545 | 32 | 162 | <0·001 | 323 | 32 | 24 | 0·48 |  | Age-Period | 441 | 32 | 88 | <0·001 | 319 | 32 | 36 | 0·025 |  | Age-Period | 498 | 32 | 136 | <0·001 | 353 | 32 | 44 | 0·009 |
| Age-drift | 694 | 35 | 317 | <0·001 | 340 | 35 | 48 | <0·001 |  | Age-drift | 476 | 35 | 130 | <0·001 | 325 | 35 | 49 | 0·006 |  | Age-drift | 534 | 35 | 178 | <0·001 | 393 | 35 | 90 | <0·001 |

**Table 12.** Mortality rate (per 100,000 persons) and relative risk (95% confidence interval) from laryngeal, oropharyngeal and oral cavity cancer by gender in Southeast region, 1980–2023.

|  |  | **Laryngeal Cancer** | |  | **Oropharyngeal Cancer** | |  | **Oral Cavity Cancer** | |
| --- | --- | --- | --- | --- | --- | --- | --- | --- | --- |
|  | | **Male** | **Female** |  | **Male** | **Female** |  | **Male** | **Female** |
|  |  | **Rate (95%CI)** | **Rate (95%CI)** |  | **Rate (95%CI)** | **Rate (95%CI)** |  | **Rate (95%CI)** | **Rate (95%CI)** |
| **Age Group** | 40 | 2·62 (2·49 - 2·76) | 0·25 (0·21 - 0·29) |  | 2·56 (2·41 - 2·72) | 0·23 (0·19 - 0·28) |  | 2·26 (2·13 - 2·40) | 0·23 (0·21 - 0·26) |
|  | 45 | 6·70 (6·43 - 6·98) | 0·50 (0·44 - 0·57) |  | 5·60 (5·33 - 5·89) | 0·44 (0·38 - 0·51) |  | 4·68 (4·45 - 4·93) | 0·48 (0·43 - 0·53) |
|  | 50 | 12·26 (11·81 - 12·72) | 0·93 (0·83 - 1·04) |  | 9·26 (8·85 - 9·70) | 0·75 (0·65 - 0·86) |  | 7·78 (7·42 - 8·16) | 0·79 (0·72 - 0·86) |
|  | 55 | 18·23 (17·59 - 18·90) | 1·55 (1·39 - 1·73) |  | 12·31 (11·76 - 12·89) | 1·19 (1·05 - 1·35) |  | 10·6 (10·11 - 11·11) | 1·23 (1·13 - 1·34) |
|  | 60 | 23·92 (23·07 - 24·80) | 2·18 (1·96 - 2·42) |  | 14·81 (14·13 - 15·52) | 1·42 (1·25 - 1·61) |  | 12·30 (11·73 - 12·90) | 1·77 (1·63 - 1·92) |
|  | 65 | 29·23 (28·17 - 30·34) | 2·64 (2·37 - 2·95) |  | 16·05 (15·27 - 16·87) | 1·96 (1·73 - 2·23) |  | 13·74 (13·07 - 14·43) | 2·33 (2·15 - 2·52) |
|  | 70 | 33·7 (32·35 - 35·10) | 3·48 (3·10 - 3·91) |  | 15·39 (14·54 - 16·29) | 2·42 (2·11 - 2·78) |  | 14·78 (13·99 - 15·61) | 3·27 (3·02 - 3·54) |
|  | 75 | 36·18 (34·64 - 37·78) | 4·01 (3·56 - 4·52) |  | 15·39 (14·46 - 16·37) | 2·92 (2·54 - 3·36) |  | 16·25 (15·32 - 17·23) | 4·83 (4·47 - 5·22) |
|  | 80 | 39·77 (38·07 - 41·54) | 5·46 (4·86 - 6·12) |  | 15·73 (14·77 - 16·74) | 4·43 (3·89 - 5·03) |  | 19·92 (18·81 - 21·09) | 9·54 (8·91 - 10·21) |
|  |  | **RR (95%CI)** | **RR (95%CI)** |  | **RR (95%CI)** | **RR (95%CI)** |  | **RR (95%CI)** | **RR (95%CI)** |
| **Period** | 1980 | 1·34 (1·29 - 1·40) | 1·66 (1·49 - 1·86) |  | 0·62 (0·58 - 0·66) | 0·57 (0·48 - 0·68) |  | 1·49 (1·42 - 1·57) | 1·47 (1·34 - 1·62) |
|  | 1985 | 1·26 (1·21 - 1·31) | 1·36 (1·23 - 1·52) |  | 0·81 (0·77 - 0·86) | 0·90 (0·79 - 1·04) |  | 1·36 (1·30 - 1·43) | 1·32 (1·20 - 1·45) |
|  | 1990 | 1·24 (1·20 - 1·29) | 1·42 (1·29 - 1·57) |  | 0·84 (0·80 - 0·88) | 0·97 (0·85 - 1·10) |  | 1·44 (1·38 - 1·51) | 1·41 (1·29 - 1·54) |
|  | 1995 | 1·27 (1·23 - 1·31) | 1·47 (1·34 - 1·62) |  | 1·01 (0·97 - 1·06) | 1·05 (0·93 - 1·18) |  | 1·19 (1·14 - 1·25) | 1·27 (1·17 - 1·39) |
|  | 2000 | 1·00 (Reference) | 1·00 (Reference) |  | 1·00 (Reference) | 1·00 (Reference) |  | 1·00 (Reference) | 1·00 (Reference) |
|  | 2005 | 0·99 (0·96 - 1·02) | 1·09 (0·99 - 1·19) |  | 0·95 (0·91 - 0·98) | 1·04 (0·94 - 1·16) |  | 0·96 (0·92 - 1·00) | 1·05 (0·97 - 1·14) |
|  | 2010 | 0·95 (0·92 - 0·98) | 1·05 (0·96 - 1·14) |  | 0·94 (0·90 - 0·98) | 1·00 (0·90 - 1·11) |  | 0·92 (0·88 - 0·96) | 1·06 (0·98 - 1·14) |
|  | 2015 | 0·87 (0·85 - 0·90) | 0·95 (0·87 - 1·04) |  | 0·94 (0·90 - 0·98) | 0·94 (0·85 - 1·04) |  | 0·94 (0·90 - 0·97) | 1·09 (1·01 - 1·18) |
|  | 2020 | 0·79 (0·77 - 0·82) | 0·84 (0·77 - 0·93) |  | 0·86 (0·82 - 0·90) | 0·93 (0·84 - 1·03) |  | 0·90 (0·86 - 0·94) | 1·06 (0·98 - 1·15) |
|  |  | **RR (95%CI)** | **RR (95%CI)** |  | **RR (95%CI)** | **RR (95%CI)** |  | **RR (95%CI)** | **RR (95%CI)** |
| **Birth Cohort** | 1900 | 0·75 (0·66 - 0·85) | 0·53 (0·39 - 0·71) |  | 0·86 (0·66 - 1·12) | 1·29 (0·90 - 1·87) |  | 0·98 (0·85 - 1·14) | 0·89 (0·75 - 1·07) |
|  | 1905 | 0·79 (0·73 - 0·86) | 0·85 (0·71 - 1·02) |  | 0·84 (0·73 - 0·98) | 1·01 (0·79 - 1·28) |  | 0·76 (0·69 - 0·85) | 0·94 (0·83 - 1·07) |
|  | 1910 | 0·77 (0·73 - 0·82) | 0·85 (0·74 - 0·99) |  | 0·87 (0·79 - 0·97) | 1·19 (1·00 - 1·43) |  | 0·80 (0·74 - 0·87) | 0·98 (0·88 - 1·08) |
|  | 1915 | 0·74 (0·70 - 0·77) | 0·88 (0·78 - 1·00) |  | 0·74 (0·68 - 0·80) | 1·11 (0·95 - 1·29) |  | 0·75 (0·70 - 0·80) | 1·15 (1·07 - 1·25) |
|  | 1920 | 0·74 (0·71 - 0·77) | 0·78 (0·69 - 0·87) |  | 0·72 (0·67 - 0·77) | 0·86 (0·75 - 0·99) |  | 0·73 (0·69 - 0·77) | 0·99 (0·92 - 1·07) |
|  | 1925 | 0·81 (0·78 - 0·84) | 0·78 (0·70 - 0·87) |  | 0·81 (0·76 - 0·86) | 0·84 (0·74 - 0·96) |  | 0·77 (0·73 - 0·81) | 1·03 (0·97 - 1·10) |
|  | 1930 | 0·86 (0·83 - 0·89) | 0·94 (0·85 - 1·03) |  | 0·80 (0·76 - 0·85) | 0·85 (0·75 - 0·95) |  | 0·87 (0·83 - 0·91) | 0·97 (0·91 - 1·03) |
|  | 1935 | 0·86 (0·83 - 0·89) | 0·85 (0·78 - 0·94) |  | 0·81 (0·78 - 0·85) | 0·83 (0·74 - 0·93) |  | 0·85 (0·82 - 0·89) | 0·99 (0·94 - 1·04) |
|  | 1940 | 0·87 (0·85 - 0·90) | 0·90 (0·82 - 0·99) |  | 0·83 (0·80 - 0·87) | 0·84 (0·75 - 0·94) |  | 0·89 (0·86 - 0·93) | 0·99 (0·94 - 1·04) |
|  | 1945 | 0·88 (0·85 - 0·91) | 0·89 (0·80 - 0·98) |  | 0·85 (0·81 - 0·88) | 0·85 (0·76 - 0·96) |  | 0·92 (0·88 - 0·96) | 0·98 (0·92 - 1·04) |
|  | 1950 | 0·91 (0·88 - 0·94) | 0·90 (0·81 - 0·99) |  | 0·94 (0·91 - 0·98) | 0·89 (0·79 - 1·00) |  | 0·95 (0·91 - 0·99) | 0·97 (0·91 - 1·04) |
|  | 1955 | 1·00 (Reference) | 1·00 (Reference) |  | 1·00 (Reference) | 1·00 (Reference) |  | 1·00 (Reference) | 1·00 (0·93 - 1·07) |
|  | 1960 | 0·93 (0·90 - 0·96) | 0·97 (0·87 - 1·08) |  | 0·94 (0·90 - 0·98) | 1·12 (0·99 - 1·26) |  | 0·97 (0·92 - 1·01) | 1·06 (0·98 - 1·15) |
|  | 1965 | 0·79 (0·75 - 0·82) | 0·86 (0·75 - 0·98) |  | 0·82 (0·78 - 0·86) | 1·08 (0·94 - 1·23) |  | 0·82 (0·77 - 0·86) | 1·01 (0·91 - 1·11) |
|  | 1970 | 0·70 (0·66 - 0·74) | 0·78 (0·66 - 0·93) |  | 0·70 (0·66 - 0·75) | 0·98 (0·82 - 1·17) |  | 0·68 (0·63 - 0·72) | 0·98 (0·86 - 1·12) |
|  | 1975 | 0·58 (0·53 - 0·64) | 0·61 (0·46 - 0·80) |  | 0·58 (0·53 - 0·64) | 0·89 (0·70 - 1·14) |  | 0·53 (0·48 - 0·58) | 1·01 (0·83 - 1·22) |
|  | 1980 | 0·43 (0·36 - 0·51) | 0·43 (0·25 - 0·75) |  | 0·49 (0·41 - 0·58) | 0·85 (0·56 - 1·28) |  | 0·49 (0·41 - 0·58) | 1·22 (0·91 - 1·65) |

**Table 13.** Mortality rate (per 100,000 persons) and relative risk (95% confidence interval) from laryngeal, oropharyngeal and oral cavity cancer by gender in South region, 1980–2023.

|  |  | **Laryngeal Cancer** | |  | **Oropharyngeal Cancer** | |  | **Oral Cavity Cancer** | |
| --- | --- | --- | --- | --- | --- | --- | --- | --- | --- |
|  | | **Male** | **Female** |  | **Male** | **Female** |  | **Male** | **Female** |
|  |  | **Rate (95%CI)** | **Rate (95%CI)** |  | **Rate (95%CI)** | **Rate (95%CI)** |  | **Rate (95%CI)** | **Rate (95%CI)** |
| **Age Group** | 40 | 2·69 (2·46 - 2·93) | 0·25 (0·19 - 0·32) |  | 2·15 (1·93 - 2·4) | 0·12 (0·09 - 0·16) |  | 2·00 (1·80 - 2·22) | 0·27 (0·21 - 0·35) |
|  | 45 | 6·90 (6·45 - 7·38) | 0·61 (0·49 - 0·75) |  | 4·8o (4·4 - 5·24) | 0·28 (0·22 - 0·36) |  | 4·68 (4·29 - 5·10) | 0·43 (0·34 - 0·53) |
|  | 50 | 12·85 (12·09 - 13·65) | 1·10 (0·92 - 1·33) |  | 8·1o (7·46 - 8·79) | 0·51 (0·42 - 0·63) |  | 7·48 (6·89 - 8·12) | 0·74 (0·61 - 0·89) |
|  | 55 | 18·91 (17·82 - 20·07) | 1·79 (1·50 - 2·14) |  | 11·21 (10·34 - 12·16) | 0·88 (0·73 - 1·07) |  | 10·28 (9·48 - 11·14) | 1·26 (1·07 - 1·49) |
|  | 60 | 24·70 (23·27 - 26·22) | 2·52 (2·12 - 3·00) |  | 12·89 (11·86 - 14·01) | 0·97 (0·80 - 1·18) |  | 12·53 (11·55 - 13·59) | 1·48 (1·25 - 1·74) |
|  | 65 | 29·23 (27·50 - 31·08) | 3·39 (2·84 - 4·03) |  | 14·14 (12·94 - 15·44) | 1·22 (1·00 - 1·48) |  | 13·83 (12·71 - 15·05) | 2·10 (1·78 - 2·47) |
|  | 70 | 34·08 (31·87 - 36·46) | 3·74 (3·09 - 4·54) |  | 14·15 (12·80 - 15·64) | 1·81 (1·49 - 2·19) |  | 15·07 (13·72 - 16·54) | 2·89 (2·41 - 3·48) |
|  | 75 | 37·39 (34·80 - 40·17) | 4·55 (3·74 - 5·55) |  | 15·30 (13·74 - 17·04) | 1·98 (1·62 - 2·43) |  | 17·17 (15·55 - 18·96) | 3·91 (3·24 - 4·71) |
|  | 80 | 37·78 (35·08 - 40·69) | 5·41 (4·45 - 6·56) |  | 14·03 (12·54 - 15·79) | 3·55 (2·97 - 4·23) |  | 22·02 (19·99 - 24·25) | 8·32 (7·00 - 9·89) |
|  |  | **RR (95%CI)** | **RR (95%CI)** |  | **RR (95%CI)** | **RR (95%CI)** |  | **RR (95%CI)** | **RR (95%CI)** |
| **Period** | 1980 | 1·23 (1·15 - 1·31) | 1·24 (1·02 - 1·50) |  | 0·70 (0·63 - 0·78) | 0·60 (0·44 - 0·83) |  | 1·19 (1·09 - 1·30) | 0·98 (0·82 - 1·16) |
|  | 1985 | 1·20 (1·13 - 1·27) | 1·48 (1·25 - 1·75) |  | 0·73 (0·66 - 0·81) | 0·89 (0·69 - 1·15) |  | 1·23 (1·14 - 1·34) | 1·03 (0·89 - 1·19) |
|  | 1990 | 1·21 (1·14 - 1·28) | 1·37 (1·17 - 1·60) |  | 0·84 (0·77 - 0·92) | 1·10 (0·88 - 1·39) |  | 1·24 (1·15 - 1·34) | 1·06 (0·93 - 1·22) |
|  | 1995 | 1·18 (1·12 - 1·24) | 1·36 (1·17 - 1·58) |  | 1·02 (0·95 - 1·11) | 1·12 (0·90 - 1·40) |  | 1·07 (0·99 - 1·15) | 1·04 (0·92 - 1·18) |
|  | 2000 | 1·00 (Reference) | 1·00 (Reference) |  | 1·00 (Reference) | 1·00 (Reference) |  | 1·00 (Reference) | 0·96 (0·85 - 1·07) |
|  | 2005 | 0·99 (0·94 - 1·04) | 0·97 (0·84 - 1·12) |  | 0·98 (0·91 - 1·06) | 1·13 (0·92 - 1·37) |  | 0·92 (0·85 - 0·98) | 0·95 (0·86 - 1·06) |
|  | 2010 | 0·91 (0·86 - 0·95) | 0·87 (0·75 - 1·01) |  | 0·94 (0·88 - 1·01) | 1·14 (0·94 - 1·38) |  | 0·89 (0·83 - 0·95) | 0·98 (0·90 - 1·08) |
|  | 2015 | 0·81 (0·77 - 0·85) | 0·82 (0·71 - 0·95) |  | 0·99 (0·92 - 1·07) | 1·18 (0·98 - 1·42) |  | 0·86 (0·81 - 0·93) | 0·98 (0·90 - 1·07) |
|  | 2020 | 0·80 (0·75 - 0·84) | 0·82 (0·70 - 0·95) |  | 0·87 (0·80 - 0·94) | 1·00 (0·83 - 1·21) |  | 0·84 (0·78 - 0·90) | 1·05 (0·96 - 1·15) |
|  |  | **RR (95%CI)** | **RR (95%CI)** |  | **RR (95%CI)** | **RR (95%CI)** |  | **RR (95%CI)** | **RR (95%CI)** |
| **Birth Cohort** | 1900 | 0·59 (0·46 - 0·76) | 0·93 (0·58 - 1·49) |  | 1·14 (0·76 - 1·69) | 1·52 (0·82 - 2·83) |  | 1·12 (0·87 - 1·45) | 0·79 (0·49 - 1·26) |
|  | 1905 | 0·74 (0·65 - 0·86) | 0·68 (0·48 - 0·95) |  | 1·11 (0·88 - 1·40) | 1·03 (0·65 - 1·63) |  | 0·97 (0·82 - 1·15) | 1·20 (0·88 - 1·63) |
|  | 1910 | 0·75 (0·68 - 0·83) | 0·71 (0·55 - 0·93) |  | 0·60 (0·49 - 0·73) | 0·81 (0·56 - 1·17) |  | 0·85 (0·74 - 0·97) | 0·84 (0·62 - 1·12) |
|  | 1915 | 0·77 (0·70 - 0·83) | 0·75 (0·61 - 0·94) |  | 0·85 (0·74 - 0·97) | 1·05 (0·81 - 1·36) |  | 0·85 (0·76 - 0·95) | 1·11 (0·88 - 1·41) |
|  | 1920 | 0·83 (0·78 - 0·90) | 0·86 (0·71 - 1·04) |  | 0·75 (0·67 - 0·84) | 0·9 (0·71 - 1·12) |  | 0·79 (0·71 - 0·87) | 1·01 (0·82 - 1·26) |
|  | 1925 | 0·90 (0·84 - 0·96) | 0·88 (0·74 - 1·04) |  | 0·82 (0·74 - 0·91) | 0·92 (0·76 - 1·11) |  | 0·78 (0·72 - 0·86) | 0·85 (0·69 - 1·04) |
|  | 1930 | 0·93 (0·88 - 0·99) | 0·84 (0·71 - 0·99) |  | 0·78 (0·71 - 0·85) | 1·03 (0·89 - 1·2) |  | 0·86 (0·79 - 0·93) | 1·13 (0·94 - 1·35) |
|  | 1935 | 1·00 (0·94 - 1·05) | 0·98 (0·83 - 1·14) |  | 0·84 (0·77 - 0·91) | 1·06 (0·93 - 1·21) |  | 0·90 (0·83 - 0·97) | 1·17 (0·98 - 1·40) |
|  | 1940 | 0·97 (0·92 - 1·02) | 1·03 (0·88 - 1·20) |  | 0·83 (0·77 - 0·90) | 1·03 (0·9 - 1·18) |  | 0·89 (0·82 - 0·96) | 1·12 (0·94 - 1·33) |
|  | 1945 | 0·95 (0·90 - 1·00) | 0·91 (0·77 - 1·07) |  | 0·86 (0·80 - 0·93) | 0·89 (0·76 - 1·04) |  | 0·93 (0·87 - 1·01) | 1·03 (0·86 - 1·24) |
|  | 1950 | 0·97 (0·92 - 1·03) | 0·87 (0·74 - 1·03) |  | 0·88 (0·82 - 0·94) | 1·11 (0·97 - 1·28) |  | 0·98 (0·91 - 1·05) | 0·86 (0·71 - 1·04) |
|  | 1955 | 1·00 (Reference) | 1·00 (Reference) |  | 1·00 (Reference) | 1·04 (0·89 - 1·21) |  | 1·00 (Reference) | 1·00 (Reference) |
|  | 1960 | 0·95 (0·90 - 1·01) | 1·07 (0·90 - 1·27) |  | 0·93 (0·86 - 1·00) | 0·98 (0·83 - 1·17) |  | 0·97 (0·90 - 1·05) | 0·89 (0·72 - 1·09) |
|  | 1965 | 0·87 (0·81 - 0·93) | 0·82 (0·66 - 1·02) |  | 0·89 (0·82 - 0·97) | 0·99 (0·81 - 1·22) |  | 0·95 (0·87 - 1·04) | 0·91 (0·72 - 1·16) |
|  | 1970 | 0·71 (0·65 - 0·78) | 0·66 (0·48 - 0·89) |  | 0·74 (0·66 - 0·83) | 0·84 (0·61 - 1·16) |  | 0·80 (0·72 - 0·90) | 0·84 (0·61 - 1·14) |
|  | 1975 | 0·58 (0·50 - 0·68) | 0·64 (0·41 - 1·00) |  | 0·63 (0·54 - 0·74) | 0·93 (0·59 - 1·48) |  | 0·61 (0·52 - 0·73) | 1·03 (0·69 - 1·52) |
|  | 1980 | 0·41 (0·30 - 0·56) | 0·43 (0·16 - 1·15) |  | 0·49 (0·36 - 0·68) | 0·72 (0·27 - 1·91) |  | 0·55 (0·40 - 0·75) | 0·56 (0·25 - 1·22) |

**Table 14.** Mortality rate (per 100,000 persons) and relative risk (95% confidence interval) from laryngeal, oropharyngeal and oral cavity cancer by gender in Mid-west region, 1980–2023.

|  |  | **Laryngeal Cancer** | |  | **Oropharyngeal Cancer** | |  | **Oral Cavity Cancer** | |
| --- | --- | --- | --- | --- | --- | --- | --- | --- | --- |
|  | | **Male** | **Female** |  | **Male** | **Female** |  | **Male** | **Female** |
|  |  | **Rate (95%CI)** | **Rate (95%CI)** |  | **Rate (95%CI)** | **Rate (95%CI)** |  | **Rate (95%CI)** | **Rate (95%CI)** |
| **Age Group** | 40 | 1·42 (1·19 - 1·70) | 0·23 (0·14 - 0·37) |  | 1·30 (1·05 - 1·59) | 0·18 (0·12 - 0·25) |  | 1·15 (0·95 - 1·38) | 0·22 (0·16 - 0·30) |
|  | 45 | 3·77 (3·29 - 4·32) | 0·60 (0·42 - 0·86) |  | 2·67 (2·25 - 3·17) | 0·34 (0·25 - 0·44) |  | 2·75 (2·38 - 3·16) | 0·53 (0·42 - 0·66) |
|  | 50 | 7·31 (6·47 - 8·25) | 1·26 (0·92 - 1·72) |  | 4·55 (3·87 - 5·34) | 0·58 (0·46 - 0·73) |  | 4·73 (4·18 - 5·35) | 0·73 (0·60 - 0·91) |
|  | 55 | 11·5 (10·24 - 12·91) | 1·57 (1·15 - 2·14) |  | 6·29 (5·36 - 7·37) | 0·74 (0·58 - 0·93) |  | 7·34 (6·55 - 8·23) | 1·25 (1·04 - 1·50) |
|  | 60 | 15·01 (13·36 - 16·86) | 2·64 (1·94 - 3·58) |  | 7·42 (6·30 - 8·74) | 0·92 (0·73 - 1·17) |  | 8·52 (7·59 - 9·56) | 1·52 (1·26 - 1·83) |
|  | 65 | 18·88 (16·72 - 21·32) | 4·19 (3·09 - 5·69) |  | 7·98 (6·68 - 9·55) | 1·29 (1·03 - 1·63) |  | 10·24 (9·05 - 11·58) | 2·11 (1·76 - 2·53) |
|  | 70 | 22·92 (20·04 - 26·21) | 4·06 (2·85 - 5·79) |  | 9·24 (7·56 - 11·30) | 1·57 (1·22 - 2·02) |  | 12·43 (10·72 - 14·42) | 3·10 (2·59 - 3·71) |
|  | 75 | 25·44 (22·08 - 29·31) | 6·13 (4·31 - 8·74) |  | 9·44 (7·59 - 11·74) | 2·70 (2·14 - 3·41) |  | 14·29 (12·11 - 16·86) | 4·86 (4·08 - 5·80) |
|  | 80 | 26·97 (23·40 - 31·09) | 6·33 (4·44 - 9·00) |  | 9·75 (7·86 - 12·11) | 3·41 (2·78 - 4·19) |  | 17·44 (14·77 - 20·59) | 8·04 (7·00 - 9·22) |
|  |  | **RR (95%CI)** | **RR (95%CI)** |  | **RR (95%CI)** | **RR (95%CI)** |  | **RR (95%CI)** | **RR (95%CI)** |
| **Period** | 1980 | 0·79 (0·67 - 0·94) | 0·51 (0·30 - 0·85) |  | 0·47 (0·36 - 0·62) | 0·88 (0·54 - 1·43) |  | 0·91 (0·76 - 1·08) | 1·23 (0·90 - 1·67) |
|  | 1985 | 0·94 (0·81 - 1·08) | 0·95 (0·66 - 1·37) |  | 0·51 (0·39 - 0·65) | 0·74 (0·46 - 1·17) |  | 1·05 (0·91 - 1·22) | 0·70 (0·49 - 1·00) |
|  | 1990 | 0·94 (0·82 - 1·07) | 0·74 (0·51 - 1·07) |  | 0·69 (0·56 - 0·85) | 0·81 (0·55 - 1·20) |  | 0·93 (0·80 - 1·07) | 0·89 (0·67 - 1·18) |
|  | 1995 | 1·06 (0·94 - 1·20) | 0·90 (0·65 - 1·23) |  | 1·02 (0·86 - 1·22) | 1·27 (0·96 - 1·67) |  | 0·96 (0·84 - 1·08) | 0·93 (0·73 - 1·19) |
|  | 2000 | 1·00 (Reference) | 1·00 (Reference) |  | 1·00 (Reference) | 1·07 (0·84 - 1·36) |  | 1·10 (1·00 - 1·21) | 1·03 (0·85 - 1·24) |
|  | 2005 | 1·13 (1·02 - 1·25) | 0·85 (0·65 - 1·11) |  | 1·11 (0·96 - 1·28) | 1·00 (0·80 - 1·24) |  | 1·01 (0·93 - 1·11) | 1·15 (0·99 - 1·35) |
|  | 2010 | 1·25 (1·13 - 1·38) | 0·92 (0·72 - 1·18) |  | 1·24 (1·07 - 1·42) | 1·09 (0·90 - 1·31) |  | 1·03 (0·95 - 1·11) | 1·06 (0·91 - 1·22) |
|  | 2015 | 1·24 (1·12 - 1·37) | 0·70 (0·54 - 0·90) |  | 1·43 (1·24 - 1·65) | 1·10 (0·93 - 1·29) |  | 0·96 (0·89 - 1·03) | 1·00 (0·87 - 1·14) |
|  | 2020 | 1·10 (0·99 - 1·22) | 0·81 (0·63 - 1·05) |  | 1·30 (1·11 - 1·52) | 0·84 (0·70 - 1·01) |  | 1·00 (0·93 - 1·07) | 0·92 (0·80 - 1·06) |
|  |  | **RR (95%CI)** | **RR (95%CI)** |  | **RR (95%CI)** | **RR (95%CI)** |  | **RR (95%CI)** | **RR (95%CI)** |
| **Birth Cohort** | 1900 | 0·68 (0·35 - 1·32) | 1·51 (0·46 - 4·88) |  | 0·79 (0·21 - 2·91) | 0·63 (0·09 - 4·46) |  | 0·94 (0·48 - 1·84) | 0·35 (0·09 - 1·40) |
|  | 1905 | 0·82 (0·59 - 1·14) | 0·97 (0·48 - 1·97) |  | 1·40 (0·80 - 2·44) | 1·84 (0·88 - 3·87) |  | 0·79 (0·50 - 1·24) | 0·93 (0·48 - 1·79) |
|  | 1910 | 0·97 (0·77 - 1·21) | 0·87 (0·49 - 1·52) |  | 1·22 (0·82 - 1·81) | 0·90 (0·40 - 2·00) |  | 0·71 (0·50 - 1·00) | 1·07 (0·68 - 1·70) |
|  | 1915 | 0·92 (0·77 - 1·10) | 0·64 (0·39 - 1·04) |  | 0·77 (0·55 - 1·08) | 0·97 (0·56 - 1·66) |  | 0·63 (0·47 - 0·84) | 1·28 (0·92 - 1·79) |
|  | 1920 | 0·72 (0·62 - 0·85) | 0·64 (0·43 - 0·96) |  | 0·94 (0·73 - 1·21) | 1·31 (0·92 - 1·88) |  | 0·73 (0·59 - 0·91) | 0·78 (0·57 - 1·09) |
|  | 1925 | 0·87 (0·77 - 1·00) | 0·83 (0·59 - 1·16) |  | 0·67 (0·53 - 0·85) | 1·03 (0·74 - 1·44) |  | 0·84 (0·70 - 1·00) | 1·07 (0·85 - 1·34) |
|  | 1930 | 0·85 (0·76 - 0·96) | 0·66 (0·48 - 0·92) |  | 0·83 (0·68 - 1·00) | 0·99 (0·74 - 1·31) |  | 0·71 (0·60 - 0·84) | 0·97 (0·79 - 1·19) |
|  | 1935 | 0·82 (0·74 - 0·92) | 0·66 (0·49 - 0·89) |  | 0·75 (0·63 - 0·89) | 1·06 (0·84 - 1·34) |  | 0·81 (0·70 - 0·94) | 1·06 (0·90 - 1·26) |
|  | 1940 | 0·84 (0·75 - 0·93) | 0·78 (0·59 - 1·02) |  | 0·83 (0·71 - 0·96) | 0·95 (0·76 - 1·19) |  | 0·83 (0·72 - 0·95) | 1·02 (0·87 - 1·20) |
|  | 1945 | 0·82 (0·74 - 0·91) | 0·84 (0·64 - 1·10) |  | 0·85 (0·74 - 0·98) | 0·67 (0·50 - 0·90) |  | 0·88 (0·77 - 1·01) | 0·97 (0·80 - 1·17) |
|  | 1950 | 0·87 (0·79 - 0·96) | 0·83 (0·64 - 1·09) |  | 0·88 (0·78 - 1·01) | 0·95 (0·74 - 1·21) |  | 0·93 (0·82 - 1·06) | 0·97 (0·80 - 1·18) |
|  | 1955 | 1·00 (Reference) | 1·00 (Reference) |  | 1·00 (Reference) | 1·07 (0·84 - 1·35) |  | 1·00 (Reference) | 0·98 (0·80 - 1·20) |
|  | 1960 | 0·98 (0·89 - 1·08) | 1·18 (0·90 - 1·54) |  | 1·01 (0·89 - 1·15) | 1·09 (0·84 - 1·40) |  | 1·13 (0·99 - 1·28) | 0·97 (0·78 - 1·21) |
|  | 1965 | 0·77 (0·68 - 0·87) | 1·03 (0·75 - 1·42) |  | 0·92 (0·79 - 1·07) | 1·13 (0·86 - 1·50) |  | 1·02 (0·88 - 1·19) | 1·05 (0·82 - 1·35) |
|  | 1970 | 0·71 (0·61 - 0·83) | 0·61 (0·38 - 0·97) |  | 0·90 (0·75 - 1·08) | 1·04 (0·72 - 1·49) |  | 1·01 (0·85 - 1·21) | 1·03 (0·75 - 1·43) |
|  | 1975 | 0·76 (0·61 - 0·94) | 0·55 (0·28 - 1·11) |  | 0·73 (0·56 - 0·94) | 1·06 (0·64 - 1·75) |  | 0·71 (0·54 - 0·93) | 0·79 (0·48 - 1·31) |
|  | 1980 | 0·80 (0·55 - 1·17) | 0·20 (0·03 - 1·39) |  | 0·86 (0·59 - 1·26) | 1·08 (0·45 - 2·58) |  | 0·82 (0·53 - 1·27) | 1·40 (0·70 - 2·79) |

**Table 15.** Mortality rate (per 100,000 persons) and relative risk (95% confidence interval) from laryngeal, oropharyngeal and oral cavity cancer by gender in Northeast region, 1980–2023.

|  |  | **Laryngeal Cancer** | |  | **Oropharyngeal Cancer** | |  | **Oral Cavity Cancer** | |
| --- | --- | --- | --- | --- | --- | --- | --- | --- | --- |
|  | | **Male** | **Female** |  | **Male** | **Female** |  | **Male** | **Female** |
|  |  | **Rate (95%CI)** | **Rate (95%CI)** |  | **Rate (95%CI)** | **Rate (95%CI)** |  | **Rate (95%CI)** | **Rate (95%CI)** |
| **Age Group** | 40 | 0·94 (0·85 - 1·05) | 0·11 (0·08 - 0·13) |  | 0·75 (0·65 - 0·86) | 0·12 (0·09 - 0·15) |  | 1·36 (1·22 - 1·51) | 0·17 (0·14 - 0·22) |
|  | 45 | 2·25 (2·06 - 2·45) | 0·20 (0·17 - 0·25) |  | 1·59 (1·42 - 1·78) | 0·20 (0·16 - 0·25) |  | 2·77 (2·53 - 3·03) | 0·42 (0·35 - 0·51) |
|  | 50 | 4·04 (3·74 - 4·36) | 0·41 (0·35 - 0·48) |  | 2·63 (2·38 - 2·92) | 0·33 (0·27 - 0·4) |  | 4·41 (4·07 - 4·79) | 0·70 (0·60 - 0·83) |
|  | 55 | 5·83 (5·42 - 6·27) | 0·73 (0·63 - 0·85) |  | 3·54 (3·20 - 3·91) | 0·44 (0·37 - 0·53) |  | 5·58 (5·14 - 6·05) | 1·07 (0·92 - 1·24) |
|  | 60 | 7·96 (7·41 - 8·55) | 0·97 (0·84 - 1·13) |  | 4·04 (3·65 - 4·47) | 0·69 (0·58 - 0·83) |  | 6·79 (6·26 - 7·36) | 1·69 (1·46 - 1·95) |
|  | 65 | 9·69 (9·00 - 10·43) | 1·25 (1·08 - 1·45) |  | 4·23 (3·80 - 4·71) | 0·82 (0·69 - 0·98) |  | 7·41 (6·81 - 8·06) | 2·38 (2·07 - 2·74) |
|  | 70 | 12·03 (11·11 - 13·03) | 1·73 (1·49 - 2·00) |  | 4·28 (3·79 - 4·83) | 1·19 (1·00 - 1·41) |  | 8·75 (7·98 - 9·59) | 3·79 (3·27 - 4·39) |
|  | 75 | 12·54 (11·54 - 13·63) | 2·19 (1·89 - 2·54) |  | 4·92 (4·34 - 5·57) | 1·57 (1·32 - 1·87) |  | 10·07 (9·16 - 11·07) | 4·79 (4·13 - 5·56) |
|  | 80 | 14·69 (13·55 - 15·93) | 2·75 (2·40 - 3·15) |  | 5·20 (4·60 - 5·88) | 2·32 (1·98 - 2·71) |  | 14·22 (13·02 - 15·53) | 10·72 (9·33 - 12·32) |
|  |  | **RR (95%CI)** | **RR (95%CI)** |  | **RR (95%CI)** | **RR (95%CI)** |  | **RR (95%CI)** | **RR (95%CI)** |
| **Period** | 1980 | 0·70 (0·63 - 0·77) | 0·67 (0·52 - 0·85) |  | 0·47 (0·40 - 0·54) | 0·52 (0·38 - 0·70) |  | 0·74 (0·66 - 0·82) | 0·95 (0·80 - 1·11) |
|  | 1985 | 0·72 (0·66 - 0·79) | 0·73 (0·59 - 0·91) |  | 0·53 (0·46 - 0·61) | 0·75 (0·58 - 0·96) |  | 0·69 (0·63 - 0·77) | 0·68 (0·58 - 0·80) |
|  | 1990 | 0·82 (0·76 - 0·89) | 0·85 (0·69 - 1·03) |  | 0·63 (0·55 - 0·72) | 0·90 (0·72 - 1·13) |  | 0·73 (0·67 - 0·80) | 0·69 (0·60 - 0·80) |
|  | 1995 | 0·93 (0·86 - 1·00) | 0·98 (0·82 - 1·18) |  | 0·85 (0·76 - 0·95) | 0·90 (0·72 - 1·11) |  | 0·84 (0·77 - 0·92) | 0·90 (0·79 - 1·03) |
|  | 2000 | 1·00 (Reference) | 1·00 (Reference) |  | 1·00 (Reference) | 1·00 (Reference) |  | 1·00 (Reference) | 1·00 (Reference) |
|  | 2005 | 1·41 (1·32 - 1·50) | 1·50 (1·29 - 1·75) |  | 1·46 (1·33 - 1·60) | 1·49 (1·25 - 1·77) |  | 1·29 (1·20 - 1·38) | 1·42 (1·28 - 1·58) |
|  | 2010 | 1·67 (1·57 - 1·77) | 1·75 (1·52 - 2·02) |  | 1·63 (1·49 - 1·78) | 1·66 (1·40 - 1·96) |  | 1·32 (1·23 - 1·41) | 1·39 (1·25 - 1·55) |
|  | 2015 | 1·83 (1·72 - 1·94) | 1·96 (1·71 - 2·25) |  | 1·94 (1·77 - 2·11) | 1·94 (1·66 - 2·28) |  | 1·47 (1·38 - 1·58) | 1·27 (1·14 - 1·41) |
|  | 2020 | 1·84 (1·73 - 1·95) | 1·76 (1·53 - 2·03) |  | 2·08 (1·90 - 2·28) | 1·83 (1·55 - 2·15) |  | 1·42 (1·33 - 1·53) | 1·14 (1·02 - 1·27) |
|  |  | **RR (95%CI)** | **RR (95%CI)** |  | **RR (95%CI)** | **RR (95%CI)** |  | **RR (95%CI)** | **RR (95%CI)** |
| **Birth Cohort** | 1900 | 0·76 (0·56 - 1·04) | 0·77 (0·40 - 1·48) |  | 0·93 (0·53 - 1·64) | 0·52 (0·20 - 1·39) |  | 0·98 (0·74 - 1·29) | 0·71 (0·52 - 0·98) |
|  | 1905 | 0·95 (0·80 - 1·13) | 1·20 (0·86 - 1·67) |  | 1·00 (0·73 - 1·37) | 0·93 (0·60 - 1·43) |  | 0·86 (0·71 - 1·04) | 1·07 (0·86 - 1·34) |
|  | 1910 | 0·91 (0·80 - 1·03) | 1·18 (0·92 - 1·50) |  | 0·94 (0·74 - 1·17) | 0·91 (0·66 - 1·25) |  | 0·91 (0·79 - 1·05) | 1·10 (0·91 - 1·33) |
|  | 1915 | 0·80 (0·72 - 0·89) | 0·86 (0·69 - 1·08) |  | 0·82 (0·68 - 0·98) | 0·92 (0·71 - 1·19) |  | 0·70 (0·61 - 0·79) | 1·05 (0·89 - 1·24) |
|  | 1920 | 0·83 (0·76 - 0·91) | 0·89 (0·74 - 1·07) |  | 0·86 (0·74 - 0·99) | 0·74 (0·59 - 0·94) |  | 0·75 (0·68 - 0·83) | 0·98 (0·85 - 1·14) |
|  | 1925 | 0·98 (0·91 - 1·06) | 0·92 (0·80 - 1·06) |  | 1·05 (0·93 - 1·18) | 1·08 (0·93 - 1·25) |  | 0·89 (0·82 - 0·97) | 1·09 (0·96 - 1·25) |
|  | 1930 | 1·03 (0·96 - 1·10) | 0·96 (0·86 - 1·08) |  | 0·92 (0·82 - 1·03) | 1·03 (0·91 - 1·18) |  | 0·91 (0·84 - 0·98) | 1·10 (0·97 - 1·25) |
|  | 1935 | 0·98 (0·92 - 1·04) | 1·03 (0·94 - 1·14) |  | 0·90 (0·82 - 1·00) | 1·07 (0·96 - 1·19) |  | 0·92 (0·86 - 0·99) | 1·06 (0·94 - 1·19) |
|  | 1940 | 1·01 (0·95 - 1·07) | 1·07 (0·98 - 1·17) |  | 0·93 (0·85 - 1·02) | 1·08 (0·97 - 1·19) |  | 0·86 (0·80 - 0·93) | 1·10 (0·98 - 1·24) |
|  | 1945 | 0·96 (0·9 - 1·02) | 1·00 (0·90 - 1·10) |  | 0·88 (0·80 - 0·96) | 1·00 (0·88 - 1·12) |  | 0·88 (0·82 - 0·95) | 1·01 (0·88 - 1·14) |
|  | 1950 | 1·00 (0·94 - 1·06) | 1·06 (0·96 - 1·17) |  | 0·96 (0·89 - 1·04) | 0·98 (0·86 - 1·11) |  | 0·91 (0·85 - 0·98) | 0·92 (0·80 - 1·05) |
|  | 1955 | 1·00 (Reference) | 1·00 (0·89 - 1·11) |  | 1·00 (Reference) | 0·92 (0·80 - 1·06) |  | 1·00 (Reference) | 1·00 (Reference) |
|  | 1960 | 1·02 (0·96 - 1·09) | 0·95 (0·84 - 1·08) |  | 1·02 (0·94 - 1·11) | 1·04 (0·91 - 1·20) |  | 0·93 (0·87 - 1·00) | 1·01 (0·87 - 1·18) |
|  | 1965 | 0·92 (0·86 - 0·99) | 1·01 (0·87 - 1·16) |  | 0·92 (0·84 - 1·01) | 1·02 (0·87 - 1·20) |  | 0·82 (0·75 - 0·88) | 1·07 (0·91 - 1·27) |
|  | 1970 | 0·83 (0·76 - 0·91) | 0·97 (0·80 - 1·19) |  | 0·86 (0·77 - 0·97) | 0·89 (0·72 - 1·11) |  | 0·78 (0·71 - 0·86) | 0·87 (0·70 - 1·09) |
|  | 1975 | 0·77 (0·68 - 0·87) | 0·84 (0·62 - 1·14) |  | 0·88 (0·76 - 1·02) | 0·98 (0·74 - 1·29) |  | 0·68 (0·59 - 0·77) | 1·10 (0·83 - 1·46) |
|  | 1980 | 0·60 (0·48 - 0·76) | 0·78 (0·45 - 1·35) |  | 0·70 (0·54 - 0·89) | 0·80 (0·48 - 1·32) |  | 0·64 (0·52 - 0·80) | 1·77 (1·18 - 2·67) |

**Table 16.** Mortality rate (per 100,000 persons) and relative risk (95% confidence interval) from laryngeal, oropharyngeal and oral cavity cancer by gender in North region, 1980–2023.

|  |  | **Laryngeal Cancer** | |  | **Oropharyngeal Cancer** | |  | **Oral Cavity Cancer** | |
| --- | --- | --- | --- | --- | --- | --- | --- | --- | --- |
|  | | **Male** | **Female** |  | **Male** | **Female** |  | **Male** | **Female** |
|  |  | **Rate (95%CI)** | **Rate (95%CI)** |  | **Rate (95%CI)** | **Rate (95%CI)** |  | **Rate (95%CI)** | **Rate (95%CI)** |
| **Age Group** | 40 | 0·62 (0·51 - 0·76) | 0·18 (0·12 - 0·27) |  | 0·46 (0·35 - 0·60) | 0·09 (0·05 - 0·14) |  | 0·40 (0·31 - 0·51) | 0·13 (0·08 - 0·23) |
|  | 45 | 1·58 (1·35 - 1·85) | 0·19 (0·12 - 0·29) |  | 1·11 (0·89 - 1·38) | 0·10 (0·06 - 0·17) |  | 1·02 (0·83 - 1·25) | 0·18 (0·11 - 0·30) |
|  | 50 | 2·86 (2·49 - 3·30) | 0·39 (0·26 - 0·56) |  | 1·59 (1·29 - 1·96) | 0·20 (0·13 - 0·30) |  | 1·58 (1·30 - 1·93) | 0·40 (0·25 - 0·62) |
|  | 55 | 5·76 (5·07 - 6·54) | 0·67 (0·47 - 0·94) |  | 3·05 (2·52 - 3·68) | 0·49 (0·37 - 0·67) |  | 2·91 (2·43 - 3·49) | 0·63 (0·41 - 0·96) |
|  | 60 | 8·58 (7·58 - 9·70) | 1·11 (0·80 - 1·54) |  | 3·45 (2·84 - 4·20) | 0·69 (0·52 - 0·92) |  | 4·11 (3·44 - 4·91) | 0·85 (0·56 - 1·28) |
|  | 65 | 11·73 (10·37 - 13·26) | 1·47 (1·06 - 2·03) |  | 4·34 (3·56 - 5·29) | 0·99 (0·75 - 1·31) |  | 4·53 (3·77 - 5·45) | 1·56 (1·07 - 2·28) |
|  | 70 | 13·11 (11·52 - 14·92) | 2·12 (1·53 - 2·94) |  | 4·71 (3·82 - 5·82) | 1·11 (0·82 - 1·52) |  | 5·55 (4·58 - 6·72) | 2·52 (1·67 - 3·79) |
|  | 75 | 16·76 (14·64 - 19·17) | 2·95 (2·12 - 4·09) |  | 5·07 (4·02 - 6·40) | 1·97 (1·49 - 2·62) |  | 5·49 (4·43 - 6·79) | 3·97 (2·66 - 5·93) |
|  | 80 | 18·94 (16·62 - 21·60) | 4·08 (3·03 - 5·51) |  | 5·82 (4·65 - 7·28) | 2·68 (2·12 - 3·38) |  | 8·60 (7·10 - 10·4) | 5·89 (3·97 - 8·73) |
|  |  | **RR (95%CI)** | **RR (95%CI)** |  | **RR (95%CI)** | **RR (95%CI)** |  | **RR (95%CI)** | **RR (95%CI)** |
| **Period** | 1980 | 1·26 (1·06 - 1·51) | 1·77 (1·17 - 2·69) |  | 0·39 (0·25 - 0·60) | 1·16 (0·62 - 2·15) |  | 1·16 (0·89 - 1·52) | 1·86 (1·22 - 2·85) |
|  | 1985 | 1·04 (0·87 - 1·23) | 1·09 (0·70 - 1·71) |  | 0·51 (0·37 - 0·73) | 1·21 (0·73 - 2·00) |  | 1·06 (0·82 - 1·37) | 1·63 (1·10 - 2·43) |
|  | 1990 | 0·92 (0·77 - 1·09) | 1·20 (0·80 - 1·81) |  | 0·56 (0·41 - 0·77) | 1·16 (0·74 - 1·83) |  | 0·95 (0·74 - 1·22) | 0·88 (0·57 - 1·35) |
|  | 1995 | 0·91 (0·77 - 1·07) | 1·07 (0·72 - 1·60) |  | 0·68 (0·52 - 0·89) | 1·06 (0·70 - 1·59) |  | 1·12 (0·89 - 1·40) | 0·87 (0·57 - 1·31) |
|  | 2000 | 1·00 (Reference) | 1·00 (Reference) |  | 1·00 (Reference) | 0·70 (0·48 - 1·04) |  | 1·00 (Reference) | 1·00 (Reference) |
|  | 2005 | 1·10 (0·96 - 1·26) | 0·94 (0·65 - 1·34) |  | 1·06 (0·86 - 1·31) | 0·98 (0·74 - 1·31) |  | 1·27 (1·04 - 1·53) | 1·16 (0·84 - 1·60) |
|  | 2010 | 1·37 (1·21 - 1·55) | 1·37 (1·00 - 1·88) |  | 1·09 (0·89 - 1·33) | 0·92 (0·71 - 1·19) |  | 1·34 (1·12 - 1·61) | 1·62 (1·19 - 2·19) |
|  | 2015 | 1·38 (1·22 - 1·56) | 1·39 (1·03 - 1·89) |  | 1·44 (1·20 - 1·73) | 1·03 (0·84 - 1·27) |  | 1·62 (1·36 - 1·92) | 1·37 (1·01 - 1·86) |
|  | 2020 | 1·28 (1·13 - 1·45) | 1·24 (0·90 - 1·69) |  | 1·33 (1·10 - 1·60) | 1·06 (0·87 - 1·30) |  | 1·36 (1·13 - 1·62) | 1·52 (1·12 - 2·06) |
|  |  | **RR (95%CI)** | **RR (95%CI)** |  | **RR (95%CI)** | **RR (95%CI)** |  | **RR (95%CI)** | **RR (95%CI)** |
| **Birth Cohort** | 1900 | 1·29 (0·78 - 2·15) | 0·62 (0·20 - 1·91) |  |  | 0·70 (0·10 - 4·95) |  | 0·62 (0·20 - 1·93) | 1·22 (0·58 - 2·56) |
|  | 1905 | 0·95 (0·67 - 1·35) | 0·85 (0·42 - 1·69) |  | 1·27 (0·53 - 3·04) | 0·75 (0·24 - 2·33) |  | 0·97 (0·55 - 1·71) | 1·07 (0·62 - 1·83) |
|  | 1910 | 0·97 (0·75 - 1·25) | 1·20 (0·76 - 1·88) |  | 1·80 (1·10 - 2·93) | 1·44 (0·78 - 2·68) |  | 1·17 (0·80 - 1·69) | 1·30 (0·82 - 2·07) |
|  | 1915 | 0·93 (0·76 - 1·13) | 0·99 (0·65 - 1·50) |  | 1·07 (0·68 - 1·68) | 1·03 (0·57 - 1·86) |  | 1·27 (0·97 - 1·65) | 1·25 (0·82 - 1·90) |
|  | 1920 | 0·80 (0·67 - 0·95) | 0·81 (0·55 - 1·18) |  | 0·89 (0·63 - 1·26) | 0·87 (0·50 - 1·49) |  | 0·69 (0·52 - 0·92) | 0·66 (0·42 - 1·03) |
|  | 1925 | 1·05 (0·92 - 1·19) | 1·10 (0·81 - 1·48) |  | 0·86 (0·65 - 1·14) | 1·07 (0·71 - 1·59) |  | 1·07 (0·89 - 1·29) | 1·37 (0·96 - 1·95) |
|  | 1930 | 0·98 (0·88 - 1·10) | 1·10 (0·86 - 1·41) |  | 0·94 (0·75 - 1·17) | 1·32 (0·97 - 1·80) |  | 0·96 (0·81 - 1·14) | 1·06 (0·75 - 1·49) |
|  | 1935 | 1·02 (0·93 - 1·12) | 0·76 (0·58 - 0·98) |  | 1·10 (0·93 - 1·29) | 1·05 (0·78 - 1·40) |  | 1·04 (0·91 - 1·19) | 1·38 (1·00 - 1·90) |
|  | 1940 | 1·03 (0·95 - 1·13) | 0·98 (0·80 - 1·22) |  | 1·05 (0·91 - 1·22) | 0·76 (0·56 - 1·03) |  | 0·94 (0·83 - 1·08) | 1·06 (0·77 - 1·47) |
|  | 1945 | 1·01 (0·92 - 1·1) | 1·30 (1·06 - 1·60) |  | 0·94 (0·81 - 1·10) | 1·06 (0·79 - 1·42) |  | 0·95 (0·83 - 1·08) | 0·73 (0·51 - 1·05) |
|  | 1950 | 1·04 (0·96 - 1·14) | 1·00 (0·79 - 1·28) |  | 0·98 (0·85 - 1·12) | 0·83 (0·59 - 1·17) |  | 1·06 (0·94 - 1·20) | 0·96 (0·67 - 1·37) |
|  | 1955 | 1·08 (0·99 - 1·18) | 1·14 (0·90 - 1·45) |  | 1·01 (0·88 - 1·16) | 1·02 (0·75 - 1·40) |  | 1·04 (0·92 - 1·18) | 1·00 (Reference) |
|  | 1960 | 0·98 (0·89 - 1·09) | 0·90 (0·67 - 1·22) |  | 0·96 (0·83 - 1·11) | 0·95 (0·66 - 1·37) |  | 1·08 (0·95 - 1·23) | 1·08 (0·74 - 1·59) |
|  | 1965 | 0·91 (0·80 - 1·03) | 0·88 (0·61 - 1·28) |  | 1·06 (0·90 - 1·24) | 0·90 (0·58 - 1·42) |  | 0·91 (0·77 - 1·08) | 1·37 (0·92 - 2·06) |
|  | 1970 | 0·87 (0·73 - 1·04) | 1·12 (0·74 - 1·68) |  | 0·97 (0·78 - 1·20) | 0·99 (0·55 - 1·79) |  | 1·06 (0·87 - 1·29) | 1·19 (0·72 - 1·97) |
|  | 1975 | 0·94 (0·73 - 1·20) | 0·66 (0·33 - 1·31) |  | 0·91 (0·68 - 1·21) | 1·62 (0·89 - 2·92) |  | 0·79 (0·57 - 1·09) | 1·17 (0·61 - 2·26) |
|  | 1980 | 0·91 (0·58 - 1·43) | 0·51 (0·17 - 1·59) |  | 1·51 (1·01 - 2·26) | 1·74 (0·78 - 3·87) |  | 0·85 (0·48 - 1·50) | 1·31 (0·54 - 3·19) |

**Table 17.** Mortality rate (per 100,000 persons) and relative risk (95% confidence interval) from laryngeal, oropharyngeal and oral cavity cancer in white Brazilians by gender, 2000–2023.

|  |  | **Laryngeal Cancer** | |  | **Oropharyngeal Cancer** | |  | **Oral Cavity Cancer** | |
| --- | --- | --- | --- | --- | --- | --- | --- | --- | --- |
|  | | **Male** | **Female** |  | **Male** | **Female** |  | **Male** | **Female** |
|  |  | **Rate (95%CI)** | **Rate (95%CI)** |  | **Rate (95%CI)** | **Rate (95%CI)** |  | **Rate (95%CI)** | **Rate (95%CI)** |
| **Age Group** | 40 | 2·02 (1·88 - 2·16) | 0·21 (0·17 - 0·27) |  | 1·37 (1·25 - 1·50) | 0·12 (0·09 - 0·14) |  | 1·50 (1·38 - 1·63) | 0·21 (0·18 - 0·25) |
|  | 45 | 5·36 (5·12 - 5·62) | 0·48 (0·42 - 0·56) |  | 3·11 (2·92 - 3·31) | 0·25 (0·21 - 0·28) |  | 3·46 (3·26 - 3·67) | 0·40 (0·36 - 0·45) |
|  | 50 | 10·21 (9·82 - 10·62) | 0·83 (0·74 - 0·93) |  | 5·50 (5·21 - 5·81) | 0·43 (0·38 - 0·48) |  | 5·67 (5·38 - 5·97) | 0·66 (0·60 - 0·72) |
|  | 55 | 15·66 (15·11 - 16·24) | 1·31 (1·18 - 1·44) |  | 7·93 (7·53 - 8·34) | 0·70 (0·64 - 0·78) |  | 8·18 (7·79 - 8·60) | 1·06 (0·98 - 1·15) |
|  | 60 | 20·94 (20·22 - 21·70) | 1·85 (1·69 - 2·03) |  | 9·21 (8·75 - 9·69) | 0·82 (0·74 - 0·90) |  | 9·97 (9·49 - 10·47) | 1·54 (1·44 - 1·66) |
|  | 65 | 26·77 (25·79 - 27·78) | 2·27 (2·06 - 2·50) |  | 10·56 (10·00 - 11·15) | 1·18 (1·07 - 1·29) |  | 11·66 (11·07 - 12·28) | 2·21 (2·07 - 2·37) |
|  | 70 | 31·78 (30·44 - 33·17) | 2·72 (2·42 - 3·06) |  | 10·40 (9·75 - 11·10) | 1·55 (1·41 - 1·69) |  | 12·82 (12·07 - 13·62) | 3·03 (2·84 - 3·24) |
|  | 75 | 36·53 (34·86 - 38·29) | 3·03 (2·66 - 3·44) |  | 10·95 (10·19 - 11·76) | 1·87 (1·70 - 2·06) |  | 14·05 (13·15 - 15·01) | 4·66 (4·39 - 4·96) |
|  | 80 | 41·90 (39·92 - 43·98) | 4·02 (3·53 - 4·57) |  | 10·98 (10·19 - 11·82) | 2·94 (2·75 - 3·16) |  | 19·43 (18·23 - 20·72) | 10·30 (9·92 - 10·69) |
|  |  | **RR (95%CI)** | **RR (95%CI)** |  | **RR (95%CI)** | **RR (95%CI)** |  | **RR (95%CI)** | **RR (95%CI)** |
| **Period** | 2000 | 1·16 (1·12 - 1·19) | 0·96 (0·90 - 1·02) |  | 1·16 (1·10 - 1·21) | 0·98 (0·90 - 1·07) |  | 1·17 (1·12 - 1·22) | 0·97 (0·91 - 1·02) |
|  | 2005 | 1·12 (1·09 - 1·15) | 1·05 (0·99 - 1·11) |  | 1·07 (1·02 - 1·12) | 1·03 (0·95 - 1·11) |  | 1·10 (1·06 - 1·15) | 1·04 (0·99 - 1·10) |
|  | 2010 | 1·00 (Reference) | 1·01 (0·95 - 1·06) |  | 1·00 (Reference) | 1·02 (0·95 - 1·10) |  | 1·00 (Reference) | 0·99 (0·95 - 1·04) |
|  | 2015 | 0·88 (0·86 - 0·91) | 0·99 (0·94 - 1·05) |  | 1·01 (0·96 - 1·05) | 0·95 (0·89 - 1·02) |  | 0·99 (0·95 - 1·03) | 1·00 (0·96 - 1·05) |
|  | 2020 | 0·79 (0·77 - 0·82) | 0·99 (0·94 - 1·05) |  | 1·00 (0·96 - 1·05) | 1·03 (0·96 - 1·10) |  | 0·92 (0·88 - 0·96) | 0·99 (0·95 - 1·04) |
|  |  | **RR (95%CI)** | **RR (95%CI)** |  | **RR (95%CI)** | **RR (95%CI)** |  | **RR (95%CI)** | **RR (95%CI)** |
| **Birth Cohort** | 1920 | 0·58 (0·53 - 0·64) | 0·91 (0·73 - 1·13) |  | 0·73 (0·63 - 0·85) | 0·97 (0·79 - 1·18) |  | 0·65 (0·58 - 0·73) | 0·95 (0·85 - 1·05) |
|  | 1925 | 0·66 (0·62 - 0·70) | 1·10 (0·93 - 1·30) |  | 0·80 (0·73 - 0·88) | 1·08 (0·94 - 1·24) |  | 0·72 (0·66 - 0·78) | 1·00 (0·93 - 1·08) |
|  | 1930 | 0·75 (0·71 - 0·79) | 1·18 (1·02 - 1·35) |  | 0·71 (0·66 - 0·77) | 1·01 (0·91 - 1·13) |  | 0·74 (0·69 - 0·79) | 0·99 (0·93 - 1·06) |
|  | 1935 | 0·78 (0·75 - 0·81) | 1·08 (0·95 - 1·22) |  | 0·76 (0·71 - 0·81) | 1·01 (0·92 - 1·11) |  | 0·80 (0·76 - 0·85) | 1·04 (0·99 - 1·10) |
|  | 1940 | 0·83 (0·80 - 0·86) | 1·08 (0·96 - 1·21) |  | 0·82 (0·78 - 0·87) | 0·95 (0·87 - 1·04) |  | 0·84 (0·79 - 0·88) | 1·01 (0·96 - 1·06) |
|  | 1945 | 0·87 (0·84 - 0·90) | 1·01 (0·91 - 1·13) |  | 0·85 (0·81 - 0·90) | 0·92 (0·83 - 1·01) |  | 0·88 (0·84 - 0·92) | 1·01 (0·94 - 1·08) |
|  | 1950 | 0·92 (0·89 - 0·96) | 0·93 (0·84 - 1·03) |  | 0·92 (0·88 - 0·97) | 1·05 (0·95 - 1·15) |  | 0·95 (0·91 - 1·00) | 0·93 (0·86 - 1·00) |
|  | 1955 | 1·00 (Reference) | 1·00 (Reference) |  | 1·00 (Reference) | 1·05 (0·95 - 1·16) |  | 1·00 (Reference) | 0·99 (0·92 - 1·07) |
|  | 1960 | 0·94 (0·91 - 0·97) | 0·96 (0·85 - 1·07) |  | 0·93 (0·89 - 0·98) | 1·00 (0·89 - 1·12) |  | 0·97 (0·93 - 1·02) | 0·99 (0·90 - 1·08) |
|  | 1965 | 0·84 (0·81 - 0·88) | 0·73 (0·63 - 0·84) |  | 0·84 (0·79 - 0·89) | 1·07 (0·93 - 1·22) |  | 0·83 (0·79 - 0·88) | 1·05 (0·95 - 1·17) |
|  | 1970 | 0·71 (0·67 - 0·76) | 0·58 (0·47 - 0·72) |  | 0·73 (0·68 - 0·79) | 0·92 (0·76 - 1·12) |  | 0·74 (0·69 - 0·80) | 0·95 (0·82 - 1·10) |
|  | 1975 | 0·61 (0·56 - 0·67) | 0·44 (0·31 - 0·61) |  | 0·65 (0·58 - 0·72) | 0·94 (0·71 - 1·25) |  | 0·59 (0·53 - 0·66) | 1·04 (0·85 - 1·28) |
|  | 1980 | 0·45 (0·37 - 0·55) | 0·36 (0·19 - 0·67) |  | 0·58 (0·47 - 0·70) | 0·93 (0·57 - 1·52) |  | 0·58 (0·47 - 0·70) | 1·24 (0·90 - 1·71) |

**Table 18.** Mortality rate (per 100,000 persons) and relative risk (95% confidence interval) from laryngeal, oropharyngeal and oral cavity cancer in black Brazilians by gender, 2000–2023.

|  |  | **Laryngeal Cancer** | |  | **Oropharyngeal Cancer** | |  | **Oral Cavity Cancer** | |
| --- | --- | --- | --- | --- | --- | --- | --- | --- | --- |
|  | | **Male** | **Female** |  | **Male** | **Female** |  | **Male** | **Female** |
|  |  | **Rate (95%CI)** | **Rate (95%CI)** |  | **Rate (95%CI)** | **Rate (95%CI)** |  | **Rate (95%CI)** | **Rate (95%CI)** |
| **Age Group** | 40 | 2·62 (2·21 - 3·12) | 0·31 (0·22 - 0·43) |  | 1·94 (1·60 - 2·37) | 0·37 (0·23 - 0·60) |  | 2·11 (1·76 - 2·54) | 0·26 (0·19 - 0·36) |
|  | 45 | 5·80 (5·17 - 6·52) | 0·66 (0·51 - 0·85) |  | 4·09 (3·56 - 4·69) | 0·73 (0·52 - 1·02) |  | 3·44 (2·97 - 3·99) | 0·54 (0·43 - 0·69) |
|  | 50 | 9·15 (8·33 - 10·05) | 1·03 (0·82 - 1·29) |  | 6·02 (5·36 - 6·75) | 0·97 (0·72 - 1·29) |  | 5·63 (4·95 - 6·40) | 1·17 (0·98 - 1·39) |
|  | 55 | 12·17 (11·18 - 13·24) | 1·63 (1·33 - 2·00) |  | 7·87 (7·09 - 8·75) | 1·28 (0·99 - 1·65) |  | 7·34 (6·48 - 8·31) | 1·70 (1·45 - 1·99) |
|  | 60 | 14·87 (13·72 - 16·13) | 2·30 (1·89 - 2·80) |  | 9·20 (8·30 - 10·21) | 1·31 (1·02 - 1·70) |  | 8·17 (7·20 - 9·26) | 2·06 (1·76 - 2·41) |
|  | 65 | 17·33 (15·85 - 18·94) | 2·36 (1·91 - 2·92) |  | 9·55 (8·48 - 10·76) | 1·64 (1·23 - 2·17) |  | 9·74 (8·54 - 11·10) | 2·34 (1·97 - 2·77) |
|  | 70 | 18·05 (16·07 - 20·28) | 2·78 (2·22 - 3·48) |  | 8·60 (7·28 - 10·16) | 2·08 (1·45 - 2·98) |  | 9·55 (8·12 - 11·23) | 3·07 (2·59 - 3·65) |
|  | 75 | 19·98 (17·41 - 22·92) | 3·48 (2·75 - 4·40) |  | 8·58 (6·95 - 10·59) | 2·14 (1·39 - 3·29) |  | 11·38 (9·52 - 13·61) | 3·83 (3·18 - 4·62) |
|  | 80 | 19·87 (17·09 - 23·10) | 4·15 (3·36 - 5·11) |  | 10·10 (8·06 - 12·67) | 3·64 (2·39 - 5·56) |  | 12·99 (10·90 - 15·48) | 6·87 (6·04 - 7·82) |
|  |  | **RR (95%CI)** | **RR (95%CI)** |  | **RR (95%CI)** | **RR (95%CI)** |  | **RR (95%CI)** | **RR (95%CI)** |
| **Period** | 2000 | 0·97 (0·91 - 1·04) | 0·80 (0·63 - 1·01) |  | 1·03 (0·93 - 1·13) | 1·03 (0·83 - 1·28) |  | 1·11 (0·98 - 1·24) | 0·97 (0·83 - 1·13) |
|  | 2005 | 1·02 (0·96 - 1·08) | 1·12 (0·92 - 1·36) |  | 1·03 (0·95 - 1·11) | 1·09 (0·91 - 1·31) |  | 1·19 (1·07 - 1·32) | 1·08 (0·94 - 1·24) |
|  | 2010 | 1·03 (0·98 - 1·09) | 1·00 (1·00 - 1·00) |  | 0·94 (0·88 - 1·02) | 0·88 (0·74 - 1·05) |  | 1·00 (Reference) | 0·99 (0·87 - 1·12) |
|  | 2015 | 0·97 (0·92 - 1·02) | 0·78 (0·65 - 0·95) |  | 0·99 (0·93 - 1·06) | 0·96 (0·83 - 1·11) |  | 0·91 (0·82 - 1·00) | 0·94 (0·83 - 1·05) |
|  | 2020 | 1·01 (0·96 - 1·06) | 0·77 (0·64 - 0·93) |  | 1·03 (0·96 - 1·10) | 1·06 (0·92 - 1·23) |  | 0·95 (0·85 - 1·05) | 1·05 (0·93 - 1·18) |
|  |  | **RR (95%CI)** | **RR (95%CI)** |  | **RR (95%CI)** | **RR (95%CI)** |  | **RR (95%CI)** | **RR (95%CI)** |
| **Birth Cohort** | 1920 | 0·68 (0·47 - 0·97) | 1·40 (0·88 - 2·21) |  | 0·55 (0·32 - 0·96) | 0·56 (0·25 - 1·27) |  | 0·73 (0·50 - 1·06) | 0·94 (0·65 - 1·36) |
|  | 1925 | 0·83 (0·66 - 1·04) | 0·88 (0·61 - 1·27) |  | 0·70 (0·49 - 1·00) | 0·66 (0·36 - 1·23) |  | 0·66 (0·51 - 0·85) | 0·90 (0·68 - 1·19) |
|  | 1930 | 1·02 (0·86 - 1·20) | 0·89 (0·68 - 1·17) |  | 0·77 (0·60 - 1·01) | 0·69 (0·42 - 1·14) |  | 0·83 (0·69 - 1·00) | 1·03 (0·83 - 1·26) |
|  | 1935 | 1·04 (0·90 - 1·19) | 0·90 (0·72 - 1·13) |  | 0·78 (0·63 - 0·95) | 0·87 (0·58 - 1·32) |  | 0·70 (0·59 - 0·83) | 1·02 (0·86 - 1·22) |
|  | 1940 | 0·94 (0·84 - 1·06) | 1·06 (0·88 - 1·26) |  | 0·80 (0·68 - 0·95) | 0·71 (0·49 - 1·04) |  | 0·78 (0·68 - 0·90) | 1·11 (0·96 - 1·29) |
|  | 1945 | 0·93 (0·84 - 1·04) | 0·91 (0·76 - 1·11) |  | 0·81 (0·70 - 0·93) | 0·77 (0·55 - 1·08) |  | 0·94 (0·83 - 1·07) | 0·93 (0·77 - 1·11) |
|  | 1950 | 0·98 (0·89 - 1·07) | 1·04 (0·88 - 1·24) |  | 0·96 (0·86 - 1·09) | 0·86 (0·64 - 1·15) |  | 1·00 (0·89 - 1·13) | 0·94 (0·79 - 1·11) |
|  | 1955 | 1·00 (Reference) | 1·13 (0·96 - 1·33) |  | 1·00 (Reference) | 1·00 (Reference) |  | 1·00 (Reference) | 1·00 (0·84 - 1·18) |
|  | 1960 | 0·88 (0·80 - 0·97) | 1·11 (0·93 - 1·32) |  | 0·92 (0·82 - 1·04) | 1·15 (0·87 - 1·51) |  | 1·01 (0·90 - 1·13) | 1·07 (0·90 - 1·27) |
|  | 1965 | 0·65 (0·58 - 0·72) | 0·95 (0·76 - 1·19) |  | 0·76 (0·67 - 0·87) | 0·71 (0·50 - 0·99) |  | 0·86 (0·75 - 0·98) | 0·94 (0·75 - 1·16) |
|  | 1970 | 0·61 (0·53 - 0·70) | 0·81 (0·59 - 1·11) |  | 0·68 (0·58 - 0·80) | 0·42 (0·26 - 0·67) |  | 0·77 (0·66 - 0·90) | 1·07 (0·82 - 1·40) |
|  | 1975 | 0·51 (0·42 - 0·62) | 0·97 (0·64 - 1·45) |  | 0·51 (0·41 - 0·65) | 0·57 (0·33 - 0·99) |  | 0·56 (0·45 - 0·70) | 0·85 (0·54 - 1·33) |
|  | 1980 | 0·34 (0·23 - 0·50) | 0·48 (0·18 - 1·29) |  | 0·39 (0·26 - 0·59) | 0·62 (0·27 - 1·44) |  | 0·46 (0·32 - 0·66) | 1·13 (0·59 - 2·16) |

**Table 19.** Mortality rate (per 100,000 persons) and relative risk (95% confidence interval) from laryngeal, oropharyngeal and oral cavity cancer in brown Brazilians by gender, 2000–2023.

|  |  | **Laryngeal Cancer** | |  | **Oropharyngeal Cancer** | |  | **Oral Cavity Cancer** | |
| --- | --- | --- | --- | --- | --- | --- | --- | --- | --- |
|  | | **Male** | **Female** |  | **Male** | **Female** |  | **Male** | **Female** |
|  |  | **Rate (95%CI)** | **Rate (95%CI)** |  | **Rate (95%CI)** | **Rate (95%CI)** |  | **Rate (95%CI)** | **Rate (95%CI)** |
| **Age Group** | 40 | 1·69 (1·54 - 1·85) | 0·17 (0·14 - 0·21) |  | 1·27 (1·14 - 1·42) | 0·14 (0·11 - 0·19) |  | 1·55 (1·40 - 1·71) | 0·19 (0·15 - 0·24) |
|  | 45 | 4·12 (3·86 - 4·40) | 0·36 (0·31 - 0·42) |  | 2·66 (2·45 - 2·89) | 0·26 (0·21 - 0·32) |  | 3·05 (2·83 - 3·30) | 0·43 (0·36 - 0·51) |
|  | 50 | 7·13 (6·75 - 7·53) | 0·78 (0·69 - 0·87) |  | 4·48 (4·17 - 4·81) | 0·47 (0·40 - 0·56) |  | 4·88 (4·56 - 5·22) | 0·86 (0·74 - 1·00) |
|  | 55 | 10·84 (10·32 - 11·40) | 1·27 (1·14 - 1·41) |  | 5·95 (5·56 - 6·36) | 0·73 (0·62 - 0·85) |  | 6·91 (6·49 - 7·36) | 1·19 (1·04 - 1·37) |
|  | 60 | 14·39 (13·71 - 15·09) | 1·83 (1·65 - 2·03) |  | 6·89 (6·45 - 7·37) | 0·98 (0·84 - 1·14) |  | 7·83 (7·36 - 8·34) | 1·73 (1·53 - 1·97) |
|  | 65 | 18·03 (17·15 - 18·96) | 2·26 (2·03 - 2·51) |  | 7·11 (6·61 - 7·65) | 1·37 (1·17 - 1·60) |  | 8·79 (8·23 - 9·40) | 2·41 (2·12 - 2·74) |
|  | 70 | 21·09 (19·87 - 22·38) | 2·73 (2·44 - 3·05) |  | 7·28 (6·67 - 7·96) | 1·83 (1·50 - 2·23) |  | 9·66 (8·92 - 10·47) | 3·74 (3·24 - 4·30) |
|  | 75 | 24·83 (23·27 - 26·49) | 3·52 (3·13 - 3·95) |  | 7·91 (7·17 - 8·73) | 2·69 (2·16 - 3·34) |  | 12·04 (11·05 - 13·12) | 4·84 (4·17 - 5·60) |
|  | 80 | 27·78 (26·04 - 29·64) | 4·37 (3·95 - 4·85) |  | 8·52 (7·74 - 9·39) | 4·57 (3·68 - 5·69) |  | 15·52 (14·29 - 16·85) | 9·06 (7·89 - 10·41) |
|  |  | **RR (95%CI)** | **RR (95%CI)** |  | **RR (95%CI)** | **RR (95%CI)** |  | **RR (95%CI)** | **RR (95%CI)** |
| **Period** | 2000 | 0·69 (0·66 - 0·73) | 0·71 (0·63 - 0·81) |  | 0·76 (0·71 - 0·82) | 0·91 (0·80 - 1·04) |  | 0·82 (0·77 - 0·88) | 0·70 (0·62 - 0·79) |
|  | 2005 | 0·87 (0·83 - 0·91) | 0·87 (0·78 - 0·97) |  | 0·96 (0·90 - 1·02) | 1·08 (0·98 - 1·19) |  | 0·98 (0·93 - 1·03) | 0·95 (0·86 - 1·04) |
|  | 2010 | 1·00 (Reference) | 1·00 (Reference) |  | 1·00 (Reference) | 0·97 (0·89 - 1·06) |  | 1·00 (Reference) | 1·00 (Reference) |
|  | 2015 | 1·06 (1·02 - 1·10) | 1·05 (0·96 - 1·15) |  | 1·20 (1·13 - 1·26) | 1·04 (0·96 - 1·12) |  | 1·11 (1·06 - 1·16) | 0·97 (0·89 - 1·06) |
|  | 2020 | 1·04 (1·00 - 1·08) | 0·96 (0·87 - 1·05) |  | 1·21 (1·15 - 1·28) | 0·97 (0·90 - 1·05) |  | 1·10 (1·05 - 1·16) | 0·89 (0·81 - 0·97) |
|  |  | **RR (95%CI)** | **RR (95%CI)** |  | **RR (95%CI)** | **RR (95%CI)** |  | **RR (95%CI)** | **RR (95%CI)** |
| **Birth Cohort** | 1920 | 0·58 (0·49 - 0·69) | 0·91 (0·68 - 1·23) |  | 0·71 (0·54 - 0·92) | 0·26 (0·16 - 0·43) |  | 0·61 (0·50 - 0·75) | 1·00 (0·78 - 1·28) |
|  | 1925 | 0·77 (0·70 - 0·85) | 0·95 (0·78 - 1·14) |  | 0·79 (0·67 - 0·91) | 0·66 (0·49 - 0·89) |  | 0·76 (0·67 - 0·85) | 1·28 (1·08 - 1·52) |
|  | 1930 | 0·81 (0·75 - 0·87) | 1·07 (0·95 - 1·22) |  | 0·86 (0·77 - 0·96) | 0·79 (0·62 - 1·01) |  | 0·83 (0·75 - 0·91) | 1·18 (1·02 - 1·36) |
|  | 1935 | 0·85 (0·80 - 0·90) | 0·96 (0·86 - 1·06) |  | 0·87 (0·80 - 0·95) | 0·78 (0·63 - 0·97) |  | 0·83 (0·76 - 0·89) | 1·03 (0·90 - 1·18) |
|  | 1940 | 0·91 (0·87 - 0·96) | 1·00 (0·91 - 1·09) |  | 0·84 (0·78 - 0·91) | 0·80 (0·65 - 0·98) |  | 0·81 (0·76 - 0·87) | 1·11 (0·98 - 1·26) |
|  | 1945 | 0·88 (0·84 - 0·93) | 0·98 (0·90 - 1·08) |  | 0·86 (0·79 - 0·92) | 0·82 (0·68 - 0·98) |  | 0·89 (0·83 - 0·95) | 0·98 (0·86 - 1·11) |
|  | 1950 | 0·94 (0·90 - 0·99) | 1·02 (0·93 - 1·11) |  | 0·93 (0·87 - 1·00) | 0·89 (0·75 - 1·05) |  | 0·92 (0·86 - 0·97) | 0·94 (0·83 - 1·07) |
|  | 1955 | 1·00 (Reference) | 1·04 (0·96 - 1·14) |  | 1·00 (Reference) | 1·00 (Reference) |  | 1·00 (Reference) | 1·00 (Reference) |
|  | 1960 | 1·00 (0·96 - 1·05) | 0·99 (0·90 - 1·09) |  | 0·95 (0·90 - 1·02) | 1·19 (1·01 - 1·41) |  | 0·96 (0·91 - 1·02) | 1·04 (0·91 - 1·19) |
|  | 1965 | 0·87 (0·82 - 0·92) | 1·02 (0·91 - 1·14) |  | 0·87 (0·81 - 0·94) | 1·25 (1·03 - 1·51) |  | 0·89 (0·83 - 0·95) | 1·09 (0·94 - 1·27) |
|  | 1970 | 0·77 (0·72 - 0·82) | 1·01 (0·87 - 1·18) |  | 0·78 (0·72 - 0·85) | 1·21 (0·95 - 1·53) |  | 0·77 (0·71 - 0·84) | 0·96 (0·80 - 1·16) |
|  | 1975 | 0·70 (0·63 - 0·77) | 0·88 (0·69 - 1·12) |  | 0·75 (0·66 - 0·84) | 1·12 (0·81 - 1·54) |  | 0·63 (0·57 - 0·71) | 1·29 (1·02 - 1·63) |
|  | 1980 | 0·58 (0·49 - 0·69) | 0·70 (0·43 - 1·12) |  | 0·66 (0·55 - 0·80) | 1·19 (0·74 - 1·92) |  | 0·55 (0·46 - 0·67) | 1·44 (0·98 - 2·10) |
