# Supplementary material for: Head and neck cancer mortality by gender, region and ethnicity: a population-based study in Brazil
Source: Lancet Reg Health Am. 2025 Nov 29;53:101306. doi: 10.1016/j.lana.2025.101306 (PMC12703860; doi:10.1016/j.lana.2025.101306)
Supplement: Translated Summary [file mmc2.docx]

***Editorial disclaimer*:** *This translation in Portuguese was submitted by the authors and we reproduce it as supplied. It has not been peer reviewed. Our editorial processes have only been applied to the original abstract in English, which should serve as reference for this manuscript*

RESUMO

Introdução: O Brasil apresenta as maiores taxas de mortalidade por câncer de cavidade oral (CCO), orofaringe (CO) e laringe (CL) da América do Sul. Este estudo teve como objetivo analisar as tendências de mortalidade por câncer de cabeça e pescoço (CCP) na população brasileira ao longo de um período de 44 anos.

Métodos: Estudo ecológico de séries temporais utilizando dados de mortalidade por CCO, CO e CL. Foram estimados os efeitos de idade-período-coorte e a variação percentual média anual (*average annual percent change* – AAPC) para cada subsítio anatômico, segundo gênero, região e etnia.

Resultados: Entre 1980 e 2023, foram registrados 303.882 óbitos por CCP entre adultos ≥40 anos. O CL predominou (45,4%), seguido por CCO (30,5%) e CO (24,1%). As taxas de mortalidade foram mais altas em homens, com CL apresentando as maiores. Após 2000, observou-se declínio da mortalidade por CL em homens (RR: 0,98 [IC95% 0,98–1,00]), enquanto as mulheres apresentaram aumento na mortalidade por CO (RR: 1,12 [1,04–1,21]) e CCO (RR: 1,08 [1,02–1,15]). Homens nascidos após 1955 mostraram redução do risco relativo (RR) para todos os subsítios, enquanto entre as mulheres essa redução foi observada apenas para CL. A região Nordeste apresentou tendência crescente em todos os grupos etários e subsítios. O CO mostrou aumento nas tendências de mortalidade em todas as regiões, enquanto CL e o CCO diminuíram no Sul e Sudeste. Homens brancos apresentaram tendência decrescente para CL (AAPC = –1,62 [–1,99 – –1,26]), CCO (AAPC = –1,01 [–1,31 – –0,71]) e CO (AAPC = –0,60 [–0,98 – –0,24]), enquanto pardos mostraram tendência crescente em ambos os sexos e em todos os subsítios.

Interpretação: Gênero, etnia e localização geográfica estão associados à mortalidade por CCP no Brasil. Homens brancos e regiões mais desenvolvidas apresentaram as maiores reduções nas taxas de mortalidade, enquanto pardos, mulheres e residentes nas regiões Norte e Nordeste exibiram aumentos preocupantes. O CO, embora com as menores taxas absolutas, mostra tendência crescente de mortalidade em todo o país.

Financiamento: CAPES–Brasil.
